# Ku-Mediated Coupling of DNA Cleavage and Repair during Programmed Genome Rearrangements in the Ciliate *Paramecium tetraurelia*

**DOI:** 10.1371/journal.pgen.1004552

**Published:** 2014-08-28

**Authors:** Antoine Marmignon, Julien Bischerour, Aude Silve, Clémentine Fojcik, Emeline Dubois, Olivier Arnaiz, Aurélie Kapusta, Sophie Malinsky, Mireille Bétermier

**Affiliations:** 1 CNRS UPR3404 Centre de Génétique Moléculaire, Gif-sur-Yvette, France; Université Paris-Sud, Département de Biologie, Orsay, France; 2 Ecole Normale Supérieure, Institut de Biologie de l'ENS, IBENS, Paris, France; INSERM, U1024, Paris, France; CNRS, UMR 8197, Paris, France; 3 Université Paris Diderot, Sorbonne Paris Cité, UFR Sciences du Vivant, Paris, France; The University of North Carolina at Chapel Hill, United States of America

## Abstract

During somatic differentiation, physiological DNA double-strand breaks (DSB) can drive programmed genome rearrangements (PGR), during which DSB repair pathways are mobilized to safeguard genome integrity. Because of their unique nuclear dimorphism, ciliates are powerful unicellular eukaryotic models to study the mechanisms involved in PGR. At each sexual cycle, the germline nucleus is transmitted to the progeny, but the somatic nucleus, essential for gene expression, is destroyed and a new somatic nucleus differentiates from a copy of the germline nucleus. In *Paramecium tetraurelia*, the development of the somatic nucleus involves massive PGR, including the precise elimination of at least 45,000 germline sequences (Internal Eliminated Sequences, IES). IES excision proceeds through a cut-and-close mechanism: a domesticated transposase, PiggyMac, is essential for DNA cleavage, and DSB repair at excision sites involves the Ligase IV, a specific component of the non-homologous end-joining (NHEJ) pathway. At the genome-wide level, a huge number of programmed DSBs must be repaired during this process to allow the assembly of functional somatic chromosomes. To understand how DNA cleavage and DSB repair are coordinated during PGR, we have focused on Ku, the earliest actor of NHEJ-mediated repair. Two Ku70 and three Ku80 paralogs are encoded in the genome of *P. tetraurelia*: Ku70a and Ku80c are produced during sexual processes and localize specifically in the developing new somatic nucleus. Using RNA interference, we show that the development-specific Ku70/Ku80c heterodimer is essential for the recovery of a functional somatic nucleus. Strikingly, at the molecular level, PiggyMac-dependent DNA cleavage is abolished at IES boundaries in cells depleted for Ku80c, resulting in IES retention in the somatic genome. PiggyMac and Ku70a/Ku80c co-purify as a complex when overproduced in a heterologous system. We conclude that Ku has been integrated in the *Paramecium* DNA cleavage factory, enabling tight coupling between DSB introduction and repair during PGR.

## Author Summary

DNA double-strand breaks (DSBs) are potential threats for chromosome stability, but they are usually repaired by two major pathways, homologous recombination or non-homologous end joining (NHEJ). DSBs can also be essential during physiological processes, such as the programmed removal of germline sequences that takes place in various eukaryotes, including ciliates, during somatic differentiation. We use the ciliate *Paramecium tetraurelia* as a unicellular model to study how DNA breakage and DSB repair are coordinated during programmed genome rearrangements. In this organism, assembly of the somatic genome involves the elimination of ∼25% of germline DNA, including the precise excision of thousands of short Internal Eliminated Sequences (IES) scattered along germline chromosomes. A domesticated piggyBac transposase, PiggyMac, is required for double-strand DNA cleavage at IES ends and IES excision sites are very precisely repaired by the NHEJ pathway. Here, we report that a specialized Ku heterodimer, specifically expressed during programmed genome rearrangements, is an essential partner of PiggyMac and activates DNA cleavage. We propose that incorporation of DSB repair proteins in a pre-cleavage complex constitutes a safe and efficient way for *Paramecium* to direct thousands of programmed DSBs to the NHEJ pathway and make sure that somatic chromosomes are assembled correctly.

## Introduction

DNA double strand breaks (DSBs) are among the most deleterious DNA lesions: if left unrepaired, a single DSB may trigger cell death, while incorrect repair can give rise to chromosome rearrangements [1]. Cells rely on two major pathways to repair DSBs. Homologous recombination (HR) uses a homologous template to restore the sequence of the broken chromosome, while non-homologous end joining (NHEJ) proceeds through the ligation of free DNA ends. Even though they can be very toxic, programmed DSBs are obligatory intermediates in essential biological processes, such as meiosis or acquired immune response. During meiosis, the Spo11 endonuclease cleaves DNA and DSB repair is carried out by HR [2]. In addition to favoring the exchange of parental alleles, HR ensures that homologous chromosomes are correctly paired before they are segregated during the first meiotic division. During lymphocyte differentiation, programmed genome rearrangements (PGR) mediated through V(D)J recombination generate the large diversity of immunoglobulin genes [3]. During V(D)J recombination, the domesticated transposase RAG1, associated with its partner RAG2, cleaves specific recombination sites. The resulting DSBs are repaired through classical NHEJ (C-NHEJ).

A critical step in C-NHEJ is the binding of the Ku70/Ku80 heterodimer to broken DNA ends [4]. Upon binding, Ku protects DNA ends from extensive resection [5] and, together with its facultative partner DNA-PKcs, facilitates the synapsis of two broken ends. Following recruitment of DNA processing enzymes, the Ligase IV-Xrcc4 complex mediates the joining of DNA ends. An alternative end joining pathway, referred to as alt-NHEJ (or MMEJ, for microhomology-dependent end joining), has been reported [6]. This poorly characterized pathway is independent of Ku and, to some extent, of Ligase IV. Because of the absence of Ku, alt-NHEJ involves limited 5′ to 3′ resection of broken DNA ends and generates deletions at DSB repair sites, which often involve microhomologies. When DSBs are repaired through HR, 5′ to 3′ resection also takes place, but two steps can be distinguished: initial short-range end resection relies on the same factors as alt-NHEJ [7], while subsequent long-range resection generates the long 3′ single strand that will invade a homologous DNA duplex [8].

Ciliates provide extraordinary models to study the interplay between DNA cleavage and DSB repair during PGR [9]. In these unicellular eukaryotes, two kinds of nuclei coexist in the same cytoplasm. The highly polyploid somatic macronucleus (MAC) is essential for gene expression but it is destroyed at each sexual cycle, while the diploid micronucleus (MIC) undergoes meiosis and transmits the germline genome to the new MIC and MAC of the next generation. In *Paramecium*, massive PGR take place in the new developing MAC, while the genome is amplified from 2n to 800n [10]. These PGR consist in the elimination of two types of germline-specific DNA. Regions of up to several kbp in length, often containing repeated sequences, are eliminated in a heterogeneous manner, leading to chromosome fragmentation or intra-chromosomal deletions. In addition, thousands of single-copy, short and non-coding Internal Eliminated Sequences (IES) are excised precisely. Because 47% of genes are interrupted by at least one IES in the germline genome [11], the precise excision of IESs is essential for the assembly of functional genes in the new MAC and the survival of the sexual progeny. *Paramecium* IESs are invariably flanked by one TA dinucleotide on each side and little additional information can be found in their nucleotide sequence, which raises the question of how these sequences are recognized and targeted for excision. In fact, the excision of an estimated one-third of *Paramecium* IESs is controlled maternally through a sequence homology-dependent mechanism [12]. For these so-called maternally-controlled IESs, a genome-wide comparison of the germline and rearranged versions of the genome involves non-coding RNAs [13], [14]. This epigenetic control drives the trans-generational inheritance of rearrangement patterns, from the old to the new MAC.

IES excision proceeds through a two-step “cut-and-close” mechanism. A domesticated *piggyBac* transposase, PiggyMac (Pgm), is essential to introduce the DNA cleavages that initiate the reaction, generating 4-bp staggered DSBs centered on the conserved TA at each IES boundary [15]. Following IES release, precise DSB repair is carried out through the C-NHEJ pathway and leaves a single TA at the IES excision site [16]. Providing their length allows enough DNA flexibility, the excised linear IESs are circularized, also through the C-NHEJ pathway, before they are actively degraded. During DSB repair, the flanking broken ends are thought to anneal through the pairing of the complementary TAs carried by their 4-base 5′ overhangs, and undergo limited 5′ and 3′ processing [17]. The final ligation step is mediated by the NHEJ-specific ligase complex, Ligase IV and its partner Xrcc4, both of which are essential for PGR. When *LIG4* or *XRCC4* genes are knocked down, Pgm-dependent DSBs are introduced normally, but they accumulate in the developing new MAC, which correlates with severely compromised DNA amplification and impairs the recovery of viable progeny [16].

Next-generation sequencing of the non-rearranged genome of *Paramecium tetraurelia* led to the identification of at least 45,000 IESs [11]. Therefore, a huge number of programmed DSBs have to be repaired precisely during MAC development. In the present study, we have addressed the question of how the C-NHEJ pathway is recruited to IES excision sites to carry out efficient and precise DSB repair. We have focused our analysis on the Ku heterodimer, which is the most upstream actor of the C-NHEJ pathway. Two *KU70* and three *KU80* genes were identified in the somatic genome [16]. We report here that development-specific *KU* genes are essential for PGR. Surprisingly, we demonstrate that Ku is required for the introduction of programmed DSBs at IES boundaries, and provide evidence that Ku interacts functionally and physically with Pgm. We propose that the Ku70a/Ku80c heterodimer forms a complex with Pgm and activates DNA cleavage during PGR in *P. tetraurelia*.

## Results

### Development-specific *KU* genes in *P. tetraurelia*

**Figure 1:**
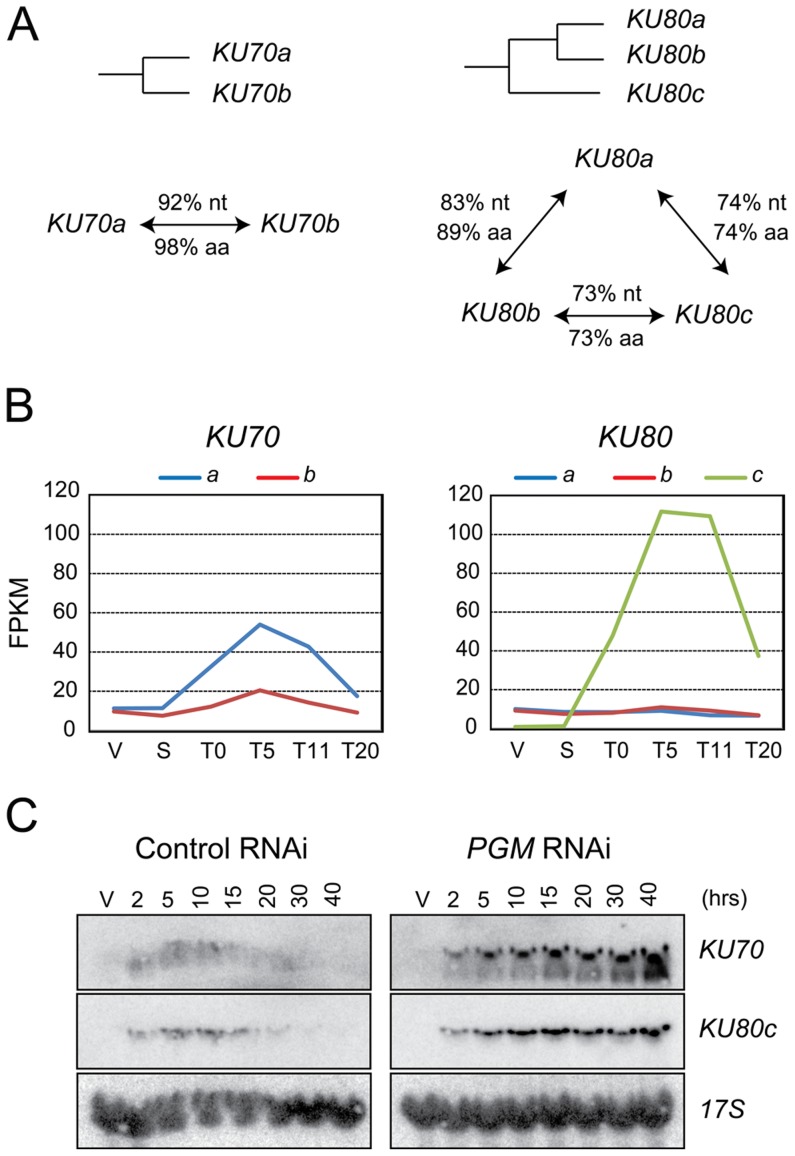
*KU70* and *KU80* genes in *P. tetraurelia*. (A) Diagram of the WGD relationships between *KU70* or *KU80* genes in *P. tetraurelia*. The % of identity between genes (nt) and proteins (aa) are indicated along the arrows connecting two genes. (B) Transcription profiles of *KU70* and *KU80* genes during an autogamy time-course of strain 51, as determined by high-throughput RNA-seq. V: vegetative cells; S: starved or meiotic cells with intact parental MAC; T0: 50% of cells with fragmented MAC; the following time-points refer to hours after T0 [51]. On the vertical axis, FPKM represents the number of fragments per gene kb per million of fragments that were uniquely mapped on the genome. (C) Detection of *KU70* and *KU80c* mRNA during autogamy, through northern blot hybridization. Control RNAi: RNAi against the nonessential *ND7* gene [43], which encodes an exocytosis protein. V: vegetative cells. The times refer to hours after T0, the time at which 50% of cells have a fragmented MAC (Figure S1A).

Two genes encoding Ku70 homologs, *KU70a* and *KU70b*, were identified in the macronuclear genome assembly of *P. tetraurelia* (Figure 1A and [16]). These two closely related copies arose from a recent whole genome duplication (WGD) that took place during the evolution of this species [18], and are referred to as ohnologs. We also identified three homologs of the human *KU80* gene (Figure 1A). *KU80a* and *KU80b* are ohnologs from the recent WGD, while the more distant *KU80c* diverged after an earlier, “intermediate”, WGD.

Sexual processes in *Paramecium* may occur in two different ways: during conjugation, following the mixing of two reactive partners with compatible mating types, or during a self-fertilization process called autogamy, in which MIC meiosis is induced upon starvation in cells of a single mating type [19]. A microarray analysis of the *P. tetraurelia* transcriptome during autogamy revealed that *KU70a* and *KU80c* are specifically induced during the development of the new MAC, when PGR take place [16], [20]. The microarray data were confirmed by high-throughput sequencing of polyadenylated RNAs extracted during an autogamy time-course (Figure 1B, Arnaiz et al. in prep.), and by northern blot hybridization with specific probes (Figures 1C and 3C, left panels; Malinsky et al. in prep.). After Pgm depletion, the transcription of *KU70* and *KU80c* is switched on at the expected time-point during autogamy (Figure 1C, right panels), indicating that *KU* genes are not induced as a response to Pgm-induced DSBs, but more likely as part of a general transcription program during MAC development. Moreover, in contrast to control cells, the levels of *KU70* and *KU80c* mRNAs do not decrease at later time-points after Pgm depletion, suggesting that the completion of PGR is a signal for transcriptional switch-off.

### Ku70a and Ku80c localize in the developing new MAC

**Figure 2:**
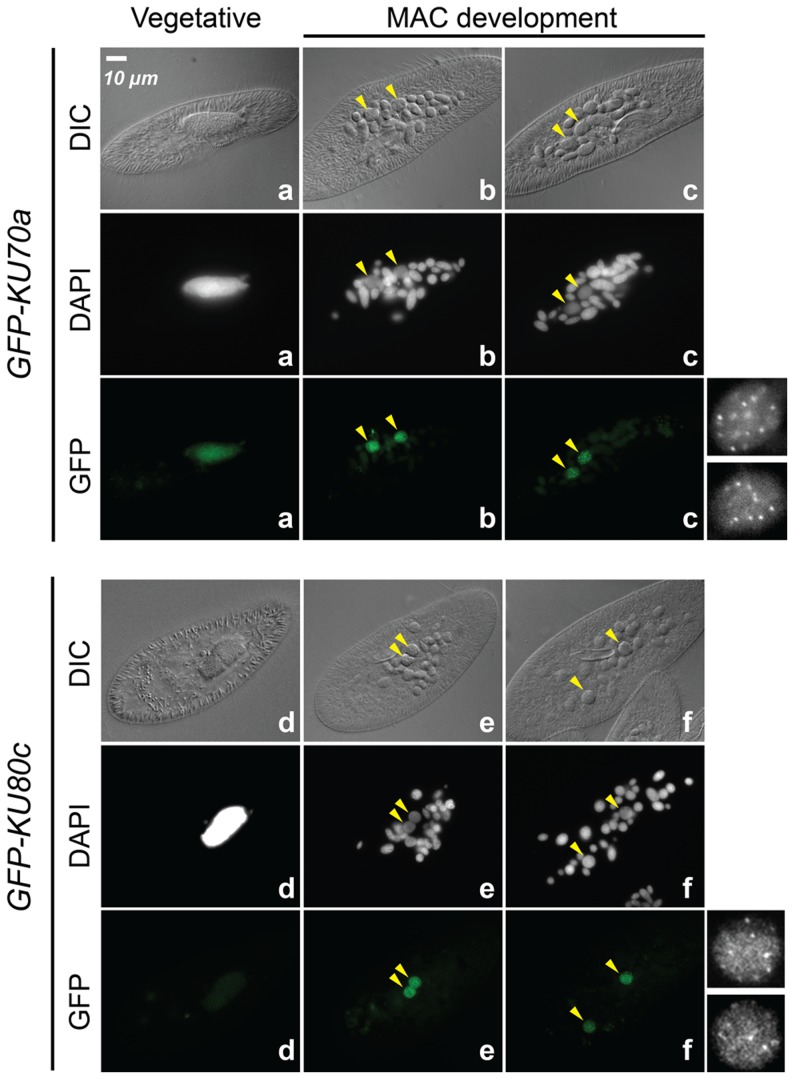
Nuclear localization of GFP-Ku fusions during autogamy. Cells were microinjected with fusion transgenes expressing GFP-Ku70a (panels a, b, c) and GFP-Ku80c (panels d, e, f) under the control of their respective transcription signals. In autogamous cells, developing MACs are indicated by yellow arrowheads, the other DAPI-stained nuclei are fragments from the old vegetative MAC. For each protein, the GFP fluorescence sometimes concentrated in nuclear foci of unclear biological significance (see cells in panels c and f, and enlarged inserts on the right). In this particular experiment, expression of the GFP fusions had no significant effect on the recovery of viable post-autogamous progeny (87% progeny with functional new MACs for GFP-Ku70a, 90% for GFP-Ku80c).

The expression of *KU70a* and *KU80c* is specifically induced during autogamy and reaches a peak when the developing new MACs start to be detected in the culture (T5 and T11 time-points in Figure 1B). Using N-terminal GFP fusions, we followed the cellular localization of Ku70a and Ku80c during autogamy. Transgenes expressing each fusion protein under the control of their respective endogenous promoters were microinjected into the MAC of vegetative cells. The resulting transformants were grown and starved to induce autogamy. Consistent with the transcriptome analysis, GFP fluorescence was stronger in autogamous cells with respect to vegetative cells for each fusion transgene (Figure 2). Both fusion proteins accumulated in the developing new MACs, suggesting a possible involvement of Ku70a and Ku80c in PGR during MAC development.

**Figure 3:**
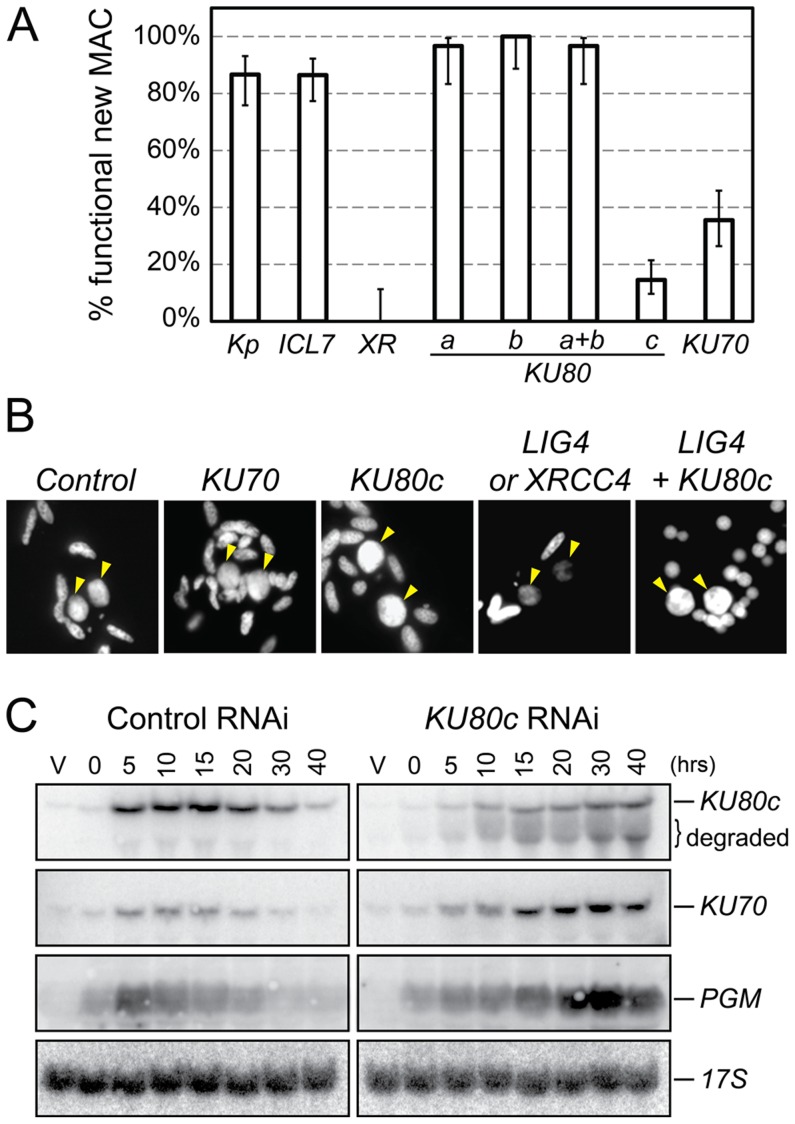
RNAi screen for essential *KU* genes during autogamy. (A) Survival of the post-autogamous progeny of cells submitted to different combinations of RNAi. Kp: autogamy in standard *K. pneumoniae* medium; *ICL7*: RNAi against *ICL7*, a nonessential gene that encodes an infraciliary lattice centrin [47]; *XR*: RNAi against *XRCC4*. RNAi experiments against *KU80* genes were performed using gene-specific inserts KU80-a2, KU80-b2 and KU80-c2 (see Figure S2). For each condition, 30 to ∼140 post-autogamous cells were analyzed. Each bar represents the percentage of viable post-autogamous cells carrying a functional new MAC, for each condition. Error bars represent the Wilson score intervals (95% confidence level), which are appropriate for a small number of trials or for values close to an extreme probability. (B) DAPI-staining of developing MACs during RNAi against *ND7* (control), *KU70*, *KU80c*, *XRCC4/LIG4* and *LIG4* + *KU80c*. Developing MACs are indicated by yellow arrowheads. Upon *LIG4* or *XRCC4* RNAi, developing MACs exhibit faint DAPI staining, which correlates with a defect in DNA amplification [16]. (C) Northern blot hybridization of total RNA during a control time course experiment (*ND7* RNAi) and in a *KU80c* RNAi. V: vegetative cells; T0: 60% of cells with fragmented MAC; other time-points refer to hours following T0 (Figure S1B).

### Ku70 and Ku80c are required for successful completion of autogamy

To test the implication of the different *KU* genes in MAC development, we knocked them down systematically by feeding wild-type *P. tetraurelia* cells on dsRNA-producing bacteria to induce RNA interference [21]. The very high percentage of identity between *KU70a* and *KU70b* made it impossible to design specific RNAi constructs for each individual gene. Therefore, we silenced them both together. In contrast, we designed gene-specific RNAi constructs for *KU80a*, *KU80b* and *KU80c*. Whenever possible, to make sure that the observed phenotypes would be attributable to the silencing of each targeted gene, we used RNAi constructs homologous to two different regions in each *KU80* gene (Figure S2). After three days of starvation in each silencing medium, individual autogamous cells were transferred to standard growth medium and allowed to resume vegetative growth. The survivors were grouped in three categories: (i) those that were able to undergo a new round of autogamy following a few divisions, most likely because they had regenerated their old MAC, were not considered as *bona fide* post-autogamous progeny [16]; (ii) slow-growing survivors were counted as progeny with a defective new MAC; (iii) those survivors that remained in the vegetative state when starved after a few divisions were classified as fully viable post-autogamous progeny. Only the recovery of survivors from the third category indicated that the silenced cells had been able to form a functional new MAC (Figure 3A).

When *KU80a* or *KU80b* were knocked down, either individually or together, the progeny exhibited good survival rates when compared to a control RNAi against the nonessential *ICL7* gene, or with respect to cells that underwent autogamy in standard medium (Figure 3A). In contrast, RNAi against *KU80c* or *KU70* yielded only 10 to 30% viable sexual progeny, showing that the developmentally-induced *KU80c* gene and one or both *KU70* genes are required for the completion of autogamy. Interestingly, we observed that new MACs develop normally at the cytological level in cells silenced for *KU70* or *KU80c* (Figure 3B), while cells silenced for *LIG4* or *XRCC4* were previously shown to harbor small new developing MACs that remain faintly stained with DAPI, correlating with a block in DNA amplification [16]. Bright DAPI staining of the new MACs indicates that DNA amplification takes place normally in Ku-depleted cells.

Residual survival was observed reproducibly following Ku70 or Ku80c depletions, in contrast to the severe lethality phenotype of Xrcc4 depletion (Figure 3A). This phenotypic difference, which was also noted during mouse embryonic development [22], may be explained by the fact that Ku acts upstream of Ligase IV/Xrcc4 during C-NHEJ. Upon Ku depletion, alternative DSB repair pathways could restore chromosome integrity to some extent, while Ligase IV/Xrcc4 depletion would constitute a dead-end for DSBs, committed to C-NHEJ in the presence of Ku. We noted, indeed, that normal DAPI staining of the developing MAC is restored in Ligase IV-depleted cells by simultaneously depleting Ku80c (Figure 3B). At this stage of our study, this observation is consistent with the hypothesis that activation of alternative Ku-independent DSB repair pathways may rescue DNA amplification in the new MAC, therefore allowing the recovery of low amounts of post-autogamous survivors. An alternative explanation for the residual survival observed upon Ku80c depletion could also be that *KU80c* transcripts are only partially degraded by RNAi, as testified by the restoration of significant amounts of full-length mRNA at T30 and T40 (Figure 3C, top lane).

### *KU70* and *KU80c* are essential for the programmed elimination of germline DNA

We showed previously that the NHEJ-specific Ligase IV/Xrcc4 ligation complex joins broken DNA ends at IES excision sites [16]. Strikingly, after Ligase IV depletion, unrepaired broken ends remain very stable throughout autogamy, suggesting that Ku protects them against degradation. To gain further insight into the role of Ku during IES excision, we performed a molecular analysis of PGR in Ku-depleted cells. As predicted from the known role of Ku during C-NHEJ, we expected that DSBs would be introduced normally at IES boundaries, but that the broken DNA ends would not be directed to the C-NHEJ pathway.

**Figure 4:**
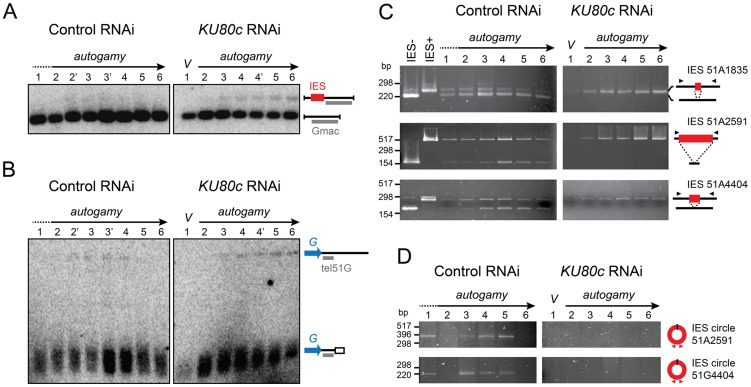
Molecular analysis of genome rearrangements after Ku80c depletion. (A) Detection of DNA fragments with excised or non-excised IES 51G4404 from the surface antigen *G^51^* gene. Total genomic DNA was extracted during autogamy of 51ΔA cells subjected to RNAi against *ICL7* (control) or *KU80c*. To compare the two time-courses, similar autogamy stages were numbered from 1 to 6, based on the observation of DAPI-stained cells (Figure S1C). *Pst*I-hydrolyzed total genomic DNA was run on 1% agarose gels. Southern blots were hybridized with the Gmac probe (in grey), which hybridizes to the flanking MAC DNA downstream of the IES. (B) Detection of fragmented or non-fragmented DNA downstream of the *G^51^* gene by Southern blot hybridization of *Pst*I-digested total genomic DNA (same samples as in A) run on 0.8% agarose gels. The subtelomeric tel51G probe is shown in grey. The white box in the bottom right diagram represents telomeric repeats. (C) PCR detection of *de novo* IES excision junctions during autogamy of 51ΔA cells, in an *ICL7* (control) or a *KU80c* RNAi. Note that a few autogamous cells were present at time-point 1 in the control RNAi (see Figure S1C). (D) PCR detection of IES circle junctions during autogamy in an *ICL7* (control) or a *KU80c* RNAi. Triangles in C and D represent PCR primers (see Table S1).

We first focused our analysis on cells depleted in Ku80c. Using Southern blot hybridization, we monitored the excision of one particular IES, IES 51G4404 from the surface antigen *G^51^* gene [23], during an autogamy time-course (Figure 4A). In a control RNAi (left panel), a major band corresponded to the IES^−^ molecules from the old MAC and the newly rearranged molecules from the developing new MAC. A higher molecular weight species corresponding to the non-excised IES^+^ form was transiently and only barely detectable during MAC development (lanes 2′ to 4), and was completely absent at late time-points as a result of IES excision. In contrast, the IES^+^ form was clearly amplified during autogamy after Ku80c depletion (right panel). Using a related strategy, we tested chromosome fragmentation at one particular fragmentation site located downstream of the *G^51^* gene (Figure 4B). We observed that non-rearranged molecules are amplified during MAC development in Ku80c-depleted cells, which indicates that Ku is also required for the elimination of the germline DNA that is associated with chromosome fragmentation.

The amplification of non-rearranged DNA indicates that PGR do not proceed normally after Ku80c depletion. However, because of the presence of old MAC DNA in our samples, the above experiment could not detect whether residual rearrangements had taken place in the new MAC. We could circumvent this problem, because the strain used in this experiment (51ΔA) harbors a wild-type germline genome, but carries a somatic deletion of the nonessential surface antigen gene *A^51^*. During autogamy, all IESs are excised normally from the *A^51^* gene before the whole locus is deleted from the new somatic MAC [24]. Therefore, in the 51ΔA variant, all the IES^−^ molecules originating from this locus may be attributed to *de novo* IES excision from the new MAC. Using PCR primers hybridizing in the flanking sequences, excised (IES^−^) and non-excised (IES^+^) molecules were readily detected in a control RNAi experiment, for IESs belonging to different classes (Figure 4C, left panel): short (51A1835: 28 bp), intermediate (51A4404: 77 bp) or long (51A2591: 370 bp), maternally (51A2591) or non-maternally controlled (51A1835 and 51A4404). This stands in sharp contrast to the complete absence of *de novo* IES excision junctions after Ku depletion (right panel). Likewise, using IES-specific internal divergent primers, we followed the appearance of excised IES circular molecules during the autogamy of control and *KU80c*-silenced cells, but no circle junctions were detected following Ku80c depletion (Figure 4D).

To extend our analysis to the other *KU* genes, we submitted small-scale cultures of 51ΔA cells to RNAi against *KU70* or individual *KU80* genes, and tested their ability to complete IES excision during autogamy. When *KU80a* and *KU80b* were knocked down, together or separately, rearranged IES^−^ molecules appeared at day 2 of starvation, with the same timing as in a control RNAi (Figure S3). In contrast, the appearance of *de novo* IES excision junctions was strongly impaired upon Ku70 depletion, similar to Ku80c depletion. Taken together, our molecular data indicate that Ku70 and the development-specific Ku80c are essential for the recovery of both precise chromosomal junctions and excised IES circles during PGR. Moreover, after Ku70 or Ku80c depletion, the non-rearranged version of the genome is amplified in the developing MAC, a strikingly different phenotype from that of Ligase IV or Xrcc4 depletions [16], but quite similar to Pgm depletion [15].

### No DNA breaks can be detected by LMPCR in cells depleted for Ku80c

**Figure 5:**
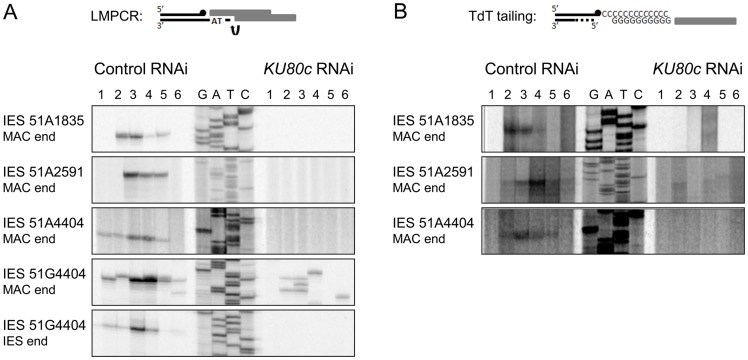
Detection of programmed DSBs at IES boundaries during autogamy. (A) LMPCR detection of DSBs at MAC or IES ends during autogamy of 51ΔA cells subjected to RNAi against *ICL7* (control) or *KU80c* (same samples as in Figure 4). A Sanger DNA sequencing ladder provides size markers. On the diagram, the LMPCR linker is drawn as grey boxes and *Paramecium* DNA as black lines, with a black dot representing the 3′ end generated by Pgm-dependent cleavage. In the *KU80c* RNAi, the LMPCR signals at 51G4404 MAC ends are likely due to background DNA breaks generated at the MAC *G^51^* locus during DNA extraction. This background is not detected for IESs of the *A^51^* gene, because this locus is absent from the old MAC. (B) TdT tailing of free 3′OH ends during autogamy of 51ΔA cells subjected to RNAi against *ICL7* (control) or *KU80c* (same samples as in (A)). On the diagram, the potentially resected 5′ end is represented by a dotted line.

The absence of precise *de novo* IES excision junctions after Ku70 or Ku80c depletions could either reflect a problem in DSB repair, as established for Ligase IV depletion, or an inhibition of DNA cleavage, as demonstrated for Pgm depletion. Because Ku is essential for C-NHEJ in all organisms, a defect in end-joining was expected after Ku depletion in *P. tetraurelia*. However, the observation that non-excised IESs were amplified in the new MAC of cells depleted for Ku70/Ku80c raised the issue of whether DSBs were actually introduced at IES boundaries. We therefore used a sensitive ligation-mediated PCR assay (LMPCR) to search for DSBs at IES boundaries after Ku depletion. As previously published [17], for those IESs that were tested, free broken DNA ends at IES boundaries were detected at early autogamy time-points in cells subjected to a control RNAi (Figure 5A). DSBs disappeared later on during MAC development, indicative of efficient repair. After Ku80c depletion, no specific broken ends were detected on the MAC side or the IES side of the DSB (Figure 5A).

**Figure 6:**
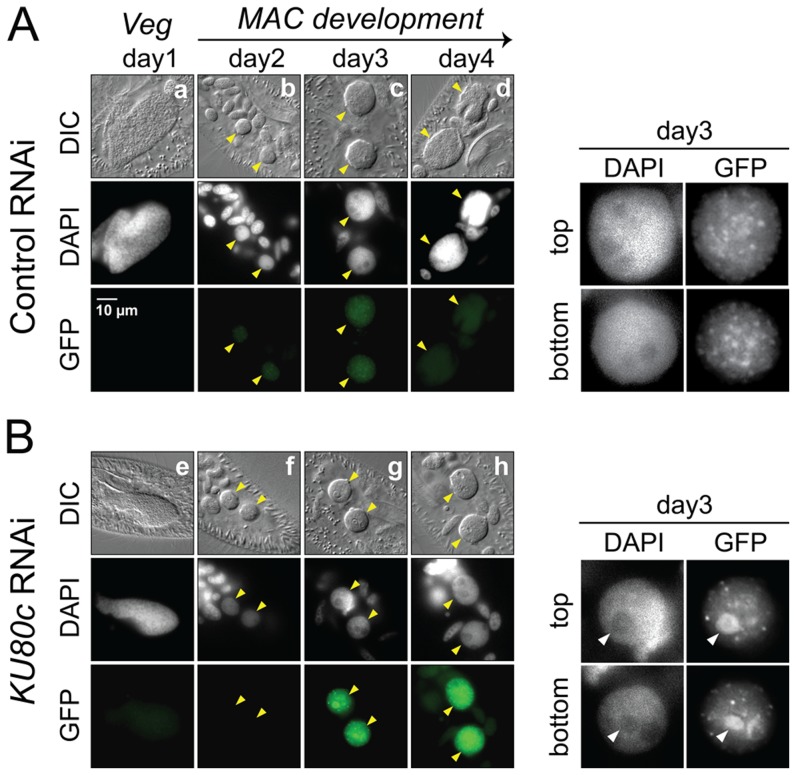
Nuclear accumulation of a Pgm-GFP fusion in Ku80c-depleted cells. Cells were microinjected with a *PGM-GFP* fusion transgene and one transformant was submitted to RNAi against *ICL7* (control: panel A) or *KU80c* (panel B). The progression of autogamy was monitored over a four-day starvation period (a and e: day 1; b and f: day 2; c and g: day 3; d and h: day 4). To compare the intensities of GFP fluorescence, signals were acquired with the same exposure time, and identical window settings were applied to the image display using the ImageJ software (National Institute of Health). Developing MACs are indicated by yellow arrowheads. In the enlarged inserts shown on the right of each panel, the display settings were modified to highlight the Pgm-GFP nuclear foci. The white arrowheads in panel B point to the DAPI-free regions, in which overproduced Pgm-GFP accumulates following *KU80c* RNAi. In the control RNAi, 93% of post-autogamous progeny had a functional new MAC, while the *KU80c* RNAi yielded no progeny with a functional new MAC.

An important control was to verify whether Pgm is produced and imported normally into the developing MAC upon Ku depletion. Cells expressing a Pgm-GFP fusion under the control of the endogenous *PGM* transcription signals were subjected to a control RNAi or to RNAi against *KU80c* (Figure 6). In the control, the fusion protein appeared in the developing new MAC at day 2 of starvation, and formed small foci until day 3 (Figure 6A). Pgm-GFP foci disappeared at day 4, which corresponds to the time when most PGR are completed in control cells (see Figure S3). In a *KU80c* RNAi, the Pgm-GFP fusion also localized specifically to the developing new MAC (Figure 6B), indicating that Ku is not required for the nuclear import of Pgm. However, Ku80c depletion triggered a dramatic increase in the nuclear amount of the Pgm-GFP fusion (Figure 6B), which parallels the accumulation of the endogenous *PGM* mRNA observed upon *KU80c* RNAi in cells harboring no fusion transgene (Figure 3C, right panels). We also observed a striking difference in the subnuclear localization of Pgm, in a *KU80c* RNAi relative to the control. Indeed, at day 3, Pgm-GFP accumulated in large nuclear bodies, which were clearly detectable under differential interference contrast (DIC) and coincided with DAPI-free regions (Figure 6B, right panels). At day 4, GFP fluorescence was still high, but large nuclear bodies were no longer detectable. The apparently normal subnuclear organization of Pgm-GFP observed at day 4 correlated with the recovery of functional amounts of *KU80c* mRNA at very late time-points (Figure 3C, top right panel).

### No detectable DSB repair intermediates in Ku-depleted cells

In other organisms, Ku is known to protect broken DNA ends against degradation [1], and Ku depletion may reveal alternative DSB repair pathways, such as alt-NHEJ or HR, by allowing 5′ to 3′ DNA end resection. Should resection occur upon Ku depletion in *P. tetraurelia*, the resected 5′ DNA ends would not be appropriate substrates for the LMPCR assay displayed in Figure 5, because the linkers that were used only allow detection of DSBs with a specific geometry (4- or 3-base 5′ overhangs). We first investigated whether alt-NHEJ might rescue Ku depletions, by repeating the PCR assays shown in Figure 4C with more distant primers hybridizing 1 kb away from the IES excision site. This would have allowed us to detect alternative repair junctions with small deletions attributable to alt-NHEJ. Even under these conditions, however, no heterogeneous excision junctions could be detected (Figure S4). Second, because IES excision is concomitant with genome endoduplication, we reasoned that HR could use yet non-rearranged DNA molecules as templates to repair DSBs at IES excision sites: this would account for the amplification of IES^+^ molecules that we observed in the new MAC (Figure 4A). During HR-mediated DSB repair, the free 3′OH ends resulting from Pgm-dependent DNA cleavage would not be degraded during 5′ to 3′ resection, and should be detectable through polynucleotidyl terminal transferase (TdT) tailing [17]. In a control RNAi, indeed, free 3′OH ends were detected at IES boundaries (Figure 5B), with the same timing as the DSBs observed using LMPCR (Figure 5A). However, no free 3′OH ends were found at the expected position in a *KU80c* RNAi (Figure 5B). Taken together, our data do not support the hypothesis that, in cells depleted for Ku, broken IES excision sites are repaired through an alternative pathway involving 5′ to 3′ resection. Our results rather suggest that Ku is required for Pgm-dependent DNA cleavage itself.

### Ku and Pgm form a complex in soluble cell extracts

**Figure 7:**
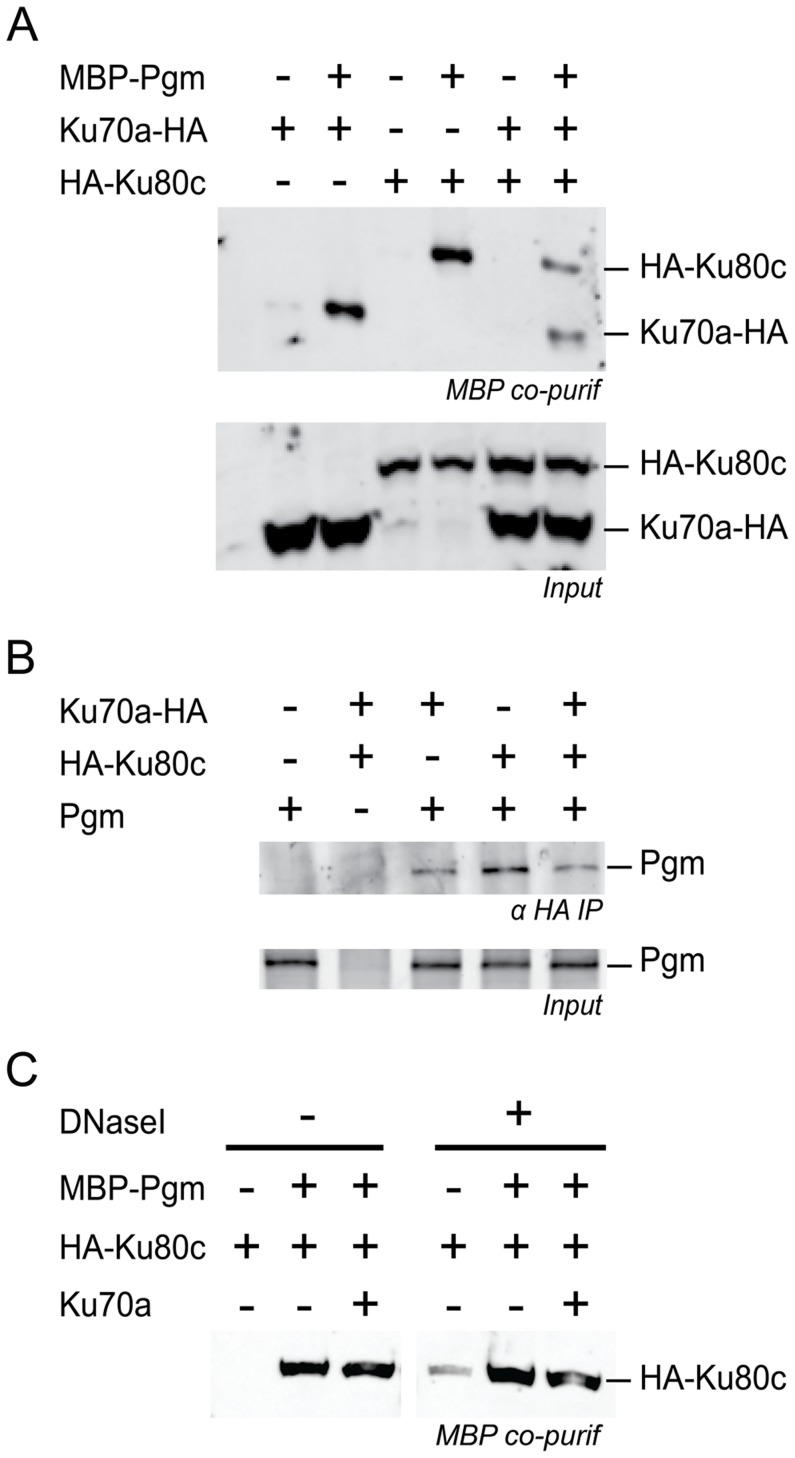
Physical interaction between Pgm and Ku. (A) Co-precipitation of HA-Ku80c and Ku70a-HA with MBP-Pgm from insect cell extracts (top panel), revealed on western blots using an anti-HA antibody. (B) Co-immunoprecipitation of Pgm with HA-Ku80c and/or Ku70a-HA from insect cell extracts, revealed on western blots using an anti-Pgm antibody. In A and B, the input proteins from each extract are displayed in the bottom panel. (C) The interaction between HA-Ku80c and Pgm is resistant to DNaseI treatment. The co-precipitation experiment was performed as described in A, using MBP-Pgm, HA-Ku80c and 6His-Ku70a recombinant proteins produced from baculovirus vectors. EDTA was removed from the lysis buffer and replaced by 10 mM MgCl_2_. Half of the sample was treated with 40 µg/mL of Dnase I during the 2-hr incubation with amylose-coupled magnetic beads. The presence of HA-Ku80c in the purified complexes was revealed on western blots using an anti-HA antibody.

A functional interaction between Ku and Pgm to activate DNA cleavage at IES boundaries may rely on the formation of a protein complex containing both proteins. To investigate this hypothesis, HA-tagged versions of Ku70a or Ku80c were produced in a heterologous insect cell system, together with Pgm fused to the maltose-binding protein (MBP) at its N-terminal end. For each condition, the MBP-Pgm protein was precipitated from soluble cell extracts using amylose magnetic beads, and co-precipitation of the Ku subunits was monitored on western blots (Figure 7A). We observed that Ku70a and Ku80c co-precipitated with MBP-Pgm, either individually or when the two subunits were co-expressed in the same cells. Control experiments confirmed that the enrichment in either Ku subunit is specific for the presence of MBP-Pgm in the extracts (Figure 7A). Reciprocally, we could co-immunoprecipitate Pgm with HA-tagged Ku from extracts of insect cells co-expressing both proteins, using magnetic beads coated with anti-HA antibodies (Figure 7B). The association of Pgm and Ku was resistant to DNase I (Figure 7C), suggesting that the formation of a Pgm/Ku complex does not depend upon the presence of DNA. Taken together, our data indicate that Pgm and Ku assemble in a higher-order complex in soluble cell extracts.

## Discussion

### A specific Ku heterodimer is required for MAC development as part of a developmental transcription program

Gene duplication has been proposed to be a driver of genome evolution, allowing the sub-functionalization of duplicated genes and sometimes leading to the emergence of novel cellular functions [25]. In *P. tetraurelia*, the presence of two *KU70* and three *KU80* genes has been the result of successive WGDs [18]. The *KU80* family provides a nice example of sub-functionalization, with *KU80a* and *KU80b* being constitutively expressed throughout the life cycle, and *KU80c* exhibiting a characteristic induction pattern correlating with its specific function during MAC development. Alignment of the three Ku80 proteins reveals that Ku80c differs from Ku80a and Ku80b at several positions (Figure S5), some of which may lie on the exposed surface of the α/β domain [26] and might possibly interact with additional partners. Future studies should address the question of whether the specific function of *KU80c* has resulted only from its overexpression or also involves a specialization of the protein in assisting PiggyMac-dependent genome rearrangements. The function(s) of the *KU70* genes could not be investigated separately through RNAi. The Ku70a and Ku70b proteins are 98% identical (see Figure S5), with only conservative amino acid changes in their α/β and β-barrel domains, which suggests that they perform similar functions. However, using RNA deep sequencing, we confirmed that the two recently duplicated *KU70* genes exhibit distinct transcription patterns, with *KU70a* being overexpressed during MAC development. We propose that, similar to *KU80c*, *KU70a* might have been undergoing specialization to carry out an essential function in PGR.

According to previous microarray hybridization analyses, successive transcription induction peaks (early, intermediate and late) were identified at the genome-wide level during autogamy [20]. *KU80c* and *PGM* belong to the same “intermediate” cluster of genes that are induced by the time PGR take place during MAC development, and are repressed at later time-points during autogamy. Our study shows that the transcription of *PGM* is induced normally after Ku80c depletion. However, while *PGM* expression decreases at late time-points in a control, after PGR are essentially completed, it is continuously turned on in cells depleted in Ku80c, while PGR are strongly inhibited. Reciprocally, after Pgm depletion, which blocks PGR, the transcription of *KU80c* is induced normally but is not switched off. Quite interestingly, normal progression of autogamy is observed at the cytological level upon Ku80c or Pgm depletion, indicating that cytological progression of MAC development (DNA amplification, segregation of the new developing MACs into daughter cells) in *Paramecium* is uncoupled from the completion of PGR. Although alternative explanations might be proposed, the observation that similar transcriptional deregulation is observed at late autogamy time-points in *KU80c* and *PGM* knockdowns suggests that the cause of aberrant transcript accumulation for genes from the intermediate cluster is a failure to complete PGR. For instance, a transcriptional activator specific for the intermediate gene cluster may be expressed from an IES-containing gene in the developing MAC, and switched off as soon as IES excision has been completed. A regulatory mechanism relying on the retention of an IES overlapping a gene promoter has been shown to control the expression of a mating-type gene in *P. tetraurelia* [27]. In the ciliate *Euplotes crassus*, PGR also regulate the expression of a development-specific telomerase gene that is localized in the germline-restricted part of the genome and is, therefore, switched off naturally once it is eliminated [28]. The existence of feedback regulatory loops provides a nice illustration of how ciliates may take advantage of PGR to fine-tune gene expression during their sexual cycle.

### The unexpected role of Ku in *Paramecium*: Coupling of DNA cleavage and DSB repair during PGR

Upon Ku70 or Ku80c depletions, we found that germline sequences are retained in the new MAC: both IES excision and chromosome fragmentation are inhibited, which confirms that the developmental-specific Ku70/Ku80c heterodimer plays an essential role in PGR. We confirmed that *PGM* expression, which is required for the two types of genome rearrangements [15], is still induced in Ku-depleted cells and that Pgm still localizes to the developing new MACs. As discussed above, the persisting overproduction of Pgm at late autogamy time-points appears to be a consequence, and not the cause, of defective genome rearrangements in Ku-depleted cells.

With regard to IES excision, no *de novo* precise excision junctions were detected upon Ku depletion, consistent with a defective C-NHEJ pathway. Neither did we detect any imprecise junction that may have resulted from alt-NHEJ. More surprisingly, we observed that the non-rearranged version of the genome is amplified in the new MAC, and that no DSBs with the expected geometry can be detected at IES boundaries using a sensitive LMPCR molecular approach. Because IES excision starts after 3 to 4 rounds of genome amplification in the new MAC [29], we considered the possibility that HR substitutes for end-joining during the repair of IES excision sites in Ku-depleted cells, which could restore IES^+^ chromosomes, supposing that a yet non-rearranged sister chromatid were used as a template. During HR-mediated repair, DSBs would be processed through 5′ to 3′ resection, which would make broken ends inappropriate substrates for LMPCR. However, using a sensitive TdT-tailing assay, we obtained no evidence that HR intermediates are formed, based on the absence of detectable free 3′ ends at IES boundaries. We therefore conclude that IESs are retained in the genome of Ku-depleted cells, as a consequence of defective programmed DNA cleavage at their boundaries. Our findings point to the participation of the development-specific Ku70-Ku80c heterodimer in Pgm-dependent DNA cleavage, upstream of its likely function in C-NHEJ-mediated DSB repair.

In another ciliate, *Tetrahymena thermophila*, IES excision is mediated by a Pgm homolog, Tpb2p, which is responsible for DNA cleavage at IES boundaries [30], [31]. *T. thermophila* harbors one *KU80* and two *KU70* genes: Tpb2p-dependent DNA cleavage does occur in a *TKU80*Δ strain, but the resulting DSBs are not repaired, leading to an arrest in MAC development and to DNA loss in the new MAC [32]. These phenotypes, which are quite similar to those described for Ligase IV or Xrcc4 depletions in *P. tetraurelia* [16], are fully consistent with a classical scenario, in which C-NHEJ factors are recruited to broken DNA ends after DNA cleavage at IES boundaries. Interestingly, the presence of Ku is not a prerequisite for DNA cleavage in *T. thermophila*, and Tku80p localization remains dispersed throughout the developing MAC when Tpb2p concentrates in large heterochromatin bodies, in which DNA elimination is thought to take place [32]. It is unclear, therefore, whether Ku interacts directly with Tpb2p during genome rearrangements in this ciliate. Together with the presence of a single *KU80* gene in *T. thermophila*, the observed differences between the two ciliates suggest that specialization of *KU80c* in *P. tetraurelia* may have occurred after the divergence between *Paramecium* and *Tetrahymena*. Noteworthy, IES excision is rather imprecise in *T. thermophila*, and heterogeneity at IES excision junctions could be attributed to variability in the choice of Tpb2p cleavage sites [33] or to the participation of different mechanisms in the formation of IES excision junctions, such as C-NHEJ-mediated DSB repair [32] or trans-esterification if a single boundary is cleaved [34], [35]. Accordingly, *T. thermophila* appears to have avoided IES insertion into coding regions [36], where imprecise excision could be deleterious.

**Figure 8:**
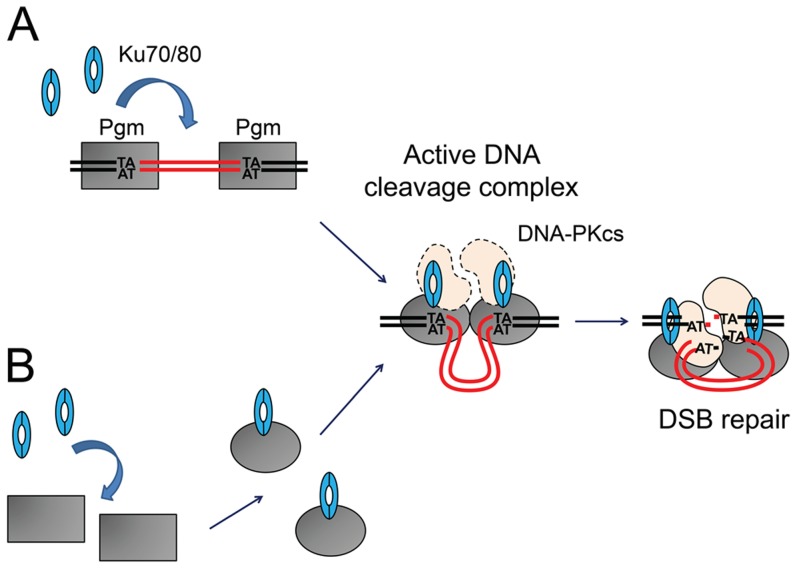
Models for the assembly of an active DNA cleavage complex. (A) Ku associates with DNA-bound Pgm and activates DNA cleavage. Pgm and its putative partners (in grey) would recognize and bind the boundaries of eliminated sequences. The binding of Ku (in blue) activates Pgm for DNA cleavage (symbolized by the switch from a rectangular box to an oval), perhaps by assisting the formation of a synapse between both IES ends, in a transpososome-like intermediate. The DNA-PKcs catalytic subunit (in peach) may also be part of the active DNA cleavage complex. (B) Ku forms a complex with Pgm in the absence of DNA, activating the DNA binding and/or cleavage activities of Pgm. Following DNA cleavage, conformational remodeling of the complex would position Ku on broken DNA ends and allow it to perform its classical role in C-NHEJ-mediated DSB repair. MAC DNA is represented in black, IES DNA in red.

The situation is quite different in *P. tetraurelia*, in which 47% of genes are interrupted by at least one IES [11]. The pressure to assemble functional open reading frames in the somatic genome has driven the emergence of a highly efficient and precise IES excision mechanism. Several known features of the excision process contribute to this precision: (i) the establishment of a crosstalk between IES ends before DNA cleavage, within a transpososome-like complex [11], [24], and (ii) the precise positioning of Pgm-dependent cleavages on each flanking TA [17]. Here, we show that the development-specific Ku70/Ku80c heterodimer is required to activate Pgm-dependent DNA cleavage at IES boundaries *in vivo*. Although we excluded an inhibitory effect of Ku80c depletion on *PGM* transcription, we cannot formally rule out that a Ku-dependent upstream developmental event activates DNA cleavage. However, our observation that Ku70a and Ku80c interact with Pgm in pull-down experiments suggests that DNA cleavage and C-NHEJ-mediated DSB repair are tightly intertwined through the incorporation of the development-specific Ku70a/Ku80c heterodimer into the DNA cleavage complex itself (Figure 8): programmed DSBs, therefore, would be efficiently directed towards C-NHEJ-mediated precise repair. We propose that the Ku70/Ku80c heterodimer is an essential Pgm partner that activates the assembly of a transpososome-like nucleoprotein complex competent for DNA cleavage. In support to this model, we found that Ku70a and Ku80c interact with Pgm in cell extracts. During IES excision, Ku and Pgm may associate once Pgm is bound to IES boundaries (Figure 8A: activation of Pgm-dependent cleavage), or before Pgm interacts with DNA, as suggested by our observation that the Pgm/Ku complex is resistant to DNase I *in vitro* (Figure 8B: activation of the DNA binding activity of Pgm). Further biochemical work is needed to analyze the Ku/Pgm interaction and clarify the mechanisms involved in Ku-mediated activation of DNA cleavage. We cannot exclude at this stage that the DNA-PKcs is also a component of the DNA cleavage complex (Figure 8): indeed, a parallel study established that DNA-PKcs depletion impairs the excision of at least a subset of IESs, which exhibit a reduced efficiency of DNA cleavage at their boundaries (Malinsky et al., in prep.). The incorporation of early C-NHEJ proteins in the cleavage complex would allow the cell to face the challenge of repairing efficiently and precisely tens of thousands of DSBs during the short period of MAC development. In contrast, Ligase IV and Xrcc4 are clearly not required for DNA cleavage [16] and are probably not part of the cleavage complex.

### Integration of DNA cleavage and repair in recombination factories during PGR

Domesticated transposases from cut-and-paste DNA transposons might have been recruited to perform PGR in various systems, not only because of their DNA cleavage activities, but perhaps also because of the particular features of cut-and-paste transposition [37]. When transposons integrate into their target site, they duplicate a short sequence, the TSD (target site duplication), on each side of the integrated element. During the next round of transposition, the transposase cuts the DNA, excises the transposon and leaves a DSB at the donor site, which is repaired through cellular pathways. The study of cut-and-paste transposons has revealed that C-NHEJ, as opposed to alt-NHEJ, accounts for most end-joining events during DSB repair at transposon donor sites (reviewed in [5]). Moreover, several cut-and-paste transposons and/or their transposases interact with Ku. For instance, Ku70 binds the ends of the *P* element from *Drosophila melanogaster* and stimulates DSB repair at the donor sites [38]. *In vitro*, the transposase of *Sleeping Beauty*, a reconstructed *Tc/mariner* transposon, forms a complex with Ku70, and efficient transposition of *Sleeping Beauty* in a cellular system depends on the presence of DSB repair proteins [39]. However, it is not clear *in vivo* whether Ku/transposase interaction activates DNA cleavage at transposon ends or simply facilitates the recruitment of the C-NHEJ pathway once the donor site has been broken.

With regard to PGR, the formation of a complex involving a nuclease and DSB repair factors has been hypothesized during V(D)J recombination [40]. *In vitro*, Ku interacts with the domesticated transposase RAG1 [41], but no evidence has been provided that Ku is required *in vivo* for the introduction of RAG1-dependent programmed DSBs. As in *Tetrahymena*, the formation of nuclear recombination centers, or recombination factories, may facilitate V(D)J recombination by bringing together the recombination sites, the RAG1/RAG2 endonuclease and DSB repair factors. However, in this system, the presence of C-NHEJ proteins appears dispensable for DNA cleavage itself. The interplay between DNA cleavage and DSB repair has been pushed one step further in *Paramecium*. Indeed, the present study of IES excision in *P. tetraurelia* provides the first evidence that Ku is absolutely required *in vivo* to introduce programmed DSBs during PGR. The demonstration that DNA cleavage mediated by a transposase-related protein and C-NHEJ mediated repair are coupled during PGR in *P. tetraurelia* is reminiscent of the observation that, during meiosis in *S. cerevisiae*, the Mre11p HR protein is required for DNA cleavage by the topoisomerase-like Spo11 endonuclease [42]. These two systems support the notion that the presence of DSB repair factors in recombination factories may be a prerequisite for DNA cleavage during programmed genome rearrangements.

## Materials and Methods

### *P. tetraurelia* strains and growth conditions

For autogamy time-course experiments, we used *P. tetraurelia* strain 51 new (hereafter called 51) and its 51ΔA variant carrying a heritable deletion of the *A* gene in its MAC but harboring a wild-type MIC [24]. To facilitate the screening of transformants in microinjection experiments, we introduced the *nd7-1* mutation [43] into strain 51 by conjugation, or used somatic variants carrying a MAC deletion of the *ND7* gene [44]. Cells were grown at 27°C in a wheat grass infusion (WGP; Pines International Inc.) inoculated with *Klebsiella pneumoniae*. Autogamy was carried out through starvation as described [29]. Total RNA and genomic DNA were extracted from ∼400,000 cells for each time-point and quantified as described [15].

### Molecular procedures

Oligonucleotides were purchased from Sigma-Aldrich or Eurofins MWG Operon (Table S1).

PCR amplifications were performed in a final volume of 25 µL, with 10 pmol of each primer, 5 nmol of each dNTP and 1 U of DyNAzyme II DNA polymerase (Finnzymes) or DreamTaq (Thermo Scientific) according to the enzyme suppliers' recommendations. PCR products were analyzed on 3% NuSieve GTG agarose gels (BioWhittaker Molecular Applications). LMPCR detection of double-strand breaks was performed as described [24]. Poly(C) tailing of free 3′ ends using terminal transferase was performed as described [17], using 500 ng of input total genomic DNA. Sanger DNA sequencing was performed at GATC Biotech, or using the fmol DNA Cycle Sequencing System (Promega). Northern and Southern blot hybridization with ^32^P-labeled probes was carried out as described [15]. The *KU70* and *KU80c* probes are described in Figure S2 and Table S1. The sequence of the 17S rRNA oligonucleotide probe is shown in Table S1.

### Protein depletion by RNAi

#### Description of RNAi plasmids

All RNAi plasmids are derivatives of vector L4440 [45] and carry a target gene fragment between two convergent T7 promoters. Each insert was chosen in order to minimize the risk of cross-silencing by using the “RNAi-off-target” tool of ParameciumDB [46]. Plasmids pXRCC4-R [16], pPGM-1 [15], p0ND7c [44] and pICL7a [47] carry RNAi inserts targeting the *XRCC4*, *PGM*, *ND7* and *ICL7a* genes, respectively. RNAi plasmids targeting the *KU* genes were constructed as follows:

pL4440-KU70a-1: a 300-bp fragment from gene *KU70a* (bp 514–813 from ATG start codon) was amplified by PCR using primers ku70A-BamHI_1 and ku70A-KpnI_1, digested with *Bam*HI and *Kpn*I, and ligated between the *Bgl*II and *Kpn*I sites of plasmid L4440.

pL4440-KU80a-1: a 233-bp fragment from gene *KU80a* (bp 40–272 from ATG start codon) was amplified by PCR using primers ku80A-BamHI_1 and ku80A-KpnI_1, digested with *Bam*HI and *Kpn*I, and ligated between the *Bgl*II and *Kpn*I sites of plasmid L4440.

pL4440-KU80a-2: a 450-bp fragment from gene *KU80a* (bp 580–1029 bp from ATG start codon) was amplified by PCR using primers OMB223 and OMB224, digested with *Spe*I and ligated into the *Xba*I site of plasmid L4440.

pL4440-KU80b-1: a 233-bp fragment from gene *KU80b* (bp 40–272 from ATG start codon) was amplified by PCR using primers OMB219 and OMB220, digested with *Spe*I and ligated into the *Xba*I site of plasmid L4440.

pL4440-KU80b-2: a 450-bp fragment from gene *KU80b* (bp 580–1029 bp from ATG start codon) was amplified by PCR using primers OMB221 and OMB222, digested with *Spe*I and ligated into the *Xba*I site of plasmid L4440.

pL4440-KU80c-1: a 206-bp fragment from gene *KU80c* (bp 1021–1226 from ATG start codon) was amplified by PCR using primers ku80C-BamHI_1 and ku80C-KpnI_1bis, digested with *Bam*HI and *Kpn*I, and ligated between the *Bgl*II and *Kpn*I sites of plasmid L4440.

pL4440-KU80c-2: a fragment from gene *KU80C* was amplified by PCR using primers OMB225 and ku80C-KpnI_1bis. Following restriction with *Spe*I, a 450-bp sub-fragment (bp 557–1006 from ATG start codon) was inserted into the *Xba*I site of plasmid L4440.

#### Outline of the “feeding” procedure

RNA interference was achieved as described [15], by feeding *P. tetraurelia* strains 51 or 51ΔA on *Escherichia coli* HT115 bacteria transformed with each plasmid and induced for the production of double-strand RNA corresponding to each RNAi insert. Survival of the progeny was tested at day 4 of starvation by transferring 30 individual autogamous cells to standard *K. pneumoniae* medium.

### Determination of gene expression levels by RNA-seq

Strand-specific RNA-seq libraries were prepared from 50 ng polyA+ RNA following the directional mRNA-seq library preparation protocol provided by Illumina: RNAs were fragmented using fragmentation buffer, purified and treated with phosphatase and kinase prior to sequential ligation with different RNA adapters to the 3′ and 5′ ends. The ligated RNA fragments were reverse-transcribed, followed by PCR amplification. Each library was sequenced using an Illumina Genome Analyzer IIx to generate 75-nt paired-end reads. Reads were mapped with TopHat2 [48] (read-mismatches 1; min-intron-length 15; max-intron-length 100) on the *P. tetraurelia* strain 51 reference MAC genome [11]. Alignments were indexed using Samtools [49] and a custom perl script was used to count uniquely mapped fragments for each gene model. The counts were normalized to account for gene size and the total number of mapped fragments.

### Construction of GFP fusions

For the construction of in-frame *GFP-KU* fusions, a GFP-coding fragment adapted to *Paramecium* codon usage [50] was added by PCR fusion to the 5′ end of the *KU70a* or *KU80c* genes. Each construct was inserted in a pUC18 plasmid between the *SphI* and *SacI* sites. As a result, the GFP is fused to the N-terminus of Ku70a and Ku80c and the fusion proteins are expressed under the control of the *KU70a* and *KU80c* transcription signals (promoters and 3′UTR), respectively. The *PGM-GFP* fusion will be described in detail elsewhere (Dubois et al., in prep.). Briefly, the GFP-coding fragment was added by PCR fusion to the 3′ end of the *PGM* gene carried by plasmid pPBL49g [15]: in the resulting construct, the *PGM-GFP* coding sequence is flanked by 96 bp upstream of the ATG (i.e. the putative endogenous *PGM* promoter) and 54 bp downstream of the TGA stop codon (including the endogenous 3′ UTR and the polyadenylation site).

### Transformation of *Paramecium* cells and localization of fluorescent fusion proteins

Plasmids encoding GFP fusion proteins were linearized by appropriate restriction enzymes and microinjected into the MAC of vegetative 51 *nd7-1* or 51ΔND7 cells, as described [15]. A complementing plasmid carrying a functional *ND7* gene [43] was coinjected with the fusion transgenes to facilitate the selection of transformants. During autogamy, cells were permeabilized for 4 min in PHEM (60 mM Pipes, 25 mM Hepes, 10 mM EGTA, 2 mM MgCl_2_ pH 6.9) +1% Triton, then fixed for 10 min in PHEM +2% paraformaldehyde. All observations were performed using a Zeiss Axioplan 2 Imaging epifluorescence microscope. Developing MACs were identified using Nomarski differential interference contrast (DIC) combined with 4′,6-diamidino-2-phenylindole (DAPI) staining. No lethality was observed in the post-autogamous progeny of transformed cells.

### Protein expression in insect cells and co-precipitation assays

#### Plasmids and vectors

DNA sequences coding for Pgm, Ku70a and Ku80c were obtained by gene synthesis (DNA 2.0 or Eurofins MWG/Operon). The synthetic *PGM* DNA sequence was first cloned into the pMAL-c2x vector (New England Biolabs). The *MBP-PGM* fusion, the *MBP*, the *KU70a* and the *KU80c* sequences were further introduced into the pVL-1392 vector (BD Biosciences). A HA tag was added to the C-terminus of Ku70a and the N-terminus of Ku80c. All plasmid sequences are provided in a supplementary information file (Text S1). Plasmids pVL1392-MBP-PGM, pVL1392-MBP, pVL1392-KU70a-HA and pVL1392-HA-KU80c were transfected individually into High Five cells together with the BD BaculoGold Linearized Baculovirus DNA (BD Biosciences).

#### Preparation of soluble protein extracts from baculovirus-infected High Five cells

High Five cells were grown in the EX-CELL 405 synthetic medium (Sigma Aldrich) supplemented with Penicillin and Streptomycin. For co-expression experiments, 10^6^ cells are infected with *MBP*, *MBP-PGM*, *KU70a-HA* and/or *HA-KU80c* recombinant baculoviruses. In this system, each gene is expressed under the control of a late viral gene. At 48 hrs post-infection, cells were collected, washed with cold PBS buffer and re-suspended in 500 µl of lysis buffer containing 10 mM Tris-HCL pH 7.5, 150 mM NaCl, 1% Triton X-100(v/v), 1 mM EDTA, 1 mM DTT and 0.5% NaDesoxycholate (m/v) in the presence of protease inhibitors (Complete Protease Inhibitor Cocktail Tablets, Roche). Cells were lysed for 20 min on a rotating wheel, then centrifuged at 4°C for 15 min at 10,000 g.

#### Co-precipitation assays

For MBP-tagged proteins, soluble extracts were incubated for 2 hrs with 100 µg of amylose magnetic beads (New England Biolabs). For HA-tagged proteins, 1.5 µg of monoclonal anti-HA antibodies (HA-7 from Sigma Aldrich) were incubated overnight on a rotating wheel at 4°C with 10 µl of protein G magnetic beads (New England Biolabs), then cell extracts were incubated for 2 hrs with the coated beads. In both experiments, the beads were washed 3 times with 1 ml of lysis buffer, then re-suspended in Laemmli buffer before electrophoresis in SDS-polyacrylamide gels. Aliquots were saved for input control. HA-tagged Ku proteins were detected on western blots, using monoclonal anti-HA primary antibodies (same as above) and anti-mouse HRP-coupled secondary antibodies (Promega). MBP-tagged proteins were detected with anti-MBP HRP-coupled antibodies (New England Biolabs). Pgm was detected using polyclonal anti-Pgm rabbit primary antibodies (Proteogenics) raised against the DKNEKDAEDEFQDLNPSEHK peptide from the N-terminus of Pgm, and an anti-rabbit HRP-coupled secondary antibody (Promega).

## Supporting Information

Figure S1 Progression of autogamy in the cultures used in this study. For each time-point, cell stages were monitored by DAPI staining. Veg: vegetative cells. Skeins: cells with elongating old MAC at the beginning of fragmentation. Meiosis*: cells with detectable meiotic MICs (which is an underestimate of the actual fraction of meiotic cells). Fragments: cells with fully fragmented old MAC, but no visible new MAC (too small or indistinguishable from fragments). 2 anlagen: cells with two visible new developing MACs. postA: post autogamous cells with one new MAC and a few fragments of the old MAC. (A) Autogamy time course of strain 51 subjected to RNAi against *ND7* or *PGM*. The ND7 and PGM-1 constructs used for RNAi were described in [15]. (B) Autogamy time course of strain 51 subjected to RNAi against *ND7* or *KU80c* (using the KU80c-2 construct, see Figure S2). (C) Autogamy time course of strain 51ΔA subjected to RNAi against *ICL7a* (RNAi construct described in [47]) or *KU80c* (RNAi construct: KU80c-2). Note that in the *ICL7* RNAi, a fraction of cells underwent autogamy prematurely, accounting for the presence of 20% of cells with fragmented MACs in time-point 1 (VIcl7) and for the detection of 10% post-autogamous cells (postA) in the T0 sample. Taking into account the asynchrony of the *ICL7* RNAi experiment, the corresponding stages between the ICL7 and KU80c RNAi experiments were numbered from 1 to 6 (intermediate stages were labeled with a′).
(TIF)

Figure S2 Maps of the *KU70* and *KU80* genes of *P. tetraurelia*. The maps show the position of the inserts used for RNAi constructs (blue) and hybridization probes (red, see Table S1). For *KU70* genes, all fragments were designed from the *KU70a* sequence. Specific fragments were designed for each *KU80* gene: all experiments described in the paper were performed using inserts KU80-a2, KU80-b2 and KU80-c2. Qualitatively similar phenotypes (survival/lethality in sexual progeny and presence/absence of *de novo* IES excision junctions) were observed when RNAi was performed using inserts KU80-a1 (which also targets *KU80b* mRNA) and KU80-c1.
(TIF)

Figure S3 Detection of *de novo* IES excision junctions in different RNAi conditions. The excision of IESs 51A1835, 51A2591 and 51A4404 during autogamy of strain 51ΔA (4 days of starvation) was tested by PCR amplification (PCR primers are displayed in Table S1). RNAi conditions are indicated next to each panel. Here, starvation was prolonged for one additional day relative to the other experiments shown in the paper. Therefore, day 4 corresponds to approximately 20 hours following time-point 6 in Figure 4. The low levels of IES^−^ forms that are detected very late during autogamy might be attributable to the recovery of significant amounts of full-length *KU80c* mRNA upon prolonged starvation (see Figure 3C and related text).
(TIF)

Figure S4 Search for alternative excision junctions for IES 51A2591. (A) Map of IES 51A2591 and its flanking MAC-destined sequences. The primers used for PCR reactions are indicated by arrowheads (see Table S1). (B) No detection of alternative *de novo* IES excision junctions during autogamy of 51ΔA cells, in an *ICL7* (control) or a *KU80c* RNAi. PCR reactions were performed using primers OMB808 and OMB809 and loaded on a 2% agarose gel.
(TIF)

Figure S5 Alignments of the Ku70 and Ku80 homologs from *P. tetraurelia.* All sequences were retrieved from the ParameciumDB database, using the following accession numbers: GSPATP00006445001 (Ku70a), GSPATP00009747001 (Ku70b), GSPATP00034664001 (Ku80a), GSPATP00035446001 (Ku80b), GSPATP00030095001 (Ku80c). Alignments of Ku80 (panel A) and Ku70 homologs (panel B) were performed separately using the MUSCLE multiple sequence alignment software (http://www.ebi.ac.uk/Tools/msa/muscle/) and colored using the BoxShade server (http://www.ch.embnet.org/software/BOX_form.html). For each Ku subunit, domain annotation was based on Pfam analysis of conserved domains (http://pfam.xfam.org/ and [52]).
(PDF)

Table S1 Oligonucleotides used in this study.
(PDF)

Text S1 Plasmid sequences. This file displays the sequence of all pVL-1392 derivatives used for the construction of baculovirus vectors designed for protein expression in insect cells: pVL1392-MBP-PGM (MBP-Pgm), pVL1392-MBP (MBP tag alone), pVL1392-Ku70a-HA (Ku70a-HA) and pVL1392-HA-Ku80c (HA-Ku80c).
(DOCX)

## References

[1] Chapman JR, Taylor MR, Boulton SJ (2012) Playing the end game: DNA double-strand break repair pathway choice. Mol Cell 47: 497–510. 22920291 10.1016/j.molcel.2012.07.029

[2] Longhese MP, Bonetti D, Guerini I, Manfrini N, Clerici M (2009) DNA double-strand breaks in meiosis: checking their formation, processing and repair. DNA Repair (Amst) 8: 1127–1138. 19464965 10.1016/j.dnarep.2009.04.005

[3] Schatz DG, Swanson PC (2011) V(D)J recombination: mechanisms of initiation. Annu Rev Genet 45: 167–202. 21854230 10.1146/annurev-genet-110410-132552

[4] Lieber MR (2010) The mechanism of double-strand DNA break repair by the nonhomologous DNA end-joining pathway. Annu Rev Biochem 79: 181–211. 20192759 10.1146/annurev.biochem.052308.093131 PMC3079308

[5] Bétermier M, Bertrand P, Lopez BS (2014) Is non-homologous end-joining really an inherently error-prone process? PLoS Genet 10: e1004086. 24453986 10.1371/journal.pgen.1004086 PMC3894167

[6] McVey M, Lee SE (2008) MMEJ repair of double-strand breaks (director's cut): deleted sequences and alternative endings. Trends Genet 24: 529–538. 18809224 10.1016/j.tig.2008.08.007 PMC5303623

[7] Truong LN, Li Y, Shi LZ, Hwang PY, He J, et al (2013) Microhomology-mediated End Joining and Homologous Recombination share the initial end resection step to repair DNA double-strand breaks in mammalian cells. Proc Natl Acad Sci U S A 110: 7720–7725. 23610439 10.1073/pnas.1213431110 PMC3651503

[8] Symington LS, Gautier J (2011) Double-strand break end resection and repair pathway choice. Annu Rev Genet 45: 247–271. 21910633 10.1146/annurev-genet-110410-132435

[9] Chalker DL, Yao MC (2011) DNA elimination in ciliates: transposon domestication and genome surveillance. Annu Rev Genet 45: 227–246. 21910632 10.1146/annurev-genet-110410-132432

[10] Dubois E, Bischerour J, Marmignon A, Mathy N, Régnier V, et al (2012) Transposon Invasion of the *Paramecium* Germline Genome Countered by a Domesticated PiggyBac Transposase and the NHEJ Pathway. Int J Evol Biol 2012: 436196. 22888464 10.1155/2012/436196 PMC3408717

[11] Arnaiz O, Mathy N, Baudry C, Malinsky S, Aury JM, et al (2012) The *Paramecium* germline genome provides a niche for intragenic parasitic DNA: Evolutionary dynamics of internal eliminated sequences. PloS Genetics 8: e1002984. 23071448 10.1371/journal.pgen.1002984 PMC3464196

[12] Coyne RS, Lhuillier-Akakpo M, Duharcourt S (2012) RNA-guided DNA rearrangements in ciliates: is the best genome defence a good offence? Biol Cell 104: 309–325. 22352444 10.1111/boc.201100057

[13] Lepère G, Bétermier M, Meyer E, Duharcourt S (2008) Maternal noncoding transcripts antagonize the targeting of DNA elimination by scanRNAs in *Paramecium tetraurelia* . Genes Dev 22: 1501–1512. 18519642 10.1101/gad.473008 PMC2418586

[14] Lepère G, Nowacki M, Serrano V, Gout JF, Guglielmi G, et al (2009) Silencing-associated and meiosis-specific small RNA pathways in *Paramecium tetraurelia* . Nucleic Acids Res 37: 903–915. 19103667 10.1093/nar/gkn1018 PMC2647294

[15] Baudry C, Malinsky S, Restituito M, Kapusta A, Rosa S, et al (2009) PiggyMac, a domesticated *piggyBac* transposase involved in programmed genome rearrangements in the ciliate *Paramecium tetraurelia* . Genes Dev 23: 2478–2483. 19884254 10.1101/gad.547309 PMC2779751

[16] Kapusta A, Matsuda A, Marmignon A, Ku M, Silve A, et al (2011) Highly precise and developmentally programmed genome assembly in *Paramecium* requires Ligase IV-dependent end joining. PloS Genetics 7: e1002049. 21533177 10.1371/journal.pgen.1002049 PMC3077386

[17] Gratias A, Bétermier M (2003) Processing of double-strand breaks is involved in the precise excision of *Paramecium* IESs. Mol Cell Biol 23: 7152–7162. 14517286 10.1128/MCB.23.20.7152-7162.2003 PMC230320

[18] Aury JM, Jaillon O, Duret L, Noel B, Jubin C, et al (2006) Global trends of whole-genome duplications revealed by the ciliate *Paramecium tetraurelia* . Nature 444: 171–178. 17086204 10.1038/nature05230

[19] Bétermier M (2004) Large-scale genome remodelling by the developmentally programmed elimination of germ line sequences in the ciliate *Paramecium* . Res Microbiol 155: 399–408. 15207872 10.1016/j.resmic.2004.01.017

[20] Arnaiz O, Gout JF, Bétermier M, Bouhouche K, Cohen J, et al (2010) Gene expression in a paleopolyploid: a transcriptome resource for the ciliate *Paramecium tetraurelia* . BMC Genomics 11: 547. 20932287 10.1186/1471-2164-11-547 PMC3091696

[21] Galvani A, Sperling L (2002) RNA interference by feeding in *Paramecium* . Trends Genet 18: 11–12. 11750689 10.1016/s0168-9525(01)02548-3

[22] Frank KM, Sekiguchi JM, Seidl KJ, Swat W, Rathbun GA, et al (1998) Late embryonic lethality and impaired V(D)J recombination in mice lacking DNA ligase IV. Nature 396: 173–177. 9823897 10.1038/24172

[23] Duharcourt S, Butler A, Meyer E (1995) Epigenetic self-regulation of developmental excision of an internal eliminated sequence in *Paramecium tetraurelia* . Genes Dev 9: 2065–2077. 7649484 10.1101/gad.9.16.2065

[24] Gratias A, Lepère G, Garnier O, Rosa S, Duharcourt S, et al (2008) Developmentally programmed DNA splicing in *Paramecium* reveals short-distance crosstalk between DNA cleavage sites. Nucleic Acids Res 36: 3244–3251. 18420657 10.1093/nar/gkn154 PMC2425466

[25] Taylor JS, Raes J (2004) Duplication and divergence: the evolution of new genes and old ideas. Annu Rev Genet 38: 615–643. 15568988 10.1146/annurev.genet.38.072902.092831

[26] Walker JR, Corpina RA, Goldberg J (2001) Structure of the Ku heterodimer bound to DNA and its implications for double-strand break repair. Nature 412: 607–614. 11493912 10.1038/35088000

[27] Singh DP, Saudemont B, Guglielmi G, Arnaiz O, Gout JF, et al (2014) Genome-defence small RNAs exapted for epigenetic mating-type inheritance. Nature 509: 447–452. 24805235 10.1038/nature13318

[28] Karamysheva Z, Wang L, Shrode T, Bednenko J, Hurley LA, et al (2003) Developmentally programmed gene elimination in *Euplotes crassus* facilitates a switch in the telomerase catalytic subunit. Cell 113: 565–576. 12787498 10.1016/s0092-8674(03)00363-5

[29] Bétermier M, Duharcourt S, Seitz H, Meyer E (2000) Timing of developmentally programmed excision and circularization of *Paramecium* internal eliminated sequences. Mol Cell Biol 20: 1553–1561. 10669733 10.1128/mcb.20.5.1553-1561.2000 PMC85339

[30] Cheng CY, Vogt A, Mochizuki K, Yao MC (2010) A domesticated piggyBac transposase plays key roles in heterochromatin dynamics and DNA cleavage during programmed DNA deletion in *Tetrahymena thermophila* . Mol Biol Cell 21: 1753–1762. 20357003 10.1091/mbc.E09-12-1079 PMC2869380

[31] Vogt A, Mochizuki K (2013) A Domesticated PiggyBac Transposase Interacts with Heterochromatin and Catalyzes Reproducible DNA Elimination in *Tetrahymena* . PLoS Genet 9: e1004032. 24348275 10.1371/journal.pgen.1004032 PMC3861120

[32] Lin IT, Chao JL, Yao MC (2012) An essential role for the DNA breakage-repair protein Ku80 in programmed DNA rearrangements in *Tetrahymena* thermophila. Mol Biol Cell 23: 2213–2225. 22513090 10.1091/mbc.E11-11-0952 PMC3364183

[33] Saveliev SV, Cox MM (1995) Transient DNA breaks associated with programmed genomic deletion events in conjugating cells of *Tetrahymena thermophila* . Genes Dev 9: 248–255. 7851797 10.1101/gad.9.2.248

[34] Saveliev SV, Cox MM (1996) Developmentally programmed DNA deletion in *Tetrahymena thermophila* by a transposition-like reaction pathway. EMBO J 15: 2858–2869. 8654384 PMC450224

[35] Saveliev SV, Cox MM (2001) Product analysis illuminates the final steps of IES deletion in *Tetrahymena thermophila* . EMBO J 20: 3251–3261. 11406601 10.1093/emboj/20.12.3251 PMC150193

[36] Fass JN, Joshi NA, Couvillion MT, Bowen J, Gorovsky MA, et al (2011) Genome-Scale Analysis of Programmed DNA Elimination Sites in Tetrahymena thermophila. G3 (Bethesda) 1: 515–522. 22384362 10.1534/g3.111.000927 PMC3276166

[37] Feschotte C, Pritham EJ (2007) DNA transposons and the evolution of eukaryotic genomes. Annu Rev Genet 41: 331–368. 18076328 10.1146/annurev.genet.40.110405.090448 PMC2167627

[38] Beall EL, Rio DC (1996) *Drosophila* IRBP/Ku p70 corresponds to the mutagen-sensitive mus309 gene and is involved in P-element excision *in vivo* . Genes Dev 10: 921–933. 8608940 10.1101/gad.10.8.921

[39] Izsvak Z, Stuwe EE, Fiedler D, Katzer A, Jeggo PA, et al (2004) Healing the wounds inflicted by sleeping beauty transposition by double-strand break repair in mammalian somatic cells. Mol Cell 13: 279–290. 14759372 10.1016/s1097-2765(03)00524-0

[40] Schatz DG, Ji Y (2011) Recombination centres and the orchestration of V(D)J recombination. Nat Rev Immunol 11: 251–263. 21394103 10.1038/nri2941

[41] Raval P, Kriatchko AN, Kumar S, Swanson PC (2008) Evidence for Ku70/Ku80 association with full-length RAG1. Nucleic Acids Res 36: 2060–2072. 18281312 10.1093/nar/gkn049 PMC2330247

[42] Borde V (2007) The multiple roles of the Mre11 complex for meiotic recombination. Chromosome Res 15: 551–563. 17674145 10.1007/s10577-007-1147-9

[43] Skouri F, Cohen J (1997) Genetic approach to regulated exocytosis using functional complementation in *Paramecium*: identification of the *ND7* gene required for membrane fusion. Mol Biol Cell 8: 1063–1071. 9201716 10.1091/mbc.8.6.1063 PMC305714

[44] Garnier O, Serrano V, Duharcourt S, Meyer E (2004) RNA-mediated programming of developmental genome rearrangements in *Paramecium tetraurelia* . Mol Cell Biol 24: 7370–7379. 15314149 10.1128/MCB.24.17.7370-7379.2004 PMC506981

[45] Kamath RS, Martinez-Campos M, Zipperlen P, Fraser AG, Ahringer J (2001) Effectiveness of specific RNA-mediated interference through ingested double-stranded RNA in *Caenorhabditis elegans* . Genome Biol 2: RESEARCH0002. 11178279 10.1186/gb-2000-2-1-research0002 PMC17598

[46] Arnaiz O, Sperling L (2011) ParameciumDB in 2011: new tools and new data for functional and comparative genomics of the model ciliate *Paramecium tetraurelia* . Nucleic Acids Res 39: D632–D636. 20952411 10.1093/nar/gkq918 PMC3013783

[47] Gogendeau D, Klotz C, Arnaiz O, Malinowska A, Dadlez M, et al (2008) Functional diversification of centrins and cell morphological complexity. J Cell Sci 121: 65–74. 18057024 10.1242/jcs.019414

[48] Kim D, Pertea G, Trapnell C, Pimentel H, Kelley R, et al (2013) TopHat2: accurate alignment of transcriptomes in the presence of insertions, deletions and gene fusions. Genome Biol 14: R36. 23618408 10.1186/gb-2013-14-4-r36 PMC4053844

[49] Li H, Handsaker B, Wysoker A, Fennell T, Ruan J, et al (2009) The Sequence Alignment/Map format and SAMtools. Bioinformatics 25: 2078–2079. 19505943 10.1093/bioinformatics/btp352 PMC2723002

[50] Nowacki M, Zagorski-Ostoja W, Meyer E (2005) Nowa1p and Nowa2p: novel putative RNA binding proteins involved in *trans*-nuclear crosstalk in *Paramecium tetraurelia* . Curr Biol 15: 1616–1628. 16169483 10.1016/j.cub.2005.07.033

[51] Nowak JK, Gromadka R, Juszczuk M, Jerka-Dziadosz M, Maliszewska K, et al (2011) Functional study of genes essential for autogamy and nuclear reorganization in *Paramecium* . Eukaryot Cell 10: 363–372. 21257794 10.1128/EC.00258-10 PMC3067474

[52] Finn RD, Bateman A, Clements J, Coggill P, Eberhardt RY, et al (2014) Pfam: the protein families database. Nucleic Acids Res 42: D222–230. 24288371 10.1093/nar/gkt1223 PMC3965110

