# Supplementary figures and images for: Ku-Mediated Coupling of DNA Cleavage and Repair during Programmed Genome Rearrangements in the Ciliate Paramecium tetraurelia
Source: PLoS Genet. 2014 Aug 28;10(8):e1004552. doi: 10.1371/journal.pgen.1004552 (PMC4148214; doi:10.1371/journal.pgen.1004552)

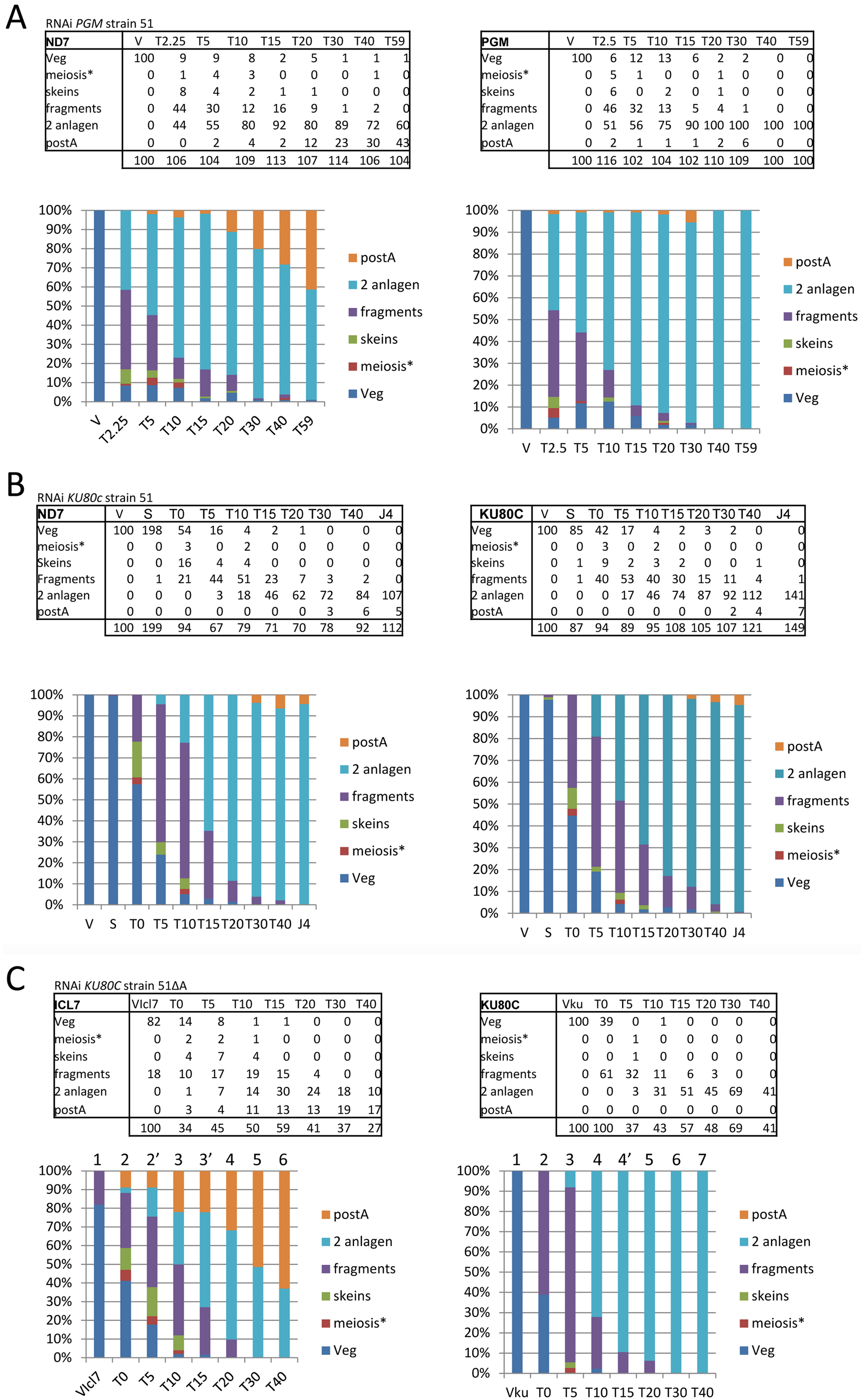

Supplement: Figure S1 — Progression of autogamy in the cultures used in this study. For each time-point, cell stages were monitored by DAPI staining. Veg: vegetative cells. Skeins: cells with elongating old MAC at the beginning of fragmentation. Meiosis*: cells with detectable meiotic MICs (which is an underestimate of the actual fraction of meiotic cells). Fragments: cells with fully fragmented old MAC, but no visible new MAC (too small or indistinguishable from fragments). 2 anlagen: cells with two visible new developing MACs. postA: post autogamous cells with one new MAC and a few fragments of the old MAC. (A) Autogamy time course of strain 51 subjected to RNAi against ND7 or PGM. The ND7 and PGM-1 constructs used for RNAi were described in [15]. (B) Autogamy time course of strain 51 subjected to RNAi against ND7 or KU80c (using the KU80c-2 construct, see Figure S2). (C) Autogamy time course of strain 51ΔA subjected to RNAi against ICL7a (RNAi construct described in [47]) or KU80c (RNAi construct: KU80c-2). Note that in the ICL7 RNAi, a fraction of cells underwent autogamy prematurely, accounting for the presence of 20% of cells with fragmented MACs in time-point 1 (VIcl7) and for the detection of 10% post-autogamous cells (postA) in the T0 sample. Taking into account the asynchrony of the ICL7 RNAi experiment, the corresponding stages between the ICL7 and KU80c RNAi experiments were numbered from 1 to 6 (intermediate stages were labeled with a′). (TIF) [file pgen.1004552.s001.tif]

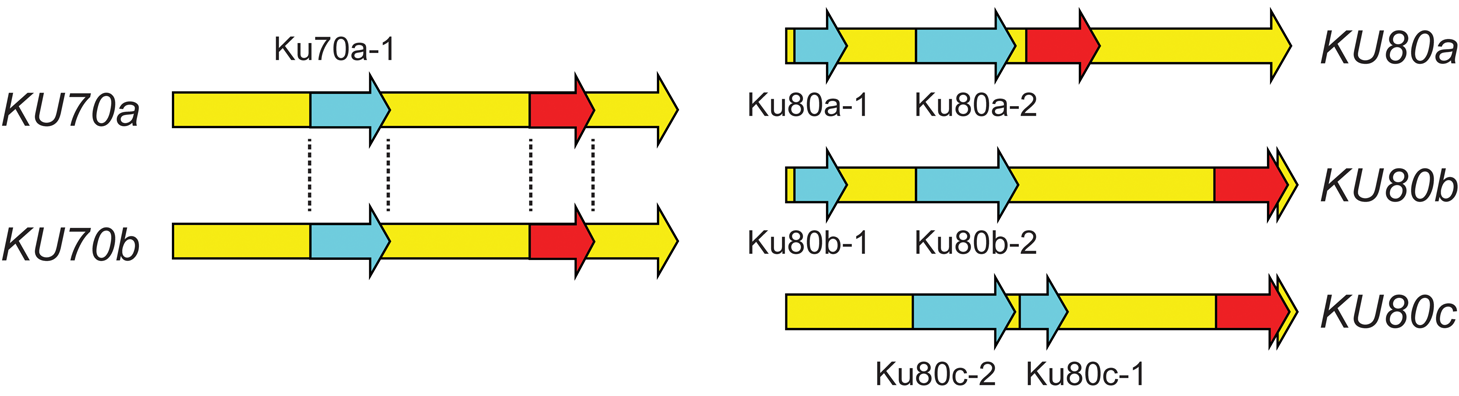

Supplement: Figure S2 — Maps of the KU70 and KU80 genes of P. tetraurelia. The maps show the position of the inserts used for RNAi constructs (blue) and hybridization probes (red, see Table S1). For KU70 genes, all fragments were designed from the KU70a sequence. Specific fragments were designed for each KU80 gene: all experiments described in the paper were performed using inserts KU80-a2, KU80-b2 and KU80-c2. Qualitatively similar phenotypes (survival/lethality in sexual progeny and presence/absence of de novo IES excision junctions) were observed when RNAi was performed using inserts KU80-a1 (which also targets KU80b mRNA) and KU80-c1. (TIF) [file pgen.1004552.s002.tif]

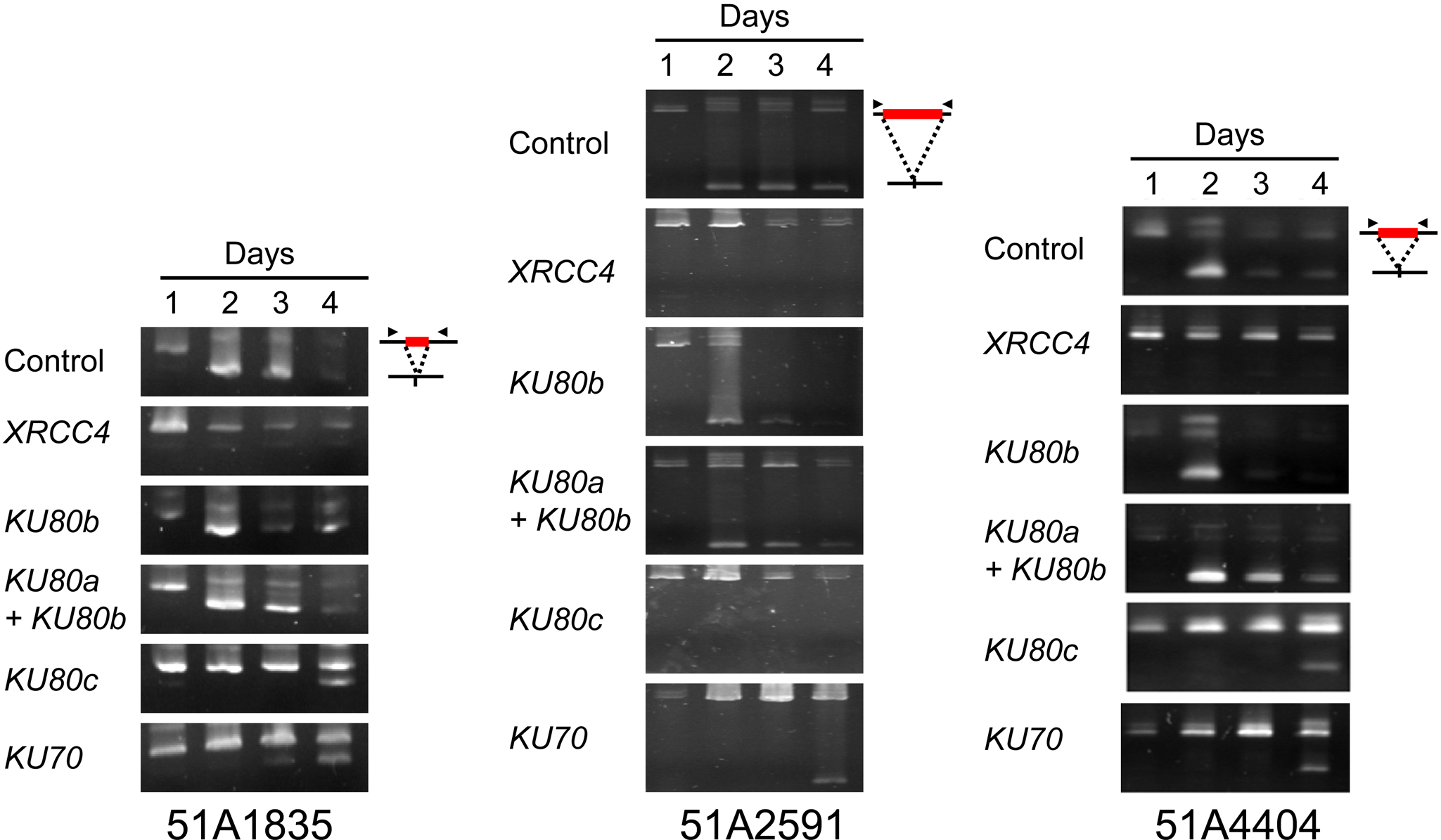

Supplement: Figure S3 — Detection of de novo IES excision junctions in different RNAi conditions. The excision of IESs 51A1835, 51A2591 and 51A4404 during autogamy of strain 51ΔA (4 days of starvation) was tested by PCR amplification (PCR primers are displayed in Table S1). RNAi conditions are indicated next to each panel. Here, starvation was prolonged for one additional day relative to the other experiments shown in the paper. Therefore, day 4 corresponds to approximately 20 hours following time-point 6 in Figure 4. The low levels of IES− forms that are detected very late during autogamy might be attributable to the recovery of significant amounts of full-length KU80c mRNA upon prolonged starvation (see Figure 3C and related text). (TIF) [file pgen.1004552.s003.tif]

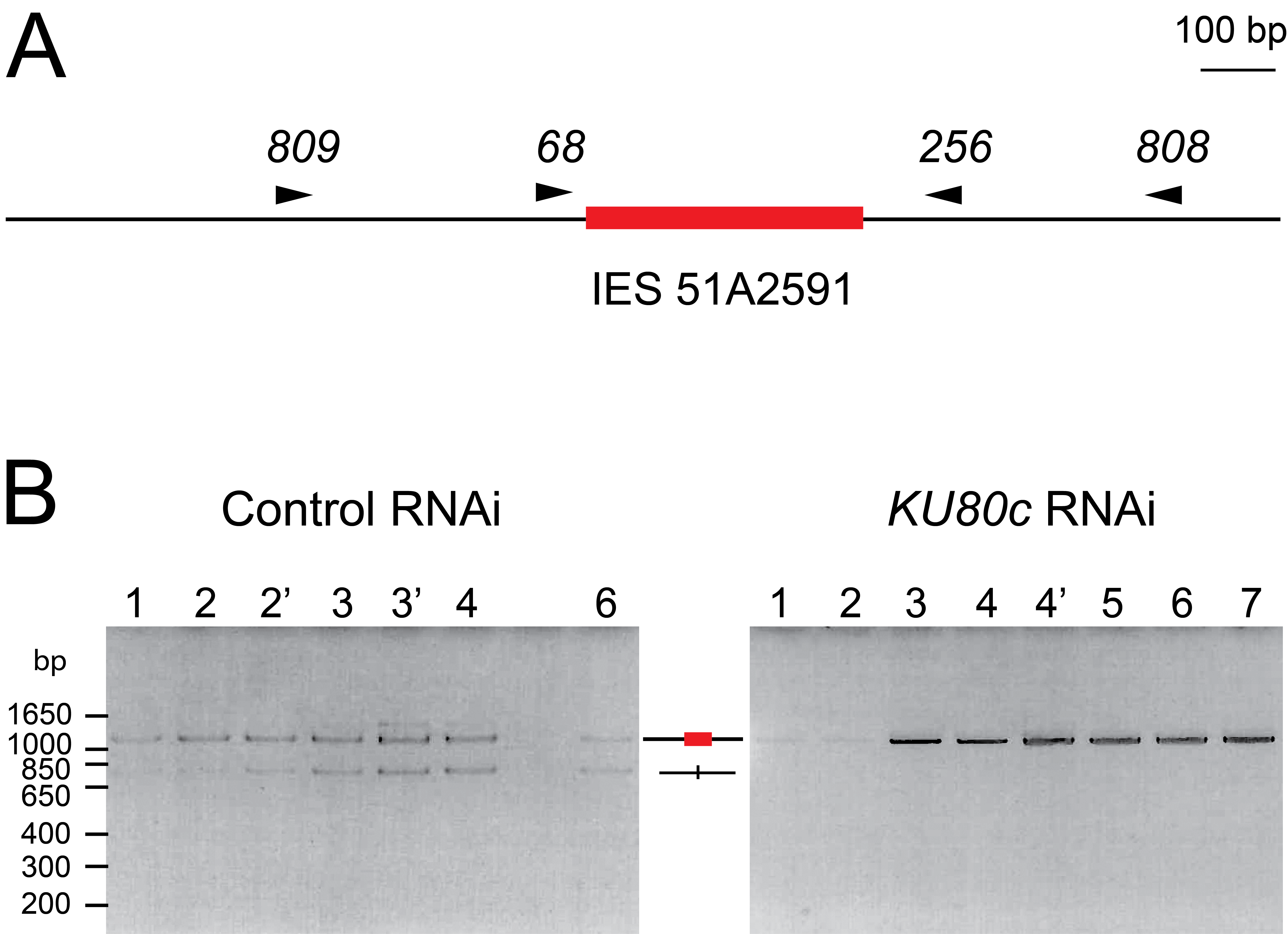

Supplement: Figure S4 — Search for alternative excision junctions for IES 51A2591. (A) Map of IES 51A2591 and its flanking MAC-destined sequences. The primers used for PCR reactions are indicated by arrowheads (see Table S1). (B) No detection of alternative de novo IES excision junctions during autogamy of 51ΔA cells, in an ICL7 (control) or a KU80c RNAi. PCR reactions were performed using primers OMB808 and OMB809 and loaded on a 2% agarose gel. (TIF) [file pgen.1004552.s004.tif]
