# Supplementary material for: Ku-Mediated Coupling of DNA Cleavage and Repair during Programmed Genome Rearrangements in the Ciliate Paramecium tetraurelia
Source: PLoS Genet. 2014 Aug 28;10(8):e1004552. doi: 10.1371/journal.pgen.1004552 (PMC4148214; doi:10.1371/journal.pgen.1004552)
Supplement: Figure S5 — Alignments of the Ku70 and Ku80 homologs from P. tetraurelia. All sequences were retrieved from the ParameciumDB database, using the following accession numbers: GSPATP00006445001 (Ku70a), GSPATP00009747001 (Ku70b), GSPATP00034664001 (Ku80a), GSPATP00035446001 (Ku80b), GSPATP00030095001 (Ku80c). Alignments of Ku80 (panel A) and Ku70 homologs (panel B) were performed separately using the MUSCLE multiple sequence alignment software (http://www.ebi.ac.uk/Tools/msa/muscle/) and colored using the BoxShade server (http://www.ch.embnet.org/software/BOX_form.html). For each Ku subunit, domain annotation was based on Pfam analysis of conserved domains (http://pfam.xfam.org/ and [52]). (PDF) [file pgen.1004552.s005.pdf]

Figure S5A: Alignment of Ku80a, Ku80b and Ku80c proteins from *P. tetraurelia*

```
Pt-Ku80c     1 MSGKEATLILLDVGASMYGQYQQGGSKKLSRLELAVDCLGLMIQQKIFNYKNHEVGLILF
Pt-Ku80a     1 MAGKEATLVLLDVGASMYEPYKQAQGKKITRLELAVDCIGMMIQQKIFNYKNHEVGLVLF
Pt-Ku80b     1 MAGKEATLVLLDVGASMYEPYKQAQGKNITRLELAVDCIGMMIQQKIFNYKNHEVGLVLF

                                    α/β domain
Pt-Ku80c    61 GTEEAPDGKTLYIQDLSIPDLDFFRNISDLPNHDVGQQVGGDIFDALDKAVHALDDHAKT
Pt-Ku80a    61 GTEDAEDGNTFYIQTLSSPDLEFYRNLTELPNHDIPKIIGGDIFDALDKSVSTLDEYVKT
Pt-Ku80b    61 GTEEAEDGNTFYIQVMSPPDLEFYRNLTELPNHDVPKIKGGDIFDALDKAVSTLDEYVKA

Pt-Ku80c   121 KKMEKKIFILTAGCGQTDYSEKQITKLIKMIEKVDVKINFIALDFMNDYNGDMDDPEKPE
Pt-Ku80a   121 KKMDKKIFVLTAGFGQTEYNEKKIAKLIKMIEKVDVKINFIALDFMNEYDAELDDPSKPE
Pt-Ku80b   121 KKMEKKIFVLTAGFGQTDYNEKKIGKLIKMIEKVDVKINFIAMDFMNEYDVELDDPTKPE

Pt-Ku80c   181 EFEALNNRMLTASYQCQEQSINSRYVFLMVQELRNNMRIFPANVAFELYSQFHTRSLQAR
Pt-Ku80a   181 NQETLNDRMLNAVYESQEQSINSRLVYYMVQELRSHMRIFPANIAFELYSQFHTKQMQAR
Pt-Ku80b   181 NQATLNDRMLNAVYQHQEQSINSRLIYQMVQELRNHMRIFPANIAFELYSQFHTKQMQAR

Pt-Ku80c   241 ASFRGDFQINDEISVQVLIYKRCFEERLPTLRKHSTLGEFQTDTNKNHVRNDLIYYNPED
Pt-Ku80a   241 ASFRGDFQINDETSISVLVYKRCTEEKLPSLKKHSATGEFSSEPTRNVVRNDTIHYNPED
Pt-Ku80b   241 ASFRGDFQINDETSIQVLIYKRCAEDRLPSLKKHSAIGEYSSEPTRNIVRNDSIHYNPED

                                     β barrel
Pt-Ku80c   301 PNMTPIEKDNIIRGYQYGRNLVPVDQIMEDKMKYQCPRQFQLLGFVDRSHIPRYYYTSTV
Pt-Ku80a   301 PNMTPIERENIIKGYLYGRSLIPVDSIMEDKMKYQCVRSFQLLGFVDKSQIPRHYFMSSV
Pt-Ku80b   301 PNMNPIERENIIKGYQYGRNLIPVDSLMEEKMKYQCNRSFQLLGFVERSQIPRHYFIFNV

Pt-Ku80c   361 DMVIAVENQKQQKALAALVIALIATRKVALARFVGREKTAPKLIMLLPHKSKNSQCFWMI
Pt-Ku80a   361 DMVVAIDCEKAKKSLSSLIIALIATKKIAIARFVGREKSSPKMVVLLPHKSKSYQCFWMI
Pt-Ku80b   361 DMVIAIDCEKARKSLSALIIALIATKKVAIARFVGRQKSSPKLMLLLPHKSKSYQCFWMI

Pt-Ku80c   421 SLPTTEDIRHFQFAALKRSTPPQQMAVSAMIDCMDLEKMPTEDGQFEELLKMKYVANPTR
Pt-Ku80a   421 ALPTSEDIRHFQFAALRKSTPHQQIAVASLIDKMDLEALPNESGEPEELLKMKYIANPTR
Pt-Ku80b   421 SLPTSEDIRHFQFSTLRKSTPNQQSAVATLIDKMNLETIPNESGEPEELLKMKYIANPTR

Pt-Ku80c   481 QYFQQVVMHKAITRSDVLPPISPLILEYLHPEKRVYDYAKEALQKVKAAFKFKINEIKKQ
Pt-Ku80a   481 QYFQQVVMHKAITRTDVIPPISPLILEYLHPEQRVYNYAQDAIQRVKNAFKFKVNEIKKP
Pt-Ku80b   481 QYFQQVVMHKAITRTDVIPPISPLILDYLHPERRVYNYAQDAIQRVKNAFKFKVNDIKKP

Pt-Ku80c   541 GDKKVFWKQLFEDQST..EQIQQQVEDEVVEINQEEEEMVNMFAKQKLGFNDDIVKEIGT
Pt-Ku80a   541 QDKKVFWKQLFDEQTTQQQQAQQQIEEEVVEINREEEEMVNMFAKQKLGFNDDIIQEIGS
Pt-Ku80b   541 QDKKVFWKQLFDEQTA.QQQVPEQIEEEIVEINREEEEMVNMFAKQKFGFNDDIIQEIGS

                          C-ter (DNA-PKcs binding)
Pt-Ku80c   599 VDPTSDFRKMITEKRVDLVDTALQQIQKVIIQFVDQSLKGSFYPKALECLKEMRKACITE
Pt-Ku80a   601 VDPISDFKKMITEKRVDLVDSALQQIQKVIIGLVDQSVKGSFFPKALECLKEMRRACISE
Pt-Ku80b   600 VDPISDFKKMITEKRVDLVDSALQQIQKVINALVDQSVKGSFFPKALECLKEMRKACISE

Pt-Ku80c   659 DEAPVFNKYLHVLKEKYSQLVFWAQIVQQGITLISNIENQKSHVSVDEAQEFLNKEDISH
Pt-Ku80a   661 DEAPVFNKFLFVLKDKYNQSIFWAQIVQQGITLISNIENQKSGVTAEEAQDFLNKEDNKH
Pt-Ku80b   660 DEAPVFNKFLFVLKDKYNQQLFWAQIVQQGITLISDIENHKSSVTAEEAQDFLNKEDNKH
```

```
Pt-Ku80c  719 KQLVDQLQHEEEDLLAEID
Pt-Ku80a  721 QQMVDQLQHEEEDLLADIE
Pt-Ku80b  720 QQMVDQLQNEEEDLLADIE
```

Figure S5B: Alignment of Ku70a and Ku70b proteins from *P. tetraurelia*

```
                                                                ----------------------
PtKu70a     1 MDFEEEHAGGDDVEEIQDGEDIFGEDADISVHDSGSKKDAVIFLVDCKKALFDMDQDGQG
PtKu70b     1 MDFEEEHAGGDDVEEIQDGEDIFGEDADISVHDSGSKKDAVIFLVDCKKALFDMDQDGQG

              ------------------------------------------------------------
PtKu70a    61 TVFSKILSAFSSFMKAKIISSPDDRIGMIFYNTKSTNNQLKFNNITEIYKLDGPSADIIK
PtKu70b    61 TVFSKILSAFSSFMKAKIISSPDDRIGMIFYNTKSTNNQLKFNNITEIYKLDGPSADIIK

              ------------------------- α/β domain -------------------------
PtKu70a   121 NCLKIEQNFEKDYQLGNNAHFHECLWLCNHEFKELDKNKFNMRIFLFTPDDLPYFKDLNA
PtKu70b   121 NCLKIEQNFEKDYQLGNNAHFHECLWLCNHEFKELDKNKFNMRIFLFTPDDLPYFKDVNA

              ------------------------------------------------------------
PtKu70a   181 RSSALKYAKQLKDADVQIELFPLPSQNEFKIARFYGEIITVDLDEVNNAVLDTSTKIMDL
PtKu70b   181 RSSALKYAKQLKDADVQIELFPLPSQNEFKIARFYGEIITVDLDEVNNAVLDTSTKIMDL

              -------------         ---------------------------------------
PtKu70a   241 HQRIKQKEFKKRALNRLIMDIDDIKIGLKIYCLVNKAKKPYGKPLDRRYNQQLKKKAQFI
PtKu70b   241 HQRIKQKEFKKRALNRLIMDIDDIKIGLKIYCLVNKAKKPYGKPLDRRYNQQLKKKAQFI

              ------------------------- β barrel ---------------------------
PtKu70a   301 DEETGQALFPQQISTHLILGNEKIAIPKEYMAKIKGFEKPGMTLIGFKSSSALKDYHNYR
PtKu70b   301 DEETGQALFPQQISTHLILGNEKIAIPKEYMAKIKGFEKPGMTLIGFKSSSALKDYHNYR

              ------------------------------------------------------------
PtKu70a   361 ASYFLYPDDEHVNGSSQFFDALIQQMILKEKIGIVRLVPKQGSQVRFCALLPQAEQYDEN
PtKu70b   361 ASYFLYPDDEHVNGSSQFFDALIQQMILKERIGIVRLVPKQGSQVRFCALLPQAEQYDEN

              ----------------------------------------           ----------
PtKu70a   421 HFQTPPGLHLIFLPYADDIRGLSTVKQEGAEITRQTLNAAKILVNALTIQDFDCSNFEDP
PtKu70b   421 HFQTPPGLHLIFLPYADDIRGLSSVKQEGAEISRQTLNAAKILVNALTIQDFDCSNFEDP

              ---------------------- C ter domain ----------
PtKu70a   481 SIQKFYTYLQGLALQEQNIEEPEDLLQPDFKGMEKYRDIVNLFMSNVSLECSNMPSRSKG
PtKu70b   481 SIQKFYTYLQGLALQEQNIEEPEDLLQPDFKGMEKYRDIVNLFMSNVSLECSNMPSRSKG

PtKu70a   541 QGGGRGRGRGRGRGRGKQEESESDDCSKVKGRGRGSTQKQKI.EEDDSLEGEEIYQPVKKRG
PtKu70b   541 QGGGRGRGRGRGRGRGKQEESDSDDCHKVKGRGRGSTSKQKVEEEEDSLEGEEIYQPIKKRG

PtKu70a   600 RGR
PtKu70b   601 RGR
```
