# Supplementary material for: Ku-Mediated Coupling of DNA Cleavage and Repair during Programmed Genome Rearrangements in the Ciliate Paramecium tetraurelia
Source: PLoS Genet. 2014 Aug 28;10(8):e1004552. doi: 10.1371/journal.pgen.1004552 (PMC4148214; doi:10.1371/journal.pgen.1004552)
Supplement: Table S1 — Oligonucleotides used in this study. (PDF) [file pgen.1004552.s006.pdf]

Table S1. Oligonucleotides used in this study

name	sequence (5' to 3')	use
ku70A-BamHI_1	gaagacaggatccAGATAAGAACAAATTCAATATGCGTA	PCR feeding insert KU70
ku70A-KpnI_1	gaagacaggtaccTTCAAAGCTCTTTTCTTAAATTCCT	PCR feeding insert KU70
ku80A-BamHI_1	gaagacaggatccGGTGCCTCAATGTATGAACCATACA	PCR feeding insert KU80a-1
ku80A-KpnI_1	gaagacaggtaccGGTAATTCTGTCAGATTTCTATAAAAC	PCR feeding insert KU80a-1
OMB223	ggactagTACGAAAGTTAAGAGCAATCAATCAATTC	PCR feeding insert KU80a-2
OMB224	ggactagtCAATAATTAAAATGACCTAACACATTAATAC	PCR feeding insert KU80a-2
OMB219	ggactagtGGTGCCTCAATGTATGAACCATACAAGTAG	PCR feeding insert KU80b-1
OMB220	ggactagtGGTAATTCTGTCAGATTTCTATAAAACTC	PCR feeding insert KU80b-1
OMB221	ggactagTACTAACATTAAGAACAATCAATAAATTC	PCR feeding insert KU80b-2
OMB222	ggactagtAAGCAATTAAAATGATCTATTACATTAATAC	PCR feeding insert KU80b-2
ku80C-BamHI_1	gaagacaggatccTAATTATTAGGCTTTGTAGATCGATC	PCR feeding insert KU80c-1
ku80C-KpnI_1bis	gaagacaggtaccTGGGGTAATAACATAATCAATTTAGGT	PCR feeding inserts KU80c-1 & KU80c-2
OMB225	ggactagtATAATAGAATGCTTACAGCTTCATATCAATG	PCR feeding insert KU80c-2
OMB063	agacaagtagggaatccacttctagtaatc	PCR around IES 51A1835
OMB064	taatgtattgataaggcttgctctacagcc	PCR around IES 51A1835
OMB068	acaccaagcgaaacatgcacagtcg	PCR around IES 51A2591
OMB256	GATGTAGCATAACATTTATCAACAATCCAT	PCR around IES 51A2591
OMB808	CAAGTTGCTCATCACTTATTTAATCAAATTG	PCR around IES 51A2591 (for alternative junctions)
OMB809	CATAATCCAGCTGTAGATCTAAAGC	PCR around IES 51A2591 (for alternative junctions)
OMB069	ccagttattgaactgcaacttactgcagtg	PCR around IES 51A4404
OMB097	TAAATGTTCAGCTTACAACGCAGCT	PCR around IES 51A4404
OMB365	AGATTTATATCTTTTTTCTCAAATTCAGC	IES circle 51A2591
OMB184	CAATATTATACATCTAGAACTTATAGTTAG	IES circle 51A2591
OMB181	TTTTGAAATATTTTCAAGTTTTTGGACTAC	IES circle 51G4404
OMB182	ACAATATATATTTACTTGATAATATTTTCC	IES circle 51G4404
OMB062	gtagtacaagatttttcgacacaagttgag	LMPCR/TdT 51A1835 MAC end
OMB065	ggttgcgtaacacttcctcttaaatgtgag	LMPCR/TdT 51A1835 MAC end
OMB066	gaagtctaatggataaccttgtggatggac	LMPCR/TdT 51A1835 MAC end
OMB145	AATTGTAAATTGACTTCAGCAAATAAAAAA	LMPCR/TdT 51A2591 MAC end
OMB212	ATGTGTTTGGACTGGATTGGCATGTAGAAG	LMPCR/TdT 51A2591 MAC end
OMB215	AGTTCCTTTGAAAGATGTGCAAGCTCCAGA	LMPCR/TdT 51A2591 MAC end
OMB069	ccagttattgaactgcaacttactgcagtg	LMPCR/TdT 51A4404 MAC end
OMB070	tggaatagtgctgcatcaccagctgcttgc	LMPCR/TdT 51A4404 MAC end
OMB226	ACCAGCTGCTTGCATTCAAATATCCACAGT	LMPCR/TdT 51A4404 MAC end
OMB113	TGCATATGTTACTGGAACTGGATTGGTAGC	LMPCR 51G4404 MAC end
OMB114	ACTGTTGCTACACATTGTGCATATGTTACT	LMPCR 51G4404 MAC end
OMB213	GCTGTAAGATTAACATTGAGCATGATCAAG	LMPCR 51G4404 MAC end
OMB300	AAAGGCTAATTTGGATGAATGAGCATTAAATC	LMPCR 51G4404 IES end
OMB301	GGACTACTTTTGAAATTGAATTATAACAAAGGC	LMPCR 51G4404 IES end
OMB056	gaattcggatccgctcggaccgtggc	LMPCR
OMB032	gctcggaccgtggctagcattagtgagtgggggggggggggggg	TdT tailing
OMB176	ACCCGTGACTGCCATGGTAGTCCAATACA	17S probe
OMB235	GTCTAGTGTGGACATGGTTGTAGCTATTGA	KU80a probe
OMB234	TCTGGTTCTCCAGATTCGTTTGGAAGTGCT	KU80a probe
OMB233	GTTATTAATGCACTAGTTGATTAATCAGTG	KU80b probe
OMB232	TTTGTAATTAGTCCACCATTTACTAATGTTTAT	KU80b probe
OMB231	GTCATTATTTAATTTGTGGATCAATCTCTC	KU80c probe
OMB230	GTTATAATTGATCAACTAATTATTTATGACTAA	KU80c probe
OMB237	TATGGCAAACCCTTAGATAGGAGATACAAC	KU70 probe
OMB236	TTTCAATATCATTTATTGAATCAATGCATC	KU70 probe
