# Supplementary material for: Ku-Mediated Coupling of DNA Cleavage and Repair during Programmed Genome Rearrangements in the Ciliate Paramecium tetraurelia
Source: PLoS Genet. 2014 Aug 28;10(8):e1004552. doi: 10.1371/journal.pgen.1004552 (PMC4148214; doi:10.1371/journal.pgen.1004552)
Supplement: Text S1 — Plasmid sequences. This file displays the sequence of all pVL-1392 derivatives used for the construction of baculovirus vectors designed for protein expression in insect cells: pVL1392-MBP-PGM (MBP-Pgm), pVL1392-MBP (MBP tag alone), pVL1392-Ku70a-HA (Ku70a-HA) and pVL1392-HA-Ku80c (HA-Ku80c). (DOCX) [file pgen.1004552.s007.docx]

**Text S1: Plasmid sequences**

>pVL1392-MBP-PGM

AAGCTTTACTCGTAAAGCGAGTTGAAGGATCATATTTAGTTGCGTTTATG AGATAAGATTGAAAGCACGTGTAAAATGTTTCCCGCGCGTTGGCACAACT ATTTACAATGCGGCCAAGTTATAAAAGATTCTAATCTGATATGTTTTAAA ACACCTTTGCGGCCCGAGTTGTTTGCGTACGTGACTAGCGAAGAAGATGT GTGGACCGCAGAACAGATAGTAAAACAAAACCCTAGTATTGGAGCAATAA TCGATTTAACCAACACGTCTAAATATTATGATGGTGTGCATTTTTTGCGG GCGGGCCTGTTATACAAAAAAATTCAAGTACCTGGCCAGACTTTGCCGCC TGAAAGCATAGTTCAAGAATTTATTGACACGGTAAAAGAATTTACAGAAA AGTGTCCCGGCATGTTGGTGGGCGTGCACTGCACACACGGTATTAATCGC ACCGGTTACATGGTGTGCAGATATTTAATGCACACCCTGGGTATTGCGCC GCAGGAAGCCATAGATAGATTCGAAAAAGCCAGAGGTCACAAAATTGAAA GACAAAATTACGTTCAAGATTTATTAATTTAATTAATATTATTTGCATTC TTTAACAAATACTTTATCCTATTTTCAAATTGTTGCGCTTCTTCCAGCGA ACCAAAACTATGCTTCGCTTGCTCCGTTTAGCTTGTAGCCGATCAGTGGC GTTGTTCCAATCGACGGTAGGATTAGGCCGGATATTCTCCACCACAATGT TGGCAACGTTGATGTTACGTTTATGCTTTTGGTTTTCCACGTACGTCTTT TGGCCGGTAATAGCCGTAAACGTAGTGCCGTCGCGCGTCACGCACAACAC CGGATGTTTGCGCTTGTCCGCGGGGTATTGAACCGCGCGATCCGACAAAT CCACCACTTTGGCAACTAAATCGGTGACCTGCGCGTCTTTTTTCTGCATT ATTTCGTCTTTCTTTTGCATGGTTTCCTGGAAGCCGGTGTACATGCGGTT TAGATCAGTCATGACGCGCGTGACCTGCAAATCTTTGGCCTCGATCTGCT TGTCCTTGATGGCAACGATGCGTTCAATAAACTCTTGTTTTTTAACAAGT TCCTCGGTTTTTTGCGCCACCACCGCTTGCAGCGCGTTTGTGTGCTCGGT GAATGTCGCAATCAGCTTAGTCACCAACTGTTTGCTCTCCTCCTCCCGTT GTTTGATCGCGGGATCGTACTTGCCGGTGCAGAGCACTTGAGGAATTACT TCTTCTAAAAGCCATTCTTGTAATTCTATGGCGTAAGGCAATTTGGACTT CATAATCAGCTGAATCACGCCGGATTTAGTAATGAGCACTGTATGCGGCT GCAAATACAGCGGGTCGCCCCTTTTCACGACGCTGTTAGAGGTAGGGCCC CCATTTTGGATGGTCTGCTCAAATAACGATTTGTATTTATTGTCTACATG AACACGTATAGCTTTATCACAAACTGTATATTTTAAACTGTTAGCGACGT CCTTGGCCACGAACCGGACCTGTTGGTCGCGCTCTAGCACGTACCGCAGG TTGAACGTATCTTCTCCAAATTTAAATTCTCCAATTTTAACGCGAGCCAT TTTGATACACGTGTGTCGATTTTGCAACAACTATTGTTTTTTAACGCAAA CTAAACTTATTGTGGTAAGCAATAATTAAATATGGGGGAACATGCGCCGC TACAACACTCGTCGTTATGAACGCAGACGGCGCCGGTCTCGGCGCAAGCG GCTAAAACGTGTTGCGCGTTCAACGCGGCAAACATCGCAAAAGCCAATAG TACAGTTTTGATTTGCATATTAACGGCGATTTTTTAAATTATCTTATTTA ATAAATAGTTATGACGCCTACAACTCCCCGCCCGCGTTGACTCGCTGCAC CTCGAGCAGTTCGTTGACGCCTTCCTCCGTGTGGCCGAACACGTCGAGCG GGTGGTCGATGACCAGCGGCGTGCCGCACGCGACGCACAAGTATCTGTAC ACCGAATGATCGTCGGGCGAAGGCACGTCGGCCTCCAAGTGGCAATATTG GCAAATTCGAAAATATATACAGTTGGGTTGTTTGCGCATATCTATCGTGG CGTTGGGCATGTACGTCCGAACGTTGATTTGCATGCAAGCCGAAATTAAA TCATTGCGATTAGTGCGATTAAAACGTTGTACATCCTCGCTTTTAATCAT GCCGTCGATTAAATCGCGCAATCGAGTCAAGTGATCAAAGTGTGGAATAA TGTTTTCTTTGTATTCCCGAGTCAAGCGCAGCGCGTATTTTAACAAACTA GCCATCTTGTAAGTTAGTTTCATTTAATGCAACTTTATCCAATAATATAT TATGTATCGCACGTCAAGAATTAACAATGCGCCCGTTGTCGCATCTCAAC ACGACTATGATAGAGATCAAATAAAGCGCGAATTAAATAGCTTGCGACGC AACGTGCACGATCTGTGCACGCGTTCCGGCACGAGCTTTGATTGTAATAA GTTTTTACGAAGCGATGACATGACCCCCGTAGTGACAACGATCACGCCCA AAAGAACTGCCGACTACAAAATTACCGAGTATGTCGGTGACGTTAAAACT ATTAAGCCATCCAATCGACCGTTAGTCGAATCAGGACCGCTGGTGCGAGA AGCCGCGAAGTATGGCGAATGCATCGTATAACGTGTGGAGTCCGCTCATT AGAGCGTCATGTTTAGACAAGAAAGCTACATATTTAATTGATCCCGATGA TTTTATTGATAAATTGACCCTAACTCCATACACGGTATTCTACAATGGCG GGGTTTTGGTCAAAATTTCCGGACTGCGATTGTACATGCTGTTAACGGCT CCGCCCACTATTAATGAAATTAAAAATTCCAATTTTAAAAAACGCAGCAA GAGAAACATTTGTATGAAAGAATGCGTAGAAGGAAAGAAAAATGTCGTCG ACATGCTGAACAACAAGATTAATATGCCTCCGTGTATAAAAAAAATATTG AACGATTTGAAAGAAAACAATGTACCGCGCGGCGGTATGTACAGGAAGAG GTTTATACTAAACTGTTACATTGCAAACGTGGTTTCGTGTGCCAAGTGTG AAAACCGATGTTTAATCAAGGCTCTGACGCATTTCTACAACCACGACTCC AAGTGTGTGGGTGAAGTCATGCATCTTTTAATCAAATCCCAAGATGTGTA TAAACCACCAAACTGCCAAAAAATGAAAACTGTCGACAAGCTCTGTCCGT TTGCTGGCAACTGCAAGGGTCTCAATCCTATTTGTAATTATTGAATAATA AAACAATTATAAATGCTAAATTTGTTTTTTATTAACGATACAAACCAAAC GCAACAAGAACATTTGTAGTATTATCTATAATTGAAAACGCGTAGTTATA ATCGCTGAGGTAATATTTAAAATCATTTTCAAATGATTCACAGTTAATTT GCGACAATATAATTTTATTTTCACATAAACTAGACGCCTTGTCGTCTTCT TCTTCGTATTCCTTCTCTTTTTCATTTTTCTCCTCATAAAAATTAACATA GTTATTATCGTATCCATATATGTATCTATCGTATAGAGTAAATTTTTTGT TGTCATAAATATATATGTCTTTTTTAATGGGGTGTATAGTACCGCTGCGC ATAGTTTTTCTGTAATTTACAACAGTGCTATTTTCTGGTAGTTCTTCGGA GTGTGTTGCTTTAATTATTAAATTTATATAATCAATGAATTTGGGATCGT CGGTTTTGTACAATATGTTGCCGGCATAGTACGCAGCTTCTTCTAGTTCA ATTACACCATTTTTTAGCAGCACCGGATTAACATAACTTTCCAAAATGTT GTACGAACCGTTAAACAAAAACAGTTCACCTCCCTTTTCTATACTATTGT CTGCGAGCAGTTGTTTGTTGTTAAAAATAACAGCCATTGTAATGAGACGC ACAAACTAATATCACAAACTGGAAATGTCTATCAATATATAGTTGCTGAT ATCATGGAGATAATTAAAATGATAACCATCTCGCAAATAAATAAGTATTT TACTGTTTTCGTAACAGTTTTGTAATAAAAAAACCTATAAATATTCCGGA TTATTCATACCGTCCCACCATCGGGCGCGGATCAGATCCACTAGTATGAA AATCGAAGAAGGTAAACTGGTAATCTGGATTAACGGCGATAAAGGCTATA ACGGTCTCGCTGAAGTCGGTAAGAAATTCGAGAAAGATACCGGAATTAAA GTCACCGTTGAGCATCCGGATAAACTGGAAGAGAAATTCCCACAGGTTGC GGCAACTGGCGATGGCCCTGACATTATCTTCTGGGCACACGACCGCTTTG GTGGCTACGCTCAATCTGGCCTGTTGGCTGAAATCACCCCGGACAAAGCG TTCCAGGACAAGCTGTATCCGTTTACCTGGGATGCCGTACGTTACAACGG CAAGCTGATTGCTTACCCGATCGCTGTTGAAGCGTTATCGCTGATTTATA ACAAAGATCTGCTGCCGAACCCGCCAAAAACCTGGGAAGAGATCCCGGCG CTGGATAAAGAACTGAAAGCGAAAGGTAAGAGCGCGCTGATGTTCAACCT GCAAGAACCGTACTTCACCTGGCCGCTGATTGCTGCTGACGGGGGTTATG CGTTCAAGTATGAAAACGGCAAGTACGACATTAAAGACGTGGGCGTGGAT AACGCTGGCGCGAAAGCGGGTCTGACCTTCCTGGTTGACCTGATTAAAAA CAAACACATGAATGCAGACACCGATTACTCCATCGCAGAAGCTGCCTTTA ATAAAGGCGAAACAGCGATGACCATCAACGGCCCGTGGGCATGGTCCAAC ATCGACACCAGCAAAGTGAATTATGGTGTAACGGTACTGCCGACCTTCAA GGGTCAACCATCCAAACCGTTCGTTGGCGTGCTGAGCGCAGGTATTAACG CCGCCAGTCCGAACAAAGAGCTGGCAAAAGAGTTCCTCGAAAACTATCTG CTGACTGATGAAGGTCTGGAAGCGGTTAATAAAGACAAACCGCTGGGTGC CGTAGCGCTGAAGTCTTACGAGGAAGAGTTGGCGAAAGATCCACGTATTG CCGCCACCATGGAAAACGCCCAGAAAGGTGAAATCATGCCGAACATCCCG CAGATGTCCGCTTTCTGGTATGCCGTGCGTACTGCGGTGATCAACGCCGC CAGCGGTCGTCAGACTGTCGATGAAGCCCTGAAAGACGCGCAGACTAATT CGAGCTCGAACAACAACAACAATAACAATAACAACAACCTCGGGATCGAG GGAAGGGGTTCGATGTTCTACAACGACGAAGAAGAAGAGGATTGGGGTGA CAAAAACGAGAAAGATGCAGAGGACGAGTTTCAGGATCTGAACCCGAGCG AGCATAAGAAACGTATCCCGAATAACCGTCCGAAGCTGCAGCCAAAACCG CAAAAGAAAAAGAAAGAGGTTCAGGAGTGCGAGTTTGCGATTGATGATCC GATTAAGCGCCGTCAGCTGGCCAATTACCAGCACGTGCCTGTGCGCTTGG AAGCGGGCGAGAAAATGAATATCAATGTCCAGTACGATGATGGCCAGGTT CGCAAGTTTGGTCAACAAAAAGCTTTCGAAGAAAAACTGATTCCAATTAG CAAATCCGGTACCTTCATCTATGAGGCTAGCACCGGCAAACTGGAGCGTA AGAACCCGGGTCGTCCTCGCGAAGATACCCGTAGCCTGTTCGATGATCCA AAACTGCAGAAGATGCGCAATAACTATGTTCCGAAACTGTTGCAACGCAT CACGACCAACAATACGAAAGAAGTTGACGAAGAAGAAGGTGTGCAGGACC CAGGTGTCTCTTGGAAGCCGGTCAAGTCCGTCCACGAGGTGCCGGAAAGC TTCTACATGCGCCAAGTGTTTGACAAGAGCGCGTCTGGTCCGCGTAGCAT CGATAAGTCTAAGATCAAATCTGAGTATGACGCCTTTCGTCTGTTCTTTG ATAATGACATCTATAACACGATCATTAAGCACACGCGTGAACGTTACCAG CAAAAAGTGGAAGAGCAAATCTATTCCTATATCCATGGCATGGTTCACAT GGGCATTCGTGCAAAGAAGCCGACGCTGATGCAGTGGGAATTCACCGAAT ACGAGCTGGAGGCGTACTTCGCTGTTCAGATCTTCTTCGGCATTGTTCGC TTGTCTAATCAACGCGACTACTGGAAAAGCTCCGCACGTCAAAAGCCGAT CAAAAAGGCGGAAACGGGTCGCCGTAAGTTGCGCGAACTGGCGCAGGAGA AAATGGACCGTTACGCGCATTGGGTGACCCAGCGTATGAGCTCTATCGTG TCCTACGAAAAGTTCAAAACGATCCGTAATTGCCTGAACATTAGCGGCGC AGAAGCGCTGAAACTGAAGGGTCGTGACCCGATCTGGAAGATTCGTGACT TTCTGAACCAGATGAACATGCGCTTCGCCAAATACTACTACCCGGGTGAA TTCATTACCATTGACGAAGGCATGATTCCGTTCGCGGGCAAGGTTCAGTT CAAAGTCTACAATCCGGATAAACCTACTAAATGGGGTATCAAAGAGTATC TGCTGTGTGATGCAAGCAATACCTACACCTTCCAACTGCGCCTGTATCAC GGCCAGACTATGTGGAACAACGATTTCAAACAAACGATGTTCGTTAATGA AGAGGACACGCAACACCGTACGATGGAGCTGGTCTTGCAAATGTGTAAAG ATTACGAGCACAAAGCCCATAAAGTGGTGATGGATAACTACTATTCGAGC TGGATGCTGTTTCGTGAGCTGCGCAACCGTGGTATCGGTGCCGTGGGTAC CATCCGCCACAATCGTACCGGCCTGACTAAGAAAGATCTGACCAGCAAGC ACTTTCAACAGATCTATAACCAATATCATTATGCGTATTACTTGAATCAA AGCAACGAACTGATGCTGATGTACTTTCAAGGTACCAGCGAGAAAGAGAT TGCATTGATTAGCAATTTCTTGGACAATAGCCTGAATGAGCAGCACATGT GGGACATTTCCAAGCAGCACTACTATGTCCCGCACCTGAAGGCACCGTAT ATGATGTACGTTTACAATAAGTACAAAGGTGGTGTTGACCGTCGTAATAG CTATGTCGTTAAGTATCGCAGCCGTTTTCCGGCGAAGAAATGGTGGCAAA GCGTTTTCGAGCGTCTGTTCGAGACTGCGATCCTGAATGCATACCTGATT TTCCGTAGCTACAACCCGGAGAGCTCTTACCGCAACAAAGGCCAGATGCG CGACTTCCGTATCAACCTGATGTATCAATTTGCCGAACGTTACAAGAGCT ATGAGCACCAGCAGGAAGAGAATGGCAAGAACCGCTTCTCGTACTTTGCG AAGATTCAACCGCATACCTTCATTGAGGGTGAAGAGATTGTGAAGTGTTC CGAGTGTGGTAATGAAACCAAAGTCTTTTGTCAAGAGTGCACCATTCTGA AAGCAGAGGTTGTGGGTCTGTGCCACGAGAAGGACACCATTAAGTGCCAA CGTTTTCATGAGTTTATGGATTTTGAGCTGGACAAAAACAAAGAAGTCAT TGATAAACGTAAGGGCAAAGATCCGTACAAGCCGAACTTTTTGGAGAAGC TGAATCAGCGTACGAACGCGAAAGGTAACCAAAGCAGCCAGAAGAAAGAA AGCCCGCTGGTGAACCTGCTGAATAAGATTAACGACCAGATCAAGCAGGA AGCTCGTGTTAAACAGGAAGTCAAACGTGAAGATAACACCAATAAGCAAA CGACCTACATCATCCCGGAGGTCAAATCGGAGGACGAGAGCTCCACCGAT AGCGATATCTACATTCAGCGTACCACGAACCAGCGCCTGATCGAGATTCA TCAGAAGATTGAGCAGATGAGCATGTGCAGCGAAGAGTTCCTGAACGGTT CGGTGGCCAACAGCCAGGAGAATTTCGCGGAACGTGATGAGAATGACCAG TTCAACGATAACAATGGTAACGACGACAATCAATTTCAGTTTCCGCAGCA ACGTGCCCAGCAGATCGACGATGATGACGAACAACGCAACAGCAAAAACG AAGAGCAGCAGAAGGACTTCATTAAGAAAATGATGGAGTTTGCGGACGAC GAGGGCTCCGAAAATGAAGAAATTCAGTATCCGGAGGATGAGGCTGATCA CTTCTACCAACAACTGCTGCAACAGGAAGAGCAGGCGATCAAGTATCAAC AAAAGAAACAGCTGCAGCAGCAACTGGAAGAAGAGTCCGAACGCAGCAAT ATCTCCCACAAGAGCAAGAAGCAACAAAAACTGGAACAAAAGTTTATCGA AACGAGCATGCGTGGTATCAAACAGAGCCAAATTCAGAGCAATAGCGAGA TCGGCCAGGACCTGCAAAAGATTATCAGCGCAAGCCAAGACCTGAATCAG ATTAGCAAACAGATCACCGAGAGCAAAGGCGATAATCAAAACAGCCAGAG CGACCAATGACTCGAGCACCACCACCACCACCACTGAGATCCGGCTGCTA ACAAAGCCCGAAAGGAAGCTGAGTTGGCTGCTGCCACCGCTGAGCGGATC CTTTCCTGGGACCCGGCAAGAACCAAAAACTCACTCTCTTCAAGGAAATC CGTAATGTTAAACCCGACACGATGAAGCTTGTCGTTGGATGGAAAGGAAA AGAGTTCTACAGGGAAACTTGGACCCGCTTCATGGAAGACAGCTTCCCCA TTGTTAACGACCAAGAAGTGATGGATGTTTTCCTTGTTGTCAACATGCGT CCCACTAGACCCAACCGTTGTTACAAATTCCTGGCCCAACACGCTCTGCG TTGCGACCCCGACTATGTACCTCATGACGTGATTAGGATCGTCGAGCCTT CATGGGTGGGCAGCAACAACGAGTACCGCATCAGCCTGGCTAAGAAGGGC GGCGGCTGCCCAATAATGAACCTTCACTCTGAGTACACCAACTCGTTCGA ACAGTTCATCGATCGTGTCATCTGGGAGAACTTCTACAAGCCCATCGTTT ACATCGGTACCGACTCTGCTGAAGAGGAGGAAATTCTCCTTGAAGTTTCC CTGGTGTTCAAAGTAAAGGAGTTTGCACCAGACGCACCTCTGTTCACTGG TCCGGCGTATTAAAACACGATACATTGTTATTAGTACATTTATTAAGCGC TAGATTCTGTGCGTTGTTGATTTACAGACAATTGTTGTACGTATTTTAAT AATTCATTAAATTTATAATCTTTAGGGTGGTATGTTAGAGCGAAAATCAA ATGATTTTCAGCGTCTTTATATCTGAATTTAAATATTAAATCCTCAATAG ATTTGTAAAATAGGTTTCGATTAGTTTCAAACAAGGGTTGTTTTTCCGAA CCGATGGCTGGACTATCTAATGGATTTTCGCTCAACGCCACAAAACTTGC CAAATCTTGTAGCAGCAATCTAGCTTTGTCGATATTCGTTTGTGTTTTGT TTTGTAATAAAGGTTCGACGTCGTTCAAAATATTATGCGCTTTTGTATTT CTTTCATCACTGTCGTTAGTGTACAATTGACTCGACGTAAACACGTTAAA TAAAGCTTGGACATATTTAACATCGGGCGTGTTAGCTTTATTAGGCCGAT TATCGTCGTCGTCCCAACCCTCGTCGTTAGAAGTTGCTTCCGAAGACGAT TTTGCCATAGCCACACGACGCCTATTAATTGTGTCGGCTAACACGTCCGC GATCAAATTTGTAGTTGAGCTTTTTGGAATTATTTCTGATTGCGGGCGTT TTTGGGCGGGTTTCAATCTAACTGTGCCCGATTTTAATTCAGACAACACG TTAGAAAGCGATGGTGCAGGCGGTGGTAACATTTCAGACGGCAAATCTAC TAATGGCGGCGGTGGTGGAGCTGATGATAAATCTACCATCGGTGGAGGCG CAGGCGGGGCTGGCGGCGGAGGCGGAGGCGGAGGTGGTGGCGGTGATGCA GACGGCGGTTTAGGCTCAAATGTCTCTTTAGGCAACACAGTCGGCACCTC AACTATTGTACTGGTTTCGGGCGCCGTTTTTGGTTTGACCGGTCTGAGAC GAGTGCGATTTTTTTCGTTTCTAATAGCTTCCAACAATTGTTGTCTGTCG TCTAAAGGTGCAGCGGGTTGAGGTTCCGTCGGCATTGGTGGAGCGGGCGG CAATTCAGACATCGATGGTGGTGGTGGTGGTGGAGGCGCTGGAATGTTAG GCACGGGAGAAGGTGGTGGCGGCGGTGCCGCCGGTATAATTTGTTCTGGT TTAGTTTGTTCGCGCACGATTGTGGGCACCGGCGCAGGCGCCGCTGGCTG CACAACGGAAGGTCGTCTGCTTCGAGGCAGCGCTTGGGGTGGTGGCAATT CAATATTATAATTGGAATACAAATCGTAAAAATCTGCTATAAGCATTGTA ATTTCGCTATCGTTTACCGTGCCGATATTTAACAACCGCTCAATGTAAGC AATTGTATTGTAAAGAGATTGTCTCAAGCTCGCCGCACGCCGATAACAAG CCTTTTCATTTTTACTACAGCATTGTAGTGGCGAGACACTTCGCTGTCGT CGACGTACATGTATGCTTTGTTGTCAAAAACGTCGTTGGCAAGCTTTAAA ATATTTAAAAGAACATCTCTGTTCAGCACCACTGTGTTGTCGTAAATGTT GTTTTTGATAATTTGCGCTTCCGCAGTATCGACACGTTCAAAAAATTGAT GCGCATCAATTTTGTTGTTCCTATTATTGAATAAATAAGATTGTACAGAT TCATATCTACGATTCGTCATGGCCACCACAAATGCTACGCTGCAAACGCT GGTACAATTTTACGAAAACTGCAAAAACGTCAAAACTCGGTATAAAATAA TCAACGGGCGCTTTGGCAAAATATCTATTTTATCGCACAAGCCCACTAGC AAATTGTATTTGCAGAAAACAATTTCGGCGCACAATTTTAACGCTGACGA AATAAAAGTTCACCAGTTAATGAGCGACCACCCAAATTTTATAAAAATCT ATTTTAATCACGGTTCCATCAACAACCAAGTGATCGTGATGGACTACATT GACTGTCCCGATTTATTTGAAACACTACAAATTAAAGGCGAGCTTTCGTA CCAACTTGTTAGCAATATTATTAGACAGCTGTGTGAAGCGCTCAACGATT TGCACAAGCACAATTTCATACACAACGACATAAAACTCGAAAATGTCTTA TATTTCGAAGCACTTGATCGCGTGTATGTTTGCGATTACGGATTGTGCAA ACACGAAAACTCACTTAGCGTGCACGACGGCACGTTGGAGTATTTTAGTC CGGAAAAAATTCGACACACAACTATGCACGTTTCGTTTGACTGGTACGCG GCGTGTTAACATACAAGTTGCTAACGTAATCATGGTCATAGCTGTTTCCT GTGTGAAATTGTTATCCGCTCACAATTCCACACAACATACGAGCCGGAAG CATAAAGTGTAAAGCCTGGGGTGCCTAATGAGTGAGCTAACTCACATTAA TTGCGTTGCGCTCACTGCCCGCTTTCCAGTCGGGAAACCTGTCGTGCCAG CTGCATTAATGAATCGGCCAACGCGCGGGGAGAGGCGGTTTGCGTATTGG GCGCTCTTCCGCTTCCTCGCTCACTGACTCGCTGCGCTCGGTCGTTCGGC TGCGGCGAGCGGTATCAGCTCACTCAAAGGCGGTAATACGGTTATCCACA GAATCAGGGGATAACGCAGGAAAGAACATGTGAGCAAAAGGCCAGCAAAA GGCCAGGAACCGTAAAAAGGCCGCGTTGCTGGCGTTTTTCCATAGGCTCC GCCCCCCTGACGAGCATCACAAAAATCGACGCTCAAGTCAGAGGTGGCGA AACCCGACAGGACTATAAAGATACCAGGCGTTTCCCCCTGGAAGCTCCCT CGTGCGCTCTCCTGTTCCGACCCTGCCGCTTACCGGATACCTGTCCGCCT TTCTCCCTTCGGGAAGCGTGGCGCTTTCTCATAGCTCACGCTGTAGGTAT CTCAGTTCGGTGTAGGTCGTTCGCTCCAAGCTGGGCTGTGTGCACGAACC CCCCGTTCAGCCCGACCGCTGCGCCTTATCCGGTAACTATCGTCTTGAGT CCAACCCGGTAAGACACGACTTATCGCCACTGGCAGCAGCCACTGGTAAC AGGATTAGCAGAGCGAGGTATGTAGGCGGTGCTACAGAGTTCTTGAAGTG GTGGCCTAACTACGGCTACACTAGAAGGACAGTATTTGGTATCTGCGCTC TGCTGAAGCCAGTTACCTTCGGAAAAAGAGTTGGTAGCTCTTGATCCGGC AAACAAACCACCGCTGGTAGCGGTGGTTTTTTTGTTTGCAAGCAGCAGAT TACGCGCAGAAAAAAAGGATCTCAAGAAGATCCTTTGATCTTTTCTACGG GGTCTGACGCTCAGTGGAACGAAAACTCACGTTAAGGGATTTTGGTCATG AGATTATCAAAAAGGATCTTCACCTAGATCCTTTTAAATTAAAAATGAAG TTTTAAATCAATCTAAAGTATATATGAGTAAACTTGGTCTGACAGTTACC AATGCTTAATCAGTGAGGCACCTATCTCAGCGATCTGTCTATTTCGTTCA TCCATAGTTGCCTGACTCCCCGTCGTGTAGATAACTACGATACGGGAGGG CTTACCATCTGGCCCCAGTGCTGCAATGATACCGCGAGACCCACGCTCAC CGGCTCCAGATTTATCAGCAATAAACCAGCCAGCCGGAAGGGCCGAGCGC AGAAGTGGTCCTGCAACTTTATCCGCCTCCATCCAGTCTATTAATTGTTG CCGGGAAGCTAGAGTAAGTAGTTCGCCAGTTAATAGTTTGCGCAACGTTG TTGCCATTGCTACAGGCATCGTGGTGTCACGCTCGTCGTTTGGTATGGCT TCATTCAGCTCCGGTTCCCAACGATCAAGGCGAGTTACATGATCCCCCAT GTTGTGCAAAAAAGCGGTTAGCTCCTTCGGTCCTCCGATCGTTGTCAGAA GTAAGTTGGCCGCAGTGTTATCACTCATGGTTATGGCAGCACTGCATAAT TCTCTTACTGTCATGCCATCCGTAAGATGCTTTTCTGTGACTGGTGAGTA CTCAACCAAGTCATTCTGAGAATAGTGTATGCGGCGACCGAGTTGCTCTT GCCCGGCGTCAATACGGGATAATACCGCGCCACATAGCAGAACTTTAAAA GTGCTCATCATTGGAAAACGTTCTTCGGGGCGAAAACTCTCAAGGATCTT ACCGCTGTTGAGATCCAGTTCGATGTAACCCACTCGTGCACCCAACTGAT CTTCAGCATCTTTTACTTTCACCAGCGTTTCTGGGTGAGCAAAAACAGGA AGGCAAAATGCCGCAAAAAAGGGAATAAGGGCGACACGGAAATGTTGAAT ACTCATACTCTTCCTTTTTCAATATTATTGAAGCATTTATCAGGGTTATT GTCTCATGAGCGGATACATATTTGAATGTATTTAGAAAAATAAACAAATA GGGGTTCCGCGCACATTTCCCCGAAAAGTGCCACCTGACGTCTAAGAAAC CATTATTATCATGACATTAACCTATAAAAATAGGCGTATCACGAGGCCCT TTCGTCTCGCGCGTTTCGGTGATGACGGTGAAAACCTCTGACACATGCAG CTCCCGGAGACGGTCACAGCTTGTCTGTAAGCGGATGCCGGGAGCAGACA AGCCCGTCAGGGCGCGTCAGCGGGTGTTGGCGGGTGTCGGGGCTGGCTTA ACTATGCGGCATCAGAGCAGATTGTACTGAGAGTGCACCATATGCGGTGT GAAATACCGCACAGATGCGTAAGGAGAAAATACCGCATCAGGCGCCATTC GCCATTCAGGCTGCGCAACTGTTGGGAAGGGCGATCGGTGCGGGCCTCTT CGCTATTACGCCAGCTGGCGAAAGGGGGATGTGCTGCAAGGCGATTAAGT TGGGTAACGCCAGGGTTTTCCCAGTCACGACGTTGTAAAACGACGGCCAGTGCC

>pVL1392-MBP

AAGCTTTACTCGTAAAGCGAGTTGAAGGATCATATTTAGTTGCGTTTATG AGATAAGATTGAAAGCACGTGTAAAATGTTTCCCGCGCGTTGGCACAACT ATTTACAATGCGGCCAAGTTATAAAAGATTCTAATCTGATATGTTTTAAA ACACCTTTGCGGCCCGAGTTGTTTGCGTACGTGACTAGCGAAGAAGATGT GTGGACCGCAGAACAGATAGTAAAACAAAACCCTAGTATTGGAGCAATAA TCGATTTAACCAACACGTCTAAATATTATGATGGTGTGCATTTTTTGCGG GCGGGCCTGTTATACAAAAAAATTCAAGTACCTGGCCAGACTTTGCCGCC TGAAAGCATAGTTCAAGAATTTATTGACACGGTAAAAGAATTTACAGAAA AGTGTCCCGGCATGTTGGTGGGCGTGCACTGCACACACGGTATTAATCGC ACCGGTTACATGGTGTGCAGATATTTAATGCACACCCTGGGTATTGCGCC GCAGGAAGCCATAGATAGATTCGAAAAAGCCAGAGGTCACAAAATTGAAA GACAAAATTACGTTCAAGATTTATTAATTTAATTAATATTATTTGCATTC TTTAACAAATACTTTATCCTATTTTCAAATTGTTGCGCTTCTTCCAGCGA ACCAAAACTATGCTTCGCTTGCTCCGTTTAGCTTGTAGCCGATCAGTGGC GTTGTTCCAATCGACGGTAGGATTAGGCCGGATATTCTCCACCACAATGT TGGCAACGTTGATGTTACGTTTATGCTTTTGGTTTTCCACGTACGTCTTT TGGCCGGTAATAGCCGTAAACGTAGTGCCGTCGCGCGTCACGCACAACAC CGGATGTTTGCGCTTGTCCGCGGGGTATTGAACCGCGCGATCCGACAAAT CCACCACTTTGGCAACTAAATCGGTGACCTGCGCGTCTTTTTTCTGCATT ATTTCGTCTTTCTTTTGCATGGTTTCCTGGAAGCCGGTGTACATGCGGTT TAGATCAGTCATGACGCGCGTGACCTGCAAATCTTTGGCCTCGATCTGCT TGTCCTTGATGGCAACGATGCGTTCAATAAACTCTTGTTTTTTAACAAGT TCCTCGGTTTTTTGCGCCACCACCGCTTGCAGCGCGTTTGTGTGCTCGGT GAATGTCGCAATCAGCTTAGTCACCAACTGTTTGCTCTCCTCCTCCCGTT GTTTGATCGCGGGATCGTACTTGCCGGTGCAGAGCACTTGAGGAATTACT TCTTCTAAAAGCCATTCTTGTAATTCTATGGCGTAAGGCAATTTGGACTT CATAATCAGCTGAATCACGCCGGATTTAGTAATGAGCACTGTATGCGGCT GCAAATACAGCGGGTCGCCCCTTTTCACGACGCTGTTAGAGGTAGGGCCC CCATTTTGGATGGTCTGCTCAAATAACGATTTGTATTTATTGTCTACATG AACACGTATAGCTTTATCACAAACTGTATATTTTAAACTGTTAGCGACGT CCTTGGCCACGAACCGGACCTGTTGGTCGCGCTCTAGCACGTACCGCAGG TTGAACGTATCTTCTCCAAATTTAAATTCTCCAATTTTAACGCGAGCCAT TTTGATACACGTGTGTCGATTTTGCAACAACTATTGTTTTTTAACGCAAA CTAAACTTATTGTGGTAAGCAATAATTAAATATGGGGGAACATGCGCCGC TACAACACTCGTCGTTATGAACGCAGACGGCGCCGGTCTCGGCGCAAGCG GCTAAAACGTGTTGCGCGTTCAACGCGGCAAACATCGCAAAAGCCAATAG TACAGTTTTGATTTGCATATTAACGGCGATTTTTTAAATTATCTTATTTA ATAAATAGTTATGACGCCTACAACTCCCCGCCCGCGTTGACTCGCTGCAC CTCGAGCAGTTCGTTGACGCCTTCCTCCGTGTGGCCGAACACGTCGAGCG GGTGGTCGATGACCAGCGGCGTGCCGCACGCGACGCACAAGTATCTGTAC ACCGAATGATCGTCGGGCGAAGGCACGTCGGCCTCCAAGTGGCAATATTG GCAAATTCGAAAATATATACAGTTGGGTTGTTTGCGCATATCTATCGTGG CGTTGGGCATGTACGTCCGAACGTTGATTTGCATGCAAGCCGAAATTAAA TCATTGCGATTAGTGCGATTAAAACGTTGTACATCCTCGCTTTTAATCAT GCCGTCGATTAAATCGCGCAATCGAGTCAAGTGATCAAAGTGTGGAATAA TGTTTTCTTTGTATTCCCGAGTCAAGCGCAGCGCGTATTTTAACAAACTA GCCATCTTGTAAGTTAGTTTCATTTAATGCAACTTTATCCAATAATATAT TATGTATCGCACGTCAAGAATTAACAATGCGCCCGTTGTCGCATCTCAAC ACGACTATGATAGAGATCAAATAAAGCGCGAATTAAATAGCTTGCGACGC AACGTGCACGATCTGTGCACGCGTTCCGGCACGAGCTTTGATTGTAATAA GTTTTTACGAAGCGATGACATGACCCCCGTAGTGACAACGATCACGCCCA AAAGAACTGCCGACTACAAAATTACCGAGTATGTCGGTGACGTTAAAACT ATTAAGCCATCCAATCGACCGTTAGTCGAATCAGGACCGCTGGTGCGAGA AGCCGCGAAGTATGGCGAATGCATCGTATAACGTGTGGAGTCCGCTCATT AGAGCGTCATGTTTAGACAAGAAAGCTACATATTTAATTGATCCCGATGA TTTTATTGATAAATTGACCCTAACTCCATACACGGTATTCTACAATGGCG GGGTTTTGGTCAAAATTTCCGGACTGCGATTGTACATGCTGTTAACGGCT CCGCCCACTATTAATGAAATTAAAAATTCCAATTTTAAAAAACGCAGCAA GAGAAACATTTGTATGAAAGAATGCGTAGAAGGAAAGAAAAATGTCGTCG ACATGCTGAACAACAAGATTAATATGCCTCCGTGTATAAAAAAAATATTG AACGATTTGAAAGAAAACAATGTACCGCGCGGCGGTATGTACAGGAAGAG GTTTATACTAAACTGTTACATTGCAAACGTGGTTTCGTGTGCCAAGTGTG AAAACCGATGTTTAATCAAGGCTCTGACGCATTTCTACAACCACGACTCC AAGTGTGTGGGTGAAGTCATGCATCTTTTAATCAAATCCCAAGATGTGTA TAAACCACCAAACTGCCAAAAAATGAAAACTGTCGACAAGCTCTGTCCGT TTGCTGGCAACTGCAAGGGTCTCAATCCTATTTGTAATTATTGAATAATA AAACAATTATAAATGCTAAATTTGTTTTTTATTAACGATACAAACCAAAC GCAACAAGAACATTTGTAGTATTATCTATAATTGAAAACGCGTAGTTATA ATCGCTGAGGTAATATTTAAAATCATTTTCAAATGATTCACAGTTAATTT GCGACAATATAATTTTATTTTCACATAAACTAGACGCCTTGTCGTCTTCT TCTTCGTATTCCTTCTCTTTTTCATTTTTCTCCTCATAAAAATTAACATA GTTATTATCGTATCCATATATGTATCTATCGTATAGAGTAAATTTTTTGT TGTCATAAATATATATGTCTTTTTTAATGGGGTGTATAGTACCGCTGCGC ATAGTTTTTCTGTAATTTACAACAGTGCTATTTTCTGGTAGTTCTTCGGA GTGTGTTGCTTTAATTATTAAATTTATATAATCAATGAATTTGGGATCGT CGGTTTTGTACAATATGTTGCCGGCATAGTACGCAGCTTCTTCTAGTTCA ATTACACCATTTTTTAGCAGCACCGGATTAACATAACTTTCCAAAATGTT GTACGAACCGTTAAACAAAAACAGTTCACCTCCCTTTTCTATACTATTGT CTGCGAGCAGTTGTTTGTTGTTAAAAATAACAGCCATTGTAATGAGACGC ACAAACTAATATCACAAACTGGAAATGTCTATCAATATATAGTTGCTGAT ATCATGGAGATAATTAAAATGATAACCATCTCGCAAATAAATAAGTATTT TACTGTTTTCGTAACAGTTTTGTAATAAAAAAACCTATAAATATTCCGGA TTATTCATACCGTCCCACCATCGGGCGCGGATCAGATCCACTAGTATGAA AATCGAAGAAGGTAAACTGGTAATCTGGATTAACGGCGATAAAGGCTATA ACGGTCTCGCTGAAGTCGGTAAGAAATTCGAGAAAGATACCGGAATTAAA GTCACCGTTGAGCATCCGGATAAACTGGAAGAGAAATTCCCACAGGTTGC GGCAACTGGCGATGGCCCTGACATTATCTTCTGGGCACACGACCGCTTTG GTGGCTACGCTCAATCTGGCCTGTTGGCTGAAATCACCCCGGACAAAGCG TTCCAGGACAAGCTGTATCCGTTTACCTGGGATGCCGTACGTTACAACGG CAAGCTGATTGCTTACCCGATCGCTGTTGAAGCGTTATCGCTGATTTATA ACAAAGATCTGCTGCCGAACCCGCCAAAAACCTGGGAAGAGATCCCGGCG CTGGATAAAGAACTGAAAGCGAAAGGTAAGAGCGCGCTGATGTTCAACCT GCAAGAACCGTACTTCACCTGGCCGCTGATTGCTGCTGACGGGGGTTATG CGTTCAAGTATGAAAACGGCAAGTACGACATTAAAGACGTGGGCGTGGAT AACGCTGGCGCGAAAGCGGGTCTGACCTTCCTGGTTGACCTGATTAAAAA CAAACACATGAATGCAGACACCGATTACTCCATCGCAGAAGCTGCCTTTA ATAAAGGCGAAACAGCGATGACCATCAACGGCCCGTGGGCATGGTCCAAC ATCGACACCAGCAAAGTGAATTATGGTGTAACGGTACTGCCGACCTTCAA GGGTCAACCATCCAAACCGTTCGTTGGCGTGCTGAGCGCAGGTATTAACG CCGCCAGTCCGAACAAAGAGCTGGCAAAAGAGTTCCTCGAAAACTATCTG CTGACTGATGAAGGTCTGGAAGCGGTTAATAAAGACAAACCGCTGGGTGC CGTAGCGCTGAAGTCTTACGAGGAAGAGTTGGCGAAAGATCCACGTATTG CCGCCACCATGGAAAACGCCCAGAAAGGTGAAATCATGCCGAACATCCCG CAGATGTCCGCTTTCTGGTATGCCGTGCGTACTGCGGTGATCAACGCCGC CAGCGGTCGTCAGACTGTCGATGAAGCCCTGAAAGACGCGCAGACTAATT CGAGCTCGAACAACAACAACAATAACAATAACAACAACCTCGGGATCGAG GGAAGGGGTTCGTGAGGATCCTTTCCTGGGACCCGGCAAGAACCAAAAAC TCACTCTCTTCAAGGAAATCCGTAATGTTAAACCCGACACGATGAAGCTT GTCGTTGGATGGAAAGGAAAAGAGTTCTACAGGGAAACTTGGACCCGCTT CATGGAAGACAGCTTCCCCATTGTTAACGACCAAGAAGTGATGGATGTTT TCCTTGTTGTCAACATGCGTCCCACTAGACCCAACCGTTGTTACAAATTC CTGGCCCAACACGCTCTGCGTTGCGACCCCGACTATGTACCTCATGACGT GATTAGGATCGTCGAGCCTTCATGGGTGGGCAGCAACAACGAGTACCGCA TCAGCCTGGCTAAGAAGGGCGGCGGCTGCCCAATAATGAACCTTCACTCT GAGTACACCAACTCGTTCGAACAGTTCATCGATCGTGTCATCTGGGAGAA CTTCTACAAGCCCATCGTTTACATCGGTACCGACTCTGCTGAAGAGGAGG AAATTCTCCTTGAAGTTTCCCTGGTGTTCAAAGTAAAGGAGTTTGCACCA GACGCACCTCTGTTCACTGGTCCGGCGTATTAAAACACGATACATTGTTA TTAGTACATTTATTAAGCGCTAGATTCTGTGCGTTGTTGATTTACAGACA ATTGTTGTACGTATTTTAATAATTCATTAAATTTATAATCTTTAGGGTGG TATGTTAGAGCGAAAATCAAATGATTTTCAGCGTCTTTATATCTGAATTT AAATATTAAATCCTCAATAGATTTGTAAAATAGGTTTCGATTAGTTTCAA ACAAGGGTTGTTTTTCCGAACCGATGGCTGGACTATCTAATGGATTTTCG CTCAACGCCACAAAACTTGCCAAATCTTGTAGCAGCAATCTAGCTTTGTC GATATTCGTTTGTGTTTTGTTTTGTAATAAAGGTTCGACGTCGTTCAAAA TATTATGCGCTTTTGTATTTCTTTCATCACTGTCGTTAGTGTACAATTGA CTCGACGTAAACACGTTAAATAAAGCTTGGACATATTTAACATCGGGCGT GTTAGCTTTATTAGGCCGATTATCGTCGTCGTCCCAACCCTCGTCGTTAG AAGTTGCTTCCGAAGACGATTTTGCCATAGCCACACGACGCCTATTAATT GTGTCGGCTAACACGTCCGCGATCAAATTTGTAGTTGAGCTTTTTGGAAT TATTTCTGATTGCGGGCGTTTTTGGGCGGGTTTCAATCTAACTGTGCCCG ATTTTAATTCAGACAACACGTTAGAAAGCGATGGTGCAGGCGGTGGTAAC ATTTCAGACGGCAAATCTACTAATGGCGGCGGTGGTGGAGCTGATGATAA ATCTACCATCGGTGGAGGCGCAGGCGGGGCTGGCGGCGGAGGCGGAGGCG GAGGTGGTGGCGGTGATGCAGACGGCGGTTTAGGCTCAAATGTCTCTTTA GGCAACACAGTCGGCACCTCAACTATTGTACTGGTTTCGGGCGCCGTTTT TGGTTTGACCGGTCTGAGACGAGTGCGATTTTTTTCGTTTCTAATAGCTT CCAACAATTGTTGTCTGTCGTCTAAAGGTGCAGCGGGTTGAGGTTCCGTC GGCATTGGTGGAGCGGGCGGCAATTCAGACATCGATGGTGGTGGTGGTGG TGGAGGCGCTGGAATGTTAGGCACGGGAGAAGGTGGTGGCGGCGGTGCCG CCGGTATAATTTGTTCTGGTTTAGTTTGTTCGCGCACGATTGTGGGCACC GGCGCAGGCGCCGCTGGCTGCACAACGGAAGGTCGTCTGCTTCGAGGCAG CGCTTGGGGTGGTGGCAATTCAATATTATAATTGGAATACAAATCGTAAA AATCTGCTATAAGCATTGTAATTTCGCTATCGTTTACCGTGCCGATATTT AACAACCGCTCAATGTAAGCAATTGTATTGTAAAGAGATTGTCTCAAGCT CGCCGCACGCCGATAACAAGCCTTTTCATTTTTACTACAGCATTGTAGTG GCGAGACACTTCGCTGTCGTCGACGTACATGTATGCTTTGTTGTCAAAAA CGTCGTTGGCAAGCTTTAAAATATTTAAAAGAACATCTCTGTTCAGCACC ACTGTGTTGTCGTAAATGTTGTTTTTGATAATTTGCGCTTCCGCAGTATC GACACGTTCAAAAAATTGATGCGCATCAATTTTGTTGTTCCTATTATTGA ATAAATAAGATTGTACAGATTCATATCTACGATTCGTCATGGCCACCACA AATGCTACGCTGCAAACGCTGGTACAATTTTACGAAAACTGCAAAAACGT CAAAACTCGGTATAAAATAATCAACGGGCGCTTTGGCAAAATATCTATTT TATCGCACAAGCCCACTAGCAAATTGTATTTGCAGAAAACAATTTCGGCG CACAATTTTAACGCTGACGAAATAAAAGTTCACCAGTTAATGAGCGACCA CCCAAATTTTATAAAAATCTATTTTAATCACGGTTCCATCAACAACCAAG TGATCGTGATGGACTACATTGACTGTCCCGATTTATTTGAAACACTACAA ATTAAAGGCGAGCTTTCGTACCAACTTGTTAGCAATATTATTAGACAGCT GTGTGAAGCGCTCAACGATTTGCACAAGCACAATTTCATACACAACGACA TAAAACTCGAAAATGTCTTATATTTCGAAGCACTTGATCGCGTGTATGTT TGCGATTACGGATTGTGCAAACACGAAAACTCACTTAGCGTGCACGACGG CACGTTGGAGTATTTTAGTCCGGAAAAAATTCGACACACAACTATGCACG TTTCGTTTGACTGGTACGCGGCGTGTTAACATACAAGTTGCTAACGTAAT CATGGTCATAGCTGTTTCCTGTGTGAAATTGTTATCCGCTCACAATTCCA CACAACATACGAGCCGGAAGCATAAAGTGTAAAGCCTGGGGTGCCTAATG AGTGAGCTAACTCACATTAATTGCGTTGCGCTCACTGCCCGCTTTCCAGT CGGGAAACCTGTCGTGCCAGCTGCATTAATGAATCGGCCAACGCGCGGGG AGAGGCGGTTTGCGTATTGGGCGCTCTTCCGCTTCCTCGCTCACTGACTC GCTGCGCTCGGTCGTTCGGCTGCGGCGAGCGGTATCAGCTCACTCAAAGG CGGTAATACGGTTATCCACAGAATCAGGGGATAACGCAGGAAAGAACATG TGAGCAAAAGGCCAGCAAAAGGCCAGGAACCGTAAAAAGGCCGCGTTGCT GGCGTTTTTCCATAGGCTCCGCCCCCCTGACGAGCATCACAAAAATCGAC GCTCAAGTCAGAGGTGGCGAAACCCGACAGGACTATAAAGATACCAGGCG TTTCCCCCTGGAAGCTCCCTCGTGCGCTCTCCTGTTCCGACCCTGCCGCT TACCGGATACCTGTCCGCCTTTCTCCCTTCGGGAAGCGTGGCGCTTTCTC ATAGCTCACGCTGTAGGTATCTCAGTTCGGTGTAGGTCGTTCGCTCCAAG CTGGGCTGTGTGCACGAACCCCCCGTTCAGCCCGACCGCTGCGCCTTATC CGGTAACTATCGTCTTGAGTCCAACCCGGTAAGACACGACTTATCGCCAC TGGCAGCAGCCACTGGTAACAGGATTAGCAGAGCGAGGTATGTAGGCGGT GCTACAGAGTTCTTGAAGTGGTGGCCTAACTACGGCTACACTAGAAGGAC AGTATTTGGTATCTGCGCTCTGCTGAAGCCAGTTACCTTCGGAAAAAGAG TTGGTAGCTCTTGATCCGGCAAACAAACCACCGCTGGTAGCGGTGGTTTT TTTGTTTGCAAGCAGCAGATTACGCGCAGAAAAAAAGGATCTCAAGAAGA TCCTTTGATCTTTTCTACGGGGTCTGACGCTCAGTGGAACGAAAACTCAC GTTAAGGGATTTTGGTCATGAGATTATCAAAAAGGATCTTCACCTAGATC CTTTTAAATTAAAAATGAAGTTTTAAATCAATCTAAAGTATATATGAGTA AACTTGGTCTGACAGTTACCAATGCTTAATCAGTGAGGCACCTATCTCAG CGATCTGTCTATTTCGTTCATCCATAGTTGCCTGACTCCCCGTCGTGTAG ATAACTACGATACGGGAGGGCTTACCATCTGGCCCCAGTGCTGCAATGAT ACCGCGAGACCCACGCTCACCGGCTCCAGATTTATCAGCAATAAACCAGC CAGCCGGAAGGGCCGAGCGCAGAAGTGGTCCTGCAACTTTATCCGCCTCC ATCCAGTCTATTAATTGTTGCCGGGAAGCTAGAGTAAGTAGTTCGCCAGT TAATAGTTTGCGCAACGTTGTTGCCATTGCTACAGGCATCGTGGTGTCAC GCTCGTCGTTTGGTATGGCTTCATTCAGCTCCGGTTCCCAACGATCAAGG CGAGTTACATGATCCCCCATGTTGTGCAAAAAAGCGGTTAGCTCCTTCGG TCCTCCGATCGTTGTCAGAAGTAAGTTGGCCGCAGTGTTATCACTCATGG TTATGGCAGCACTGCATAATTCTCTTACTGTCATGCCATCCGTAAGATGC TTTTCTGTGACTGGTGAGTACTCAACCAAGTCATTCTGAGAATAGTGTAT GCGGCGACCGAGTTGCTCTTGCCCGGCGTCAATACGGGATAATACCGCGC CACATAGCAGAACTTTAAAAGTGCTCATCATTGGAAAACGTTCTTCGGGG CGAAAACTCTCAAGGATCTTACCGCTGTTGAGATCCAGTTCGATGTAACC CACTCGTGCACCCAACTGATCTTCAGCATCTTTTACTTTCACCAGCGTTT CTGGGTGAGCAAAAACAGGAAGGCAAAATGCCGCAAAAAAGGGAATAAGG GCGACACGGAAATGTTGAATACTCATACTCTTCCTTTTTCAATATTATTG AAGCATTTATCAGGGTTATTGTCTCATGAGCGGATACATATTTGAATGTA TTTAGAAAAATAAACAAATAGGGGTTCCGCGCACATTTCCCCGAAAAGTG CCACCTGACGTCTAAGAAACCATTATTATCATGACATTAACCTATAAAAA TAGGCGTATCACGAGGCCCTTTCGTCTCGCGCGTTTCGGTGATGACGGTG AAAACCTCTGACACATGCAGCTCCCGGAGACGGTCACAGCTTGTCTGTAA GCGGATGCCGGGAGCAGACAAGCCCGTCAGGGCGCGTCAGCGGGTGTTGG CGGGTGTCGGGGCTGGCTTAACTATGCGGCATCAGAGCAGATTGTACTGA GAGTGCACCATATGCGGTGTGAAATACCGCACAGATGCGTAAGGAGAAAA TACCGCATCAGGCGCCATTCGCCATTCAGGCTGCGCAACTGTTGGGAAGG GCGATCGGTGCGGGCCTCTTCGCTATTACGCCAGCTGGCGAAAGGGGGAT GTGCTGCAAGGCGATTAAGTTGGGTAACGCCAGGGTTTTCCCAGTCACGA CGTTGTAAAACGACGGCCAGTGCC

>pVL1392-Ku70a-HA

AAGCTTTACTCGTAAAGCGAGTTGAAGGATCATATTTAGTTGCGTTTATG AGATAAGATTGAAAGCACGTGTAAAATGTTTCCCGCGCGTTGGCACAACT ATTTACAATGCGGCCAAGTTATAAAAGATTCTAATCTGATATGTTTTAAA ACACCTTTGCGGCCCGAGTTGTTTGCGTACGTGACTAGCGAAGAAGATGT GTGGACCGCAGAACAGATAGTAAAACAAAACCCTAGTATTGGAGCAATAA TCGATTTAACCAACACGTCTAAATATTATGATGGTGTGCATTTTTTGCGG GCGGGCCTGTTATACAAAAAAATTCAAGTACCTGGCCAGACTTTGCCGCC TGAAAGCATAGTTCAAGAATTTATTGACACGGTAAAAGAATTTACAGAAA AGTGTCCCGGCATGTTGGTGGGCGTGCACTGCACACACGGTATTAATCGC ACCGGTTACATGGTGTGCAGATATTTAATGCACACCCTGGGTATTGCGCC GCAGGAAGCCATAGATAGATTCGAAAAAGCCAGAGGTCACAAAATTGAAA GACAAAATTACGTTCAAGATTTATTAATTTAATTAATATTATTTGCATTC TTTAACAAATACTTTATCCTATTTTCAAATTGTTGCGCTTCTTCCAGCGA ACCAAAACTATGCTTCGCTTGCTCCGTTTAGCTTGTAGCCGATCAGTGGC GTTGTTCCAATCGACGGTAGGATTAGGCCGGATATTCTCCACCACAATGT TGGCAACGTTGATGTTACGTTTATGCTTTTGGTTTTCCACGTACGTCTTT TGGCCGGTAATAGCCGTAAACGTAGTGCCGTCGCGCGTCACGCACAACAC CGGATGTTTGCGCTTGTCCGCGGGGTATTGAACCGCGCGATCCGACAAAT CCACCACTTTGGCAACTAAATCGGTGACCTGCGCGTCTTTTTTCTGCATT ATTTCGTCTTTCTTTTGCATGGTTTCCTGGAAGCCGGTGTACATGCGGTT TAGATCAGTCATGACGCGCGTGACCTGCAAATCTTTGGCCTCGATCTGCT TGTCCTTGATGGCAACGATGCGTTCAATAAACTCTTGTTTTTTAACAAGT TCCTCGGTTTTTTGCGCCACCACCGCTTGCAGCGCGTTTGTGTGCTCGGT GAATGTCGCAATCAGCTTAGTCACCAACTGTTTGCTCTCCTCCTCCCGTT GTTTGATCGCGGGATCGTACTTGCCGGTGCAGAGCACTTGAGGAATTACT TCTTCTAAAAGCCATTCTTGTAATTCTATGGCGTAAGGCAATTTGGACTT CATAATCAGCTGAATCACGCCGGATTTAGTAATGAGCACTGTATGCGGCT GCAAATACAGCGGGTCGCCCCTTTTCACGACGCTGTTAGAGGTAGGGCCC CCATTTTGGATGGTCTGCTCAAATAACGATTTGTATTTATTGTCTACATG AACACGTATAGCTTTATCACAAACTGTATATTTTAAACTGTTAGCGACGT CCTTGGCCACGAACCGGACCTGTTGGTCGCGCTCTAGCACGTACCGCAGG TTGAACGTATCTTCTCCAAATTTAAATTCTCCAATTTTAACGCGAGCCAT TTTGATACACGTGTGTCGATTTTGCAACAACTATTGTTTTTTAACGCAAA CTAAACTTATTGTGGTAAGCAATAATTAAATATGGGGGAACATGCGCCGC TACAACACTCGTCGTTATGAACGCAGACGGCGCCGGTCTCGGCGCAAGCG GCTAAAACGTGTTGCGCGTTCAACGCGGCAAACATCGCAAAAGCCAATAG TACAGTTTTGATTTGCATATTAACGGCGATTTTTTAAATTATCTTATTTA ATAAATAGTTATGACGCCTACAACTCCCCGCCCGCGTTGACTCGCTGCAC CTCGAGCAGTTCGTTGACGCCTTCCTCCGTGTGGCCGAACACGTCGAGCG GGTGGTCGATGACCAGCGGCGTGCCGCACGCGACGCACAAGTATCTGTAC ACCGAATGATCGTCGGGCGAAGGCACGTCGGCCTCCAAGTGGCAATATTG GCAAATTCGAAAATATATACAGTTGGGTTGTTTGCGCATATCTATCGTGG CGTTGGGCATGTACGTCCGAACGTTGATTTGCATGCAAGCCGAAATTAAA TCATTGCGATTAGTGCGATTAAAACGTTGTACATCCTCGCTTTTAATCAT GCCGTCGATTAAATCGCGCAATCGAGTCAAGTGATCAAAGTGTGGAATAA TGTTTTCTTTGTATTCCCGAGTCAAGCGCAGCGCGTATTTTAACAAACTA GCCATCTTGTAAGTTAGTTTCATTTAATGCAACTTTATCCAATAATATAT TATGTATCGCACGTCAAGAATTAACAATGCGCCCGTTGTCGCATCTCAAC ACGACTATGATAGAGATCAAATAAAGCGCGAATTAAATAGCTTGCGACGC AACGTGCACGATCTGTGCACGCGTTCCGGCACGAGCTTTGATTGTAATAA GTTTTTACGAAGCGATGACATGACCCCCGTAGTGACAACGATCACGCCCA AAAGAACTGCCGACTACAAAATTACCGAGTATGTCGGTGACGTTAAAACT ATTAAGCCATCCAATCGACCGTTAGTCGAATCAGGACCGCTGGTGCGAGA AGCCGCGAAGTATGGCGAATGCATCGTATAACGTGTGGAGTCCGCTCATT AGAGCGTCATGTTTAGACAAGAAAGCTACATATTTAATTGATCCCGATGA TTTTATTGATAAATTGACCCTAACTCCATACACGGTATTCTACAATGGCG GGGTTTTGGTCAAAATTTCCGGACTGCGATTGTACATGCTGTTAACGGCT CCGCCCACTATTAATGAAATTAAAAATTCCAATTTTAAAAAACGCAGCAA GAGAAACATTTGTATGAAAGAATGCGTAGAAGGAAAGAAAAATGTCGTCG ACATGCTGAACAACAAGATTAATATGCCTCCGTGTATAAAAAAAATATTG AACGATTTGAAAGAAAACAATGTACCGCGCGGCGGTATGTACAGGAAGAG GTTTATACTAAACTGTTACATTGCAAACGTGGTTTCGTGTGCCAAGTGTG AAAACCGATGTTTAATCAAGGCTCTGACGCATTTCTACAACCACGACTCC AAGTGTGTGGGTGAAGTCATGCATCTTTTAATCAAATCCCAAGATGTGTA TAAACCACCAAACTGCCAAAAAATGAAAACTGTCGACAAGCTCTGTCCGT TTGCTGGCAACTGCAAGGGTCTCAATCCTATTTGTAATTATTGAATAATA AAACAATTATAAATGCTAAATTTGTTTTTTATTAACGATACAAACCAAAC GCAACAAGAACATTTGTAGTATTATCTATAATTGAAAACGCGTAGTTATA ATCGCTGAGGTAATATTTAAAATCATTTTCAAATGATTCACAGTTAATTT GCGACAATATAATTTTATTTTCACATAAACTAGACGCCTTGTCGTCTTCT TCTTCGTATTCCTTCTCTTTTTCATTTTTCTCCTCATAAAAATTAACATA GTTATTATCGTATCCATATATGTATCTATCGTATAGAGTAAATTTTTTGT TGTCATAAATATATATGTCTTTTTTAATGGGGTGTATAGTACCGCTGCGC ATAGTTTTTCTGTAATTTACAACAGTGCTATTTTCTGGTAGTTCTTCGGA GTGTGTTGCTTTAATTATTAAATTTATATAATCAATGAATTTGGGATCGT CGGTTTTGTACAATATGTTGCCGGCATAGTACGCAGCTTCTTCTAGTTCA ATTACACCATTTTTTAGCAGCACCGGATTAACATAACTTTCCAAAATGTT GTACGAACCGTTAAACAAAAACAGTTCACCTCCCTTTTCTATACTATTGT CTGCGAGCAGTTGTTTGTTGTTAAAAATAACAGCCATTGTAATGAGACGC ACAAACTAATATCACAAACTGGAAATGTCTATCAATATATAGTTGCTGAT ATCATGGAGATAATTAAAATGATAACCATCTCGCAAATAAATAAGTATTT TACTGTTTTCGTAACAGTTTTGTAATAAAAAAACCTATAAATATTCCGGA TTATTCATACCGTCCCACCATCGGGCGCGGATCAGATCTATGGACTTTGA AGAAGAGCATGCAGGCGGCGATGACGTTGAAGAAATTCAAGACGGCGAGG ACATTTTCGGCGAGGATGCAGATATTAGCGTTCACGACTCCGGGAGTAAG AAGGATGCCGTGATTTTCCTGGTAGACTGTAAGAAAGCGCTTTTCGACAT GGATCAAGACGGACAAGGTACGGTTTTCAGCAAAATCCTCAGTGCCTTCT CGTCCTTTATGAAAGCGAAAATTATTAGCTCTCCTGATGATCGCATTGGT ATGATCTTTTATAACACCAAATCCACCAATAACCAGCTGAAATTTAACAA CATCACCGAAATCTACAAATTAGATGGTCCGTCAGCTGATATCATTAAGA ATTGCCTGAAAATTGAGCAGAACTTTGAAAAGGACTACCAACTGGGCAAT AACGCCCATTTTCACGAATGTCTCTGGCTGTGCAATCACGAGTTCAAAGA ACTGGACAAAAACAAATTTAACATGCGCATCTTCTTATTCACCCCAGACG ATCTGCCGTATTTCAAAGACTTGAACGCTCGCTCTTCTGCCCTGAAATAT GCCAAGCAGCTGAAAGATGCAGATGTGCAGATTGAGCTGTTTCCGTTACC GAGCCAGAATGAATTTAAAATTGCGCGCTTTTACGGCGAGATCATTACCG TAGACCTGGACGAAGTTAACAATGCAGTGCTGGACACCAGCACGAAAATC ATGGATCTGCATCAGCGTATCAAACAGAAAGAGTTTAAAAAACGTGCGCT CAATCGCTTGATCATGGATATTGATGATATCAAAATTGGTCTGAAGATTT ACTGCCTTGTCAACAAAGCCAAGAAACCGTATGGCAAACCACTGGACCGT CGCTATAATCAGCAGCTGAAGAAGAAAGCGCAATTCATTGATGAGGAAAC GGGTCAGGCTTTGTTTCCGCAGCAAATTTCGACCCATCTCATTCTGGGAA ACGAAAAGATCGCAATCCCCAAAGAGTACATGGCGAAAATCAAAGGGTTT GAAAAGCCGGGCATGACTCTGATTGGCTTTAAATCATCAAGCGCGCTGAA AGATTATCACAATTATCGTGCTAGTTATTTTCTGTATCCCGATGATGAAC ATGTCAACGGGAGTAGTCAGTTCTTCGATGCACTGATTCAGCAAATGATC CTGAAGGAGAAAATCGGCATTGTCCGTTTAGTGCCTAAACAAGGGTCGCA AGTTCGCTTTTGTGCGTTGCTGCCGCAAGCAGAACAGTATGACGAGAACC ACTTTCAGACTCCGCCAGGTTTGCATCTGATTTTCCTGCCGTATGCGGAT GATATCCGCGGCTTGTCAACTGTGAAACAAGAAGGCGCCGAAATCACGCG CCAAACCCTGAATGCTGCGAAAATTCTCGTGAACGCGCTGACAATCCAGG ACTTCGATTGCTCTAATTTTGAAGATCCGAGCATTCAGAAATTTTACACG TACTTACAGGGGCTTGCCCTTCAGGAACAGAACATTGAGGAACCCGAAGA TTTGCTTCAGCCGGATTTTAAAGGTATGGAGAAATACCGCGATATTGTGA ATCTGTTCATGAGCAACGTATCCTTAGAATGCAGCAATATGCCATCCCGC TCGAAAGGGCAAGGCGGTGGTCGTGGCCGTGGTCGTGGTCGCGGTCGGGG TAAACAGGAAGAATCGGAAAGTGATGACTGTTCGAAAGTCAAAGGACGCG GACGTGGTAGCACACAGAAACAGAAAATCGAAGAAGATGACTCTTTAGAA GGCGAAGAAATTTACCAGCCTGTGAAAAAGCGCGGCCGTGGCCGGTCTAG AGCATACCCATACGATGTTCCAGATTACGCTTGAGATCCTTTCCTGGGAC CCGGCAAGAACCAAAAACTCACTCTCTTCAAGGAAATCCGTAATGTTAAA CCCGACACGATGAAGCTTGTCGTTGGATGGAAAGGAAAAGAGTTCTACAG GGAAACTTGGACCCGCTTCATGGAAGACAGCTTCCCCATTGTTAACGACC AAGAAGTGATGGATGTTTTCCTTGTTGTCAACATGCGTCCCACTAGACCC AACCGTTGTTACAAATTCCTGGCCCAACACGCTCTGCGTTGCGACCCCGA CTATGTACCTCATGACGTGATTAGGATCGTCGAGCCTTCATGGGTGGGCA GCAACAACGAGTACCGCATCAGCCTGGCTAAGAAGGGCGGCGGCTGCCCA ATAATGAACCTTCACTCTGAGTACACCAACTCGTTCGAACAGTTCATCGA TCGTGTCATCTGGGAGAACTTCTACAAGCCCATCGTTTACATCGGTACCG ACTCTGCTGAAGAGGAGGAAATTCTCCTTGAAGTTTCCCTGGTGTTCAAA GTAAAGGAGTTTGCACCAGACGCACCTCTGTTCACTGGTCCGGCGTATTA AAACACGATACATTGTTATTAGTACATTTATTAAGCGCTAGATTCTGTGC GTTGTTGATTTACAGACAATTGTTGTACGTATTTTAATAATTCATTAAAT TTATAATCTTTAGGGTGGTATGTTAGAGCGAAAATCAAATGATTTTCAGC GTCTTTATATCTGAATTTAAATATTAAATCCTCAATAGATTTGTAAAATA GGTTTCGATTAGTTTCAAACAAGGGTTGTTTTTCCGAACCGATGGCTGGA CTATCTAATGGATTTTCGCTCAACGCCACAAAACTTGCCAAATCTTGTAG CAGCAATCTAGCTTTGTCGATATTCGTTTGTGTTTTGTTTTGTAATAAAG GTTCGACGTCGTTCAAAATATTATGCGCTTTTGTATTTCTTTCATCACTG TCGTTAGTGTACAATTGACTCGACGTAAACACGTTAAATAAAGCTTGGAC ATATTTAACATCGGGCGTGTTAGCTTTATTAGGCCGATTATCGTCGTCGT CCCAACCCTCGTCGTTAGAAGTTGCTTCCGAAGACGATTTTGCCATAGCC ACACGACGCCTATTAATTGTGTCGGCTAACACGTCCGCGATCAAATTTGT AGTTGAGCTTTTTGGAATTATTTCTGATTGCGGGCGTTTTTGGGCGGGTT TCAATCTAACTGTGCCCGATTTTAATTCAGACAACACGTTAGAAAGCGAT GGTGCAGGCGGTGGTAACATTTCAGACGGCAAATCTACTAATGGCGGCGG TGGTGGAGCTGATGATAAATCTACCATCGGTGGAGGCGCAGGCGGGGCTG GCGGCGGAGGCGGAGGCGGAGGTGGTGGCGGTGATGCAGACGGCGGTTTA GGCTCAAATGTCTCTTTAGGCAACACAGTCGGCACCTCAACTATTGTACT GGTTTCGGGCGCCGTTTTTGGTTTGACCGGTCTGAGACGAGTGCGATTTT TTTCGTTTCTAATAGCTTCCAACAATTGTTGTCTGTCGTCTAAAGGTGCA GCGGGTTGAGGTTCCGTCGGCATTGGTGGAGCGGGCGGCAATTCAGACAT CGATGGTGGTGGTGGTGGTGGAGGCGCTGGAATGTTAGGCACGGGAGAAG GTGGTGGCGGCGGTGCCGCCGGTATAATTTGTTCTGGTTTAGTTTGTTCG CGCACGATTGTGGGCACCGGCGCAGGCGCCGCTGGCTGCACAACGGAAGG TCGTCTGCTTCGAGGCAGCGCTTGGGGTGGTGGCAATTCAATATTATAAT TGGAATACAAATCGTAAAAATCTGCTATAAGCATTGTAATTTCGCTATCG TTTACCGTGCCGATATTTAACAACCGCTCAATGTAAGCAATTGTATTGTA AAGAGATTGTCTCAAGCTCGCCGCACGCCGATAACAAGCCTTTTCATTTT TACTACAGCATTGTAGTGGCGAGACACTTCGCTGTCGTCGACGTACATGT ATGCTTTGTTGTCAAAAACGTCGTTGGCAAGCTTTAAAATATTTAAAAGA ACATCTCTGTTCAGCACCACTGTGTTGTCGTAAATGTTGTTTTTGATAAT TTGCGCTTCCGCAGTATCGACACGTTCAAAAAATTGATGCGCATCAATTT TGTTGTTCCTATTATTGAATAAATAAGATTGTACAGATTCATATCTACGA TTCGTCATGGCCACCACAAATGCTACGCTGCAAACGCTGGTACAATTTTA CGAAAACTGCAAAAACGTCAAAACTCGGTATAAAATAATCAACGGGCGCT TTGGCAAAATATCTATTTTATCGCACAAGCCCACTAGCAAATTGTATTTG CAGAAAACAATTTCGGCGCACAATTTTAACGCTGACGAAATAAAAGTTCA CCAGTTAATGAGCGACCACCCAAATTTTATAAAAATCTATTTTAATCACG GTTCCATCAACAACCAAGTGATCGTGATGGACTACATTGACTGTCCCGAT TTATTTGAAACACTACAAATTAAAGGCGAGCTTTCGTACCAACTTGTTAG CAATATTATTAGACAGCTGTGTGAAGCGCTCAACGATTTGCACAAGCACA ATTTCATACACAACGACATAAAACTCGAAAATGTCTTATATTTCGAAGCA CTTGATCGCGTGTATGTTTGCGATTACGGATTGTGCAAACACGAAAACTC ACTTAGCGTGCACGACGGCACGTTGGAGTATTTTAGTCCGGAAAAAATTC GACACACAACTATGCACGTTTCGTTTGACTGGTACGCGGCGTGTTAACAT ACAAGTTGCTAACGTAATCATGGTCATAGCTGTTTCCTGTGTGAAATTGT TATCCGCTCACAATTCCACACAACATACGAGCCGGAAGCATAAAGTGTAA AGCCTGGGGTGCCTAATGAGTGAGCTAACTCACATTAATTGCGTTGCGCT CACTGCCCGCTTTCCAGTCGGGAAACCTGTCGTGCCAGCTGCATTAATGA ATCGGCCAACGCGCGGGGAGAGGCGGTTTGCGTATTGGGCGCTCTTCCGC TTCCTCGCTCACTGACTCGCTGCGCTCGGTCGTTCGGCTGCGGCGAGCGG TATCAGCTCACTCAAAGGCGGTAATACGGTTATCCACAGAATCAGGGGAT AACGCAGGAAAGAACATGTGAGCAAAAGGCCAGCAAAAGGCCAGGAACCG TAAAAAGGCCGCGTTGCTGGCGTTTTTCCATAGGCTCCGCCCCCCTGACG AGCATCACAAAAATCGACGCTCAAGTCAGAGGTGGCGAAACCCGACAGGA CTATAAAGATACCAGGCGTTTCCCCCTGGAAGCTCCCTCGTGCGCTCTCC TGTTCCGACCCTGCCGCTTACCGGATACCTGTCCGCCTTTCTCCCTTCGG GAAGCGTGGCGCTTTCTCATAGCTCACGCTGTAGGTATCTCAGTTCGGTG TAGGTCGTTCGCTCCAAGCTGGGCTGTGTGCACGAACCCCCCGTTCAGCC CGACCGCTGCGCCTTATCCGGTAACTATCGTCTTGAGTCCAACCCGGTAA GACACGACTTATCGCCACTGGCAGCAGCCACTGGTAACAGGATTAGCAGA GCGAGGTATGTAGGCGGTGCTACAGAGTTCTTGAAGTGGTGGCCTAACTA CGGCTACACTAGAAGGACAGTATTTGGTATCTGCGCTCTGCTGAAGCCAG TTACCTTCGGAAAAAGAGTTGGTAGCTCTTGATCCGGCAAACAAACCACC GCTGGTAGCGGTGGTTTTTTTGTTTGCAAGCAGCAGATTACGCGCAGAAA AAAAGGATCTCAAGAAGATCCTTTGATCTTTTCTACGGGGTCTGACGCTC AGTGGAACGAAAACTCACGTTAAGGGATTTTGGTCATGAGATTATCAAAA AGGATCTTCACCTAGATCCTTTTAAATTAAAAATGAAGTTTTAAATCAAT CTAAAGTATATATGAGTAAACTTGGTCTGACAGTTACCAATGCTTAATCA GTGAGGCACCTATCTCAGCGATCTGTCTATTTCGTTCATCCATAGTTGCC TGACTCCCCGTCGTGTAGATAACTACGATACGGGAGGGCTTACCATCTGG CCCCAGTGCTGCAATGATACCGCGAGACCCACGCTCACCGGCTCCAGATT TATCAGCAATAAACCAGCCAGCCGGAAGGGCCGAGCGCAGAAGTGGTCCT GCAACTTTATCCGCCTCCATCCAGTCTATTAATTGTTGCCGGGAAGCTAG AGTAAGTAGTTCGCCAGTTAATAGTTTGCGCAACGTTGTTGCCATTGCTA CAGGCATCGTGGTGTCACGCTCGTCGTTTGGTATGGCTTCATTCAGCTCC GGTTCCCAACGATCAAGGCGAGTTACATGATCCCCCATGTTGTGCAAAAA AGCGGTTAGCTCCTTCGGTCCTCCGATCGTTGTCAGAAGTAAGTTGGCCG CAGTGTTATCACTCATGGTTATGGCAGCACTGCATAATTCTCTTACTGTC ATGCCATCCGTAAGATGCTTTTCTGTGACTGGTGAGTACTCAACCAAGTC ATTCTGAGAATAGTGTATGCGGCGACCGAGTTGCTCTTGCCCGGCGTCAA TACGGGATAATACCGCGCCACATAGCAGAACTTTAAAAGTGCTCATCATT GGAAAACGTTCTTCGGGGCGAAAACTCTCAAGGATCTTACCGCTGTTGAG ATCCAGTTCGATGTAACCCACTCGTGCACCCAACTGATCTTCAGCATCTT TTACTTTCACCAGCGTTTCTGGGTGAGCAAAAACAGGAAGGCAAAATGCC GCAAAAAAGGGAATAAGGGCGACACGGAAATGTTGAATACTCATACTCTT CCTTTTTCAATATTATTGAAGCATTTATCAGGGTTATTGTCTCATGAGCG GATACATATTTGAATGTATTTAGAAAAATAAACAAATAGGGGTTCCGCGC ACATTTCCCCGAAAAGTGCCACCTGACGTCTAAGAAACCATTATTATCAT GACATTAACCTATAAAAATAGGCGTATCACGAGGCCCTTTCGTCTCGCGC GTTTCGGTGATGACGGTGAAAACCTCTGACACATGCAGCTCCCGGAGACG GTCACAGCTTGTCTGTAAGCGGATGCCGGGAGCAGACAAGCCCGTCAGGG CGCGTCAGCGGGTGTTGGCGGGTGTCGGGGCTGGCTTAACTATGCGGCAT CAGAGCAGATTGTACTGAGAGTGCACCATATGCGGTGTGAAATACCGCAC AGATGCGTAAGGAGAAAATACCGCATCAGGCGCCATTCGCCATTCAGGCT GCGCAACTGTTGGGAAGGGCGATCGGTGCGGGCCTCTTCGCTATTACGCC AGCTGGCGAAAGGGGGATGTGCTGCAAGGCGATTAAGTTGGGTAACGCCA GGGTTTTCCCAGTCACGACGTTGTAAAACGACGGCCAGTGCC

>pVL1392-HA-Ku80c

AAGCTTTACTCGTAAAGCGAGTTGAAGGATCATATTTAGTTGCGTTTATG AGATAAGATTGAAAGCACGTGTAAAATGTTTCCCGCGCGTTGGCACAACT ATTTACAATGCGGCCAAGTTATAAAAGATTCTAATCTGATATGTTTTAAA ACACCTTTGCGGCCCGAGTTGTTTGCGTACGTGACTAGCGAAGAAGATGT GTGGACCGCAGAACAGATAGTAAAACAAAACCCTAGTATTGGAGCAATAA TCGATTTAACCAACACGTCTAAATATTATGATGGTGTGCATTTTTTGCGG GCGGGCCTGTTATACAAAAAAATTCAAGTACCTGGCCAGACTTTGCCGCC TGAAAGCATAGTTCAAGAATTTATTGACACGGTAAAAGAATTTACAGAAA AGTGTCCCGGCATGTTGGTGGGCGTGCACTGCACACACGGTATTAATCGC ACCGGTTACATGGTGTGCAGATATTTAATGCACACCCTGGGTATTGCGCC GCAGGAAGCCATAGATAGATTCGAAAAAGCCAGAGGTCACAAAATTGAAA GACAAAATTACGTTCAAGATTTATTAATTTAATTAATATTATTTGCATTC TTTAACAAATACTTTATCCTATTTTCAAATTGTTGCGCTTCTTCCAGCGA ACCAAAACTATGCTTCGCTTGCTCCGTTTAGCTTGTAGCCGATCAGTGGC GTTGTTCCAATCGACGGTAGGATTAGGCCGGATATTCTCCACCACAATGT TGGCAACGTTGATGTTACGTTTATGCTTTTGGTTTTCCACGTACGTCTTT TGGCCGGTAATAGCCGTAAACGTAGTGCCGTCGCGCGTCACGCACAACAC CGGATGTTTGCGCTTGTCCGCGGGGTATTGAACCGCGCGATCCGACAAAT CCACCACTTTGGCAACTAAATCGGTGACCTGCGCGTCTTTTTTCTGCATT ATTTCGTCTTTCTTTTGCATGGTTTCCTGGAAGCCGGTGTACATGCGGTT TAGATCAGTCATGACGCGCGTGACCTGCAAATCTTTGGCCTCGATCTGCT TGTCCTTGATGGCAACGATGCGTTCAATAAACTCTTGTTTTTTAACAAGT TCCTCGGTTTTTTGCGCCACCACCGCTTGCAGCGCGTTTGTGTGCTCGGT GAATGTCGCAATCAGCTTAGTCACCAACTGTTTGCTCTCCTCCTCCCGTT GTTTGATCGCGGGATCGTACTTGCCGGTGCAGAGCACTTGAGGAATTACT TCTTCTAAAAGCCATTCTTGTAATTCTATGGCGTAAGGCAATTTGGACTT CATAATCAGCTGAATCACGCCGGATTTAGTAATGAGCACTGTATGCGGCT GCAAATACAGCGGGTCGCCCCTTTTCACGACGCTGTTAGAGGTAGGGCCC CCATTTTGGATGGTCTGCTCAAATAACGATTTGTATTTATTGTCTACATG AACACGTATAGCTTTATCACAAACTGTATATTTTAAACTGTTAGCGACGT CCTTGGCCACGAACCGGACCTGTTGGTCGCGCTCTAGCACGTACCGCAGG TTGAACGTATCTTCTCCAAATTTAAATTCTCCAATTTTAACGCGAGCCAT TTTGATACACGTGTGTCGATTTTGCAACAACTATTGTTTTTTAACGCAAA CTAAACTTATTGTGGTAAGCAATAATTAAATATGGGGGAACATGCGCCGC TACAACACTCGTCGTTATGAACGCAGACGGCGCCGGTCTCGGCGCAAGCG GCTAAAACGTGTTGCGCGTTCAACGCGGCAAACATCGCAAAAGCCAATAG TACAGTTTTGATTTGCATATTAACGGCGATTTTTTAAATTATCTTATTTA ATAAATAGTTATGACGCCTACAACTCCCCGCCCGCGTTGACTCGCTGCAC CTCGAGCAGTTCGTTGACGCCTTCCTCCGTGTGGCCGAACACGTCGAGCG GGTGGTCGATGACCAGCGGCGTGCCGCACGCGACGCACAAGTATCTGTAC ACCGAATGATCGTCGGGCGAAGGCACGTCGGCCTCCAAGTGGCAATATTG GCAAATTCGAAAATATATACAGTTGGGTTGTTTGCGCATATCTATCGTGG CGTTGGGCATGTACGTCCGAACGTTGATTTGCATGCAAGCCGAAATTAAA TCATTGCGATTAGTGCGATTAAAACGTTGTACATCCTCGCTTTTAATCAT GCCGTCGATTAAATCGCGCAATCGAGTCAAGTGATCAAAGTGTGGAATAA TGTTTTCTTTGTATTCCCGAGTCAAGCGCAGCGCGTATTTTAACAAACTA GCCATCTTGTAAGTTAGTTTCATTTAATGCAACTTTATCCAATAATATAT TATGTATCGCACGTCAAGAATTAACAATGCGCCCGTTGTCGCATCTCAAC ACGACTATGATAGAGATCAAATAAAGCGCGAATTAAATAGCTTGCGACGC AACGTGCACGATCTGTGCACGCGTTCCGGCACGAGCTTTGATTGTAATAA GTTTTTACGAAGCGATGACATGACCCCCGTAGTGACAACGATCACGCCCA AAAGAACTGCCGACTACAAAATTACCGAGTATGTCGGTGACGTTAAAACT ATTAAGCCATCCAATCGACCGTTAGTCGAATCAGGACCGCTGGTGCGAGA AGCCGCGAAGTATGGCGAATGCATCGTATAACGTGTGGAGTCCGCTCATT AGAGCGTCATGTTTAGACAAGAAAGCTACATATTTAATTGATCCCGATGA TTTTATTGATAAATTGACCCTAACTCCATACACGGTATTCTACAATGGCG GGGTTTTGGTCAAAATTTCCGGACTGCGATTGTACATGCTGTTAACGGCT CCGCCCACTATTAATGAAATTAAAAATTCCAATTTTAAAAAACGCAGCAA GAGAAACATTTGTATGAAAGAATGCGTAGAAGGAAAGAAAAATGTCGTCG ACATGCTGAACAACAAGATTAATATGCCTCCGTGTATAAAAAAAATATTG AACGATTTGAAAGAAAACAATGTACCGCGCGGCGGTATGTACAGGAAGAG GTTTATACTAAACTGTTACATTGCAAACGTGGTTTCGTGTGCCAAGTGTG AAAACCGATGTTTAATCAAGGCTCTGACGCATTTCTACAACCACGACTCC AAGTGTGTGGGTGAAGTCATGCATCTTTTAATCAAATCCCAAGATGTGTA TAAACCACCAAACTGCCAAAAAATGAAAACTGTCGACAAGCTCTGTCCGT TTGCTGGCAACTGCAAGGGTCTCAATCCTATTTGTAATTATTGAATAATA AAACAATTATAAATGCTAAATTTGTTTTTTATTAACGATACAAACCAAAC GCAACAAGAACATTTGTAGTATTATCTATAATTGAAAACGCGTAGTTATA ATCGCTGAGGTAATATTTAAAATCATTTTCAAATGATTCACAGTTAATTT GCGACAATATAATTTTATTTTCACATAAACTAGACGCCTTGTCGTCTTCT TCTTCGTATTCCTTCTCTTTTTCATTTTTCTCCTCATAAAAATTAACATA GTTATTATCGTATCCATATATGTATCTATCGTATAGAGTAAATTTTTTGT TGTCATAAATATATATGTCTTTTTTAATGGGGTGTATAGTACCGCTGCGC ATAGTTTTTCTGTAATTTACAACAGTGCTATTTTCTGGTAGTTCTTCGGA GTGTGTTGCTTTAATTATTAAATTTATATAATCAATGAATTTGGGATCGT CGGTTTTGTACAATATGTTGCCGGCATAGTACGCAGCTTCTTCTAGTTCA ATTACACCATTTTTTAGCAGCACCGGATTAACATAACTTTCCAAAATGTT GTACGAACCGTTAAACAAAAACAGTTCACCTCCCTTTTCTATACTATTGT CTGCGAGCAGTTGTTTGTTGTTAAAAATAACAGCCATTGTAATGAGACGC ACAAACTAATATCACAAACTGGAAATGTCTATCAATATATAGTTGCTGAT ATCATGGAGATAATTAAAATGATAACCATCTCGCAAATAAATAAGTATTT TACTGTTTTCGTAACAGTTTTGTAATAAAAAAACCTATAAATATTCCGGA TTATTCATACCGTCCCACCATCGGGCGCGGATCAGATCTATGTACCCATA CGATGTTCCTGACTATGCTGGATCAATGTCGGGTAAAGAAGCGACCCTGA TCCTGCTGGATGTAGGCGCGAGCATGTATGGCCAGTATCAACAGGGTGGA AGCAAGAAACTGAGCCGCTTAGAGCTGGCTGTGGATTGCCTGGGCCTGAT GATCCAACAGAAAATTTTCAACTACAAAAATCATGAAGTTGGGCTGATCC TGTTCGGCACGGAAGAAGCGCCGGATGGGAAAACCCTGTACATTCAGGAT CTCTCGATTCCGGATCTGGACTTCTTCCGCAACATTAGTGATCTGCCAAA TCACGATGTCGGACAGCAAGTGGGTGGCGACATTTTCGACGCATTAGACA AAGCCGTTCATGCCTTAGACGACCATGCTAAAACCAAGAAAATGGAGAAG AAAATCTTCATTCTTACCGCCGGTTGTGGTCAGACCGACTATAGCGAGAA GCAGATTACCAAACTTATCAAAATGATTGAGAAAGTGGATGTTAAAATCA ACTTTATCGCCCTGGACTTTATGAACGACTATAACGGTGATATGGATGAC CCGGAAAAGCCTGAAGAATTTGAAGCACTCAATAATCGCATGTTAACTGC GTCATACCAGTGTCAGGAGCAGAGTATCAACTCGCGTTATGTGTTTCTGA TGGTTCAAGAATTGCGCAATAACATGCGCATCTTTCCGGCGAACGTGGCC TTTGAACTGTACTCTCAGTTTCACACACGCAGTCTGCAAGCACGCGCGTC TTTTCGCGGCGATTTCCAAATTAATGACGAAATTAGTGTTCAGGTTCTGA TCTATAAACGGTGCTTCGAAGAACGGCTGCCGACGTTACGTAAACACAGC ACACTGGGAGAATTTCAGACGGATACTAACAAAAACCATGTCCGCAACGA TCTGATCTATTATAACCCGGAGGATCCAAATATGACTCCCATCGAGAAAG ACAATATCATCCGTGGTTATCAGTATGGGCGTAATTTGGTTCCCGTGGAT CAAATTATGGAAGATAAAATGAAATATCAGTGTCCGCGTCAATTCCAGCT GCTGGGCTTTGTGGACCGCTCTCATATTCCGCGTTACTATTATACCTCCA CGGTCGACATGGTCATTGCTGTCGAGAACCAGAAGCAACAGAAAGCGTTG GCAGCACTTGTAATTGCGCTGATCGCGACTCGCAAAGTAGCACTGGCTCG TTTCGTGGGCCGTGAGAAAACGGCGCCAAAATTGATTATGCTGTTGCCTC ACAAGTCCAAGAATTCACAGTGCTTTTGGATGATTAGTCTGCCAACGACT GAAGATATTCGCCATTTCCAATTTGCAGCTCTTAAGCGGTCAACACCGCC ACAACAGATGGCGGTGAGCGCTATGATCGATTGCATGGATCTGGAGAAAA TGCCGACCGAAGATGGGCAATTTGAAGAATTACTGAAAATGAAATACGTG GCCAATCCTACCCGTCAGTACTTTCAACAGGTCGTTATGCACAAAGCCAT TACCCGCTCGGATGTACTCCCGCCCATTAGCCCCTTAATTCTGGAATACC TTCATCCGGAGAAACGTGTCTATGATTACGCGAAAGAAGCCTTACAGAAG GTGAAAGCGGCATTCAAATTCAAAATTAACGAAATTAAGAAACAGGGCGA TAAGAAAGTGTTTTGGAAACAGTTGTTCGAAGATCAGTCCACCGAACAGA TTCAGCAGCAAGTCGAGGACGAAGTGGTTGAGATCAACCAAGAAGAAGAA GAAATGGTGAACATGTTTGCCAAGCAGAAATTAGGCTTTAACGATGACAT CGTGAAAGAGATCGGTACGGTTGATCCGACATCTGACTTTCGCAAAATGA TTACGGAGAAACGCGTTGATCTTGTAGATACCGCGCTTCAGCAGATTCAG AAAGTGATCATTCAATTCGTTGATCAGTCACTGAAAGGTAGCTTTTACCC TAAAGCCCTCGAGTGCCTCAAAGAGATGCGTAAGGCCTGTATTACCGAAG ATGAAGCTCCGGTATTTAACAAATATCTGCATGTCCTGAAAGAAAAATAC TCTCAACTCGTTTTCTGGGCACAAATTGTGCAGCAGGGCATTACCCTCAT TTCCAATATCGAAAATCAGAAATCCCACGTGAGCGTAGACGAAGCGCAAG AATTTCTGAATAAGGAGGATATCTCGCATAAACAACTGGTCGATCAGTTG CAGCACGAAGAAGAAGATTTGCTGGCCGAAATTGACTGATCTAGAAGGTA CCCGGGATCCTTTCCTGGGACCCGGCAAGAACCAAAAACTCACTCTCTTC AAGGAAATCCGTAATGTTAAACCCGACACGATGAAGCTTGTCGTTGGATG GAAAGGAAAAGAGTTCTACAGGGAAACTTGGACCCGCTTCATGGAAGACA GCTTCCCCATTGTTAACGACCAAGAAGTGATGGATGTTTTCCTTGTTGTC AACATGCGTCCCACTAGACCCAACCGTTGTTACAAATTCCTGGCCCAACA CGCTCTGCGTTGCGACCCCGACTATGTACCTCATGACGTGATTAGGATCG TCGAGCCTTCATGGGTGGGCAGCAACAACGAGTACCGCATCAGCCTGGCT AAGAAGGGCGGCGGCTGCCCAATAATGAACCTTCACTCTGAGTACACCAA CTCGTTCGAACAGTTCATCGATCGTGTCATCTGGGAGAACTTCTACAAGC CCATCGTTTACATCGGTACCGACTCTGCTGAAGAGGAGGAAATTCTCCTT GAAGTTTCCCTGGTGTTCAAAGTAAAGGAGTTTGCACCAGACGCACCTCT GTTCACTGGTCCGGCGTATTAAAACACGATACATTGTTATTAGTACATTT ATTAAGCGCTAGATTCTGTGCGTTGTTGATTTACAGACAATTGTTGTACG TATTTTAATAATTCATTAAATTTATAATCTTTAGGGTGGTATGTTAGAGC GAAAATCAAATGATTTTCAGCGTCTTTATATCTGAATTTAAATATTAAAT CCTCAATAGATTTGTAAAATAGGTTTCGATTAGTTTCAAACAAGGGTTGT TTTTCCGAACCGATGGCTGGACTATCTAATGGATTTTCGCTCAACGCCAC AAAACTTGCCAAATCTTGTAGCAGCAATCTAGCTTTGTCGATATTCGTTT GTGTTTTGTTTTGTAATAAAGGTTCGACGTCGTTCAAAATATTATGCGCT TTTGTATTTCTTTCATCACTGTCGTTAGTGTACAATTGACTCGACGTAAA CACGTTAAATAAAGCTTGGACATATTTAACATCGGGCGTGTTAGCTTTAT TAGGCCGATTATCGTCGTCGTCCCAACCCTCGTCGTTAGAAGTTGCTTCC GAAGACGATTTTGCCATAGCCACACGACGCCTATTAATTGTGTCGGCTAA CACGTCCGCGATCAAATTTGTAGTTGAGCTTTTTGGAATTATTTCTGATT GCGGGCGTTTTTGGGCGGGTTTCAATCTAACTGTGCCCGATTTTAATTCA GACAACACGTTAGAAAGCGATGGTGCAGGCGGTGGTAACATTTCAGACGG CAAATCTACTAATGGCGGCGGTGGTGGAGCTGATGATAAATCTACCATCG GTGGAGGCGCAGGCGGGGCTGGCGGCGGAGGCGGAGGCGGAGGTGGTGGC GGTGATGCAGACGGCGGTTTAGGCTCAAATGTCTCTTTAGGCAACACAGT CGGCACCTCAACTATTGTACTGGTTTCGGGCGCCGTTTTTGGTTTGACCG GTCTGAGACGAGTGCGATTTTTTTCGTTTCTAATAGCTTCCAACAATTGT TGTCTGTCGTCTAAAGGTGCAGCGGGTTGAGGTTCCGTCGGCATTGGTGG AGCGGGCGGCAATTCAGACATCGATGGTGGTGGTGGTGGTGGAGGCGCTG GAATGTTAGGCACGGGAGAAGGTGGTGGCGGCGGTGCCGCCGGTATAATT TGTTCTGGTTTAGTTTGTTCGCGCACGATTGTGGGCACCGGCGCAGGCGC CGCTGGCTGCACAACGGAAGGTCGTCTGCTTCGAGGCAGCGCTTGGGGTG GTGGCAATTCAATATTATAATTGGAATACAAATCGTAAAAATCTGCTATA AGCATTGTAATTTCGCTATCGTTTACCGTGCCGATATTTAACAACCGCTC AATGTAAGCAATTGTATTGTAAAGAGATTGTCTCAAGCTCGCCGCACGCC GATAACAAGCCTTTTCATTTTTACTACAGCATTGTAGTGGCGAGACACTT CGCTGTCGTCGACGTACATGTATGCTTTGTTGTCAAAAACGTCGTTGGCA AGCTTTAAAATATTTAAAAGAACATCTCTGTTCAGCACCACTGTGTTGTC GTAAATGTTGTTTTTGATAATTTGCGCTTCCGCAGTATCGACACGTTCAA AAAATTGATGCGCATCAATTTTGTTGTTCCTATTATTGAATAAATAAGAT TGTACAGATTCATATCTACGATTCGTCATGGCCACCACAAATGCTACGCT GCAAACGCTGGTACAATTTTACGAAAACTGCAAAAACGTCAAAACTCGGT ATAAAATAATCAACGGGCGCTTTGGCAAAATATCTATTTTATCGCACAAG CCCACTAGCAAATTGTATTTGCAGAAAACAATTTCGGCGCACAATTTTAA CGCTGACGAAATAAAAGTTCACCAGTTAATGAGCGACCACCCAAATTTTA TAAAAATCTATTTTAATCACGGTTCCATCAACAACCAAGTGATCGTGATG GACTACATTGACTGTCCCGATTTATTTGAAACACTACAAATTAAAGGCGA GCTTTCGTACCAACTTGTTAGCAATATTATTAGACAGCTGTGTGAAGCGC TCAACGATTTGCACAAGCACAATTTCATACACAACGACATAAAACTCGAA AATGTCTTATATTTCGAAGCACTTGATCGCGTGTATGTTTGCGATTACGG ATTGTGCAAACACGAAAACTCACTTAGCGTGCACGACGGCACGTTGGAGT ATTTTAGTCCGGAAAAAATTCGACACACAACTATGCACGTTTCGTTTGAC TGGTACGCGGCGTGTTAACATACAAGTTGCTAACGTAATCATGGTCATAG CTGTTTCCTGTGTGAAATTGTTATCCGCTCACAATTCCACACAACATACG AGCCGGAAGCATAAAGTGTAAAGCCTGGGGTGCCTAATGAGTGAGCTAAC TCACATTAATTGCGTTGCGCTCACTGCCCGCTTTCCAGTCGGGAAACCTG TCGTGCCAGCTGCATTAATGAATCGGCCAACGCGCGGGGAGAGGCGGTTT GCGTATTGGGCGCTCTTCCGCTTCCTCGCTCACTGACTCGCTGCGCTCGG TCGTTCGGCTGCGGCGAGCGGTATCAGCTCACTCAAAGGCGGTAATACGG TTATCCACAGAATCAGGGGATAACGCAGGAAAGAACATGTGAGCAAAAGG CCAGCAAAAGGCCAGGAACCGTAAAAAGGCCGCGTTGCTGGCGTTTTTCC ATAGGCTCCGCCCCCCTGACGAGCATCACAAAAATCGACGCTCAAGTCAG AGGTGGCGAAACCCGACAGGACTATAAAGATACCAGGCGTTTCCCCCTGG AAGCTCCCTCGTGCGCTCTCCTGTTCCGACCCTGCCGCTTACCGGATACC TGTCCGCCTTTCTCCCTTCGGGAAGCGTGGCGCTTTCTCATAGCTCACGC TGTAGGTATCTCAGTTCGGTGTAGGTCGTTCGCTCCAAGCTGGGCTGTGT GCACGAACCCCCCGTTCAGCCCGACCGCTGCGCCTTATCCGGTAACTATC GTCTTGAGTCCAACCCGGTAAGACACGACTTATCGCCACTGGCAGCAGCC ACTGGTAACAGGATTAGCAGAGCGAGGTATGTAGGCGGTGCTACAGAGTT CTTGAAGTGGTGGCCTAACTACGGCTACACTAGAAGGACAGTATTTGGTA TCTGCGCTCTGCTGAAGCCAGTTACCTTCGGAAAAAGAGTTGGTAGCTCT TGATCCGGCAAACAAACCACCGCTGGTAGCGGTGGTTTTTTTGTTTGCAA GCAGCAGATTACGCGCAGAAAAAAAGGATCTCAAGAAGATCCTTTGATCT TTTCTACGGGGTCTGACGCTCAGTGGAACGAAAACTCACGTTAAGGGATT TTGGTCATGAGATTATCAAAAAGGATCTTCACCTAGATCCTTTTAAATTA AAAATGAAGTTTTAAATCAATCTAAAGTATATATGAGTAAACTTGGTCTG ACAGTTACCAATGCTTAATCAGTGAGGCACCTATCTCAGCGATCTGTCTA TTTCGTTCATCCATAGTTGCCTGACTCCCCGTCGTGTAGATAACTACGAT ACGGGAGGGCTTACCATCTGGCCCCAGTGCTGCAATGATACCGCGAGACC CACGCTCACCGGCTCCAGATTTATCAGCAATAAACCAGCCAGCCGGAAGG GCCGAGCGCAGAAGTGGTCCTGCAACTTTATCCGCCTCCATCCAGTCTAT TAATTGTTGCCGGGAAGCTAGAGTAAGTAGTTCGCCAGTTAATAGTTTGC GCAACGTTGTTGCCATTGCTACAGGCATCGTGGTGTCACGCTCGTCGTTT GGTATGGCTTCATTCAGCTCCGGTTCCCAACGATCAAGGCGAGTTACATG ATCCCCCATGTTGTGCAAAAAAGCGGTTAGCTCCTTCGGTCCTCCGATCG TTGTCAGAAGTAAGTTGGCCGCAGTGTTATCACTCATGGTTATGGCAGCA CTGCATAATTCTCTTACTGTCATGCCATCCGTAAGATGCTTTTCTGTGAC TGGTGAGTACTCAACCAAGTCATTCTGAGAATAGTGTATGCGGCGACCGA GTTGCTCTTGCCCGGCGTCAATACGGGATAATACCGCGCCACATAGCAGA ACTTTAAAAGTGCTCATCATTGGAAAACGTTCTTCGGGGCGAAAACTCTC AAGGATCTTACCGCTGTTGAGATCCAGTTCGATGTAACCCACTCGTGCAC CCAACTGATCTTCAGCATCTTTTACTTTCACCAGCGTTTCTGGGTGAGCA AAAACAGGAAGGCAAAATGCCGCAAAAAAGGGAATAAGGGCGACACGGAA ATGTTGAATACTCATACTCTTCCTTTTTCAATATTATTGAAGCATTTATC AGGGTTATTGTCTCATGAGCGGATACATATTTGAATGTATTTAGAAAAAT AAACAAATAGGGGTTCCGCGCACATTTCCCCGAAAAGTGCCACCTGACGT CTAAGAAACCATTATTATCATGACATTAACCTATAAAAATAGGCGTATCA CGAGGCCCTTTCGTCTCGCGCGTTTCGGTGATGACGGTGAAAACCTCTGA CACATGCAGCTCCCGGAGACGGTCACAGCTTGTCTGTAAGCGGATGCCGG GAGCAGACAAGCCCGTCAGGGCGCGTCAGCGGGTGTTGGCGGGTGTCGGG GCTGGCTTAACTATGCGGCATCAGAGCAGATTGTACTGAGAGTGCACCAT ATGCGGTGTGAAATACCGCACAGATGCGTAAGGAGAAAATACCGCATCAG GCGCCATTCGCCATTCAGGCTGCGCAACTGTTGGGAAGGGCGATCGGTGC GGGCCTCTTCGCTATTACGCCAGCTGGCGAAAGGGGGATGTGCTGCAAGG CGATTAAGTTGGGTAACGCCAGGGTTTTCCCAGTCACGACGTTGTAAAAC GACGGCCAGTGCC
